# Postglacial recolonization shaped the genetic diversity of the winter moth (*Operophtera brumata*) in Europe

**DOI:** 10.1002/ece3.2860

**Published:** 2017-04-01

**Authors:** Jeremy C. Andersen, Nathan P. Havill, Adalgisa Caccone, Joseph S. Elkinton

**Affiliations:** ^1^ Department of Environmental Conservation University of Massachusetts Amherst Amherst MA USA; ^2^ USDA Forest Service Northern Research Station Hamden CT USA; ^3^ Department of Ecology & Evolutionary Biology Yale University New Haven CT USA; ^4^ Present address: Jeremy C. Andersen, Department of Environmental Science Policy and Management University of California Berkeley Berkeley CA USA

**Keywords:** biogeography, gene flow, glacial refugia, hybrid zone, invasive pests, outbreak waves

## Abstract

Changes in climate conditions, particularly during the Quaternary climatic oscillations, have long been recognized to be important for shaping patterns of species diversity. For species residing in the western Palearctic, two commonly observed genetic patterns resulting from these cycles are as follows: (1) that the numbers and distributions of genetic lineages correspond with the use of geographically distinct glacial refugia and (2) that southern populations are generally more diverse than northern populations (the “southern richness, northern purity” paradigm). To determine whether these patterns hold true for the widespread pest species the winter moth (*Operophtera brumata*), we genotyped 699 individual winter moths collected from 15 Eurasian countries with 24 polymorphic microsatellite loci. We find strong evidence for the presence of two major genetic clusters that diverged ~18 to ~22 ka, with evidence that secondary contact (i.e., hybridization) resumed ~ 5 ka along a well‐established hybrid zone in Central Europe. This pattern supports the hypothesis that contemporary populations descend from populations that resided in distinct glacial refugia. However, unlike many previous studies of postglacial recolonization, we found no evidence for the “southern richness, northern purity” paradigm. We also find evidence for ongoing gene flow between populations in adjacent Eurasian countries, suggesting that long‐distance dispersal plays an important part in shaping winter moth genetic diversity. In addition, we find that this gene flow is predominantly in a west‐to‐east direction, suggesting that recently debated reports of cyclical outbreaks of winter moth spreading from east to west across Europe are not the result of dispersal.

## 1 Introduction

Changes in climate conditions have long been recognized to be a major driver of range expansion, and local adaptation (Elias, Faria, Gompert, & Hendry, 2012). This is particularly true for many species dwelling in temperate regions whose recent evolutionary histories have been shaped by changes in their distribution during the Quaternary climatic oscillations (see references in Rousselet et al., 2010), and continue to be shaped by anthropogenic climate change (Svenning, Eiserhardt, Normand, Ordonez, & Sandel, 2015). In the western Palearctic, numerous studies have shown that diversification has been promoted during periods when species’ distributions were restricted to geographically isolated glacial refugia in the southern peninsulas and/or alpine regions of the European continent (see reviews by Hewitt, 1996, 2000; and Schmitt, 2007). For contemporary populations, the effects of these periods of isolation are often observed by the presence of distinct genetic lineages that correspond with the numbers and locations of the glacial refugia utilized by a particular species (Taberlet, Fumagalli, Wust‐Saucy, & Cosson, 1998).

In addition to promoting patterns of diversification, the Quaternary climatic oscillations have also had profound impacts on genetic variation within populations. One common finding for postglacial recolonization studies is that populations in geographic regions that acted as glacial refugia during the glacial maximums are more genetically diverse than populations in regions that have been recolonized (i.e., the “southern richness, northern purity” paradigm; Hewitt, 2000; Canestrelli, Cimmaruta, Costantini, & Nascetti, 2006; Babik et al., 2009; Rousselet et al., 2010; Wielstra et al., 2013; Tison et al., 2014; Mezzasalma et al., 2015; Vitales, Garcia‐Fernandez, Garnatje, Pellicer, & Valles, 2016). As populations of species recolonized temperate regions, they likely extended their distributions in a stepping‐stone manner whereby multiple genetic bottleneck events were associated with continued northward range expansions (e.g., Tison et al., 2014). However, during these cycles genetic lineages that were reproductively isolated during glacial maximums might also have subsequently come into secondary contact with one another during periods of recolonization and the signatures of these secondary contact events have been shown by the presence of detectible hybrid zones (Hewitt, 2000; Schmitt, 2007; Schmitt & Müller, 2007). Hybridization—here used in the context of admixture between distinct populations (Harrison & Larson, 2014)—has long been known to play an important role in shaping patterns of host‐associations (e.g., Feder et al., 2003), and has the potential to greatly increase measures of genetic diversity (Verhoeven, Macel, Wolfe, & Biere, 2011) and even reverse the process of speciation (Seehausen, Takimoto, Roy, & Jokela, 2007). How hybridization might affect the “southern richness, northern purity” paradigm, however, is unclear. In part this may be because the strength of the signal of this paradigm may vary depending on the recolonization paths taken by a species, and the geographic locations of secondary contact zones (see Hewitt, 2000 and Schmitt, 2007).

Species of Lepidoptera have been crucial for examining both of the above biogeographic patterns. This is could be due to the fact that many species of Lepidoptera have the ability to disperse long distances and can quickly fill open niche space at the species‐level, while dispersal distances for individuals are generally quite small, allowing for the preservation of biogeographic structure (Schmitt, 2007). These short dispersal distances may also be important for the promotion of local adaptation (Tison et al., 2014), and recent studies of Lepidoptera have also highlighted the importance of micro‐refugia (von Reumont, Struwe, Schwarzer, & Misof, 2012), postcolonization hybridization (Schmitt & Müller, 2007), and colonization from refugia outside of Europe (Habel, Lens, Rodder, & Schmitt, 2011) for patterns of Eurasian biogeography. However, one species of Lepidoptera for which elucidating historical biogeographic patterns has been particularly elusive is the winter moth, *Operophtera brumata* (Lepidoptera: Geometridae). This species has a broad range of woody host plants including, but not limited to, oak (*Quercus*), maple (*Acer*), and birch (*Betula*) trees in Europe (Wint, 1983), and has long been studied as a model organism for studies of local adaptation (Tikkanen & Lyytikainen‐Saarenmaa, 2002; Tikkanen, Woodcock, Watt, & Lock, 2006; Van Dongen, Matthysen, & Dhondt, 1996), and population ecology (Hassell, 1968; Macphee, Newton, & McRae, 1988; Varley & Gradwell, 1960, 1968), and has been at the center of an ongoing debate in regards to whether cyclical outbreaks of geometrid moths (including winter moth) move across western Eurasian from east to west approximately every 10 years (Tenow et al., 2013; but see Jepsen, Vindstad, Barraquand, Ims, & Yoccoz, 2016 and Tenow, 2016). In addition, this species has also been the subject of much genetic research, including population structure (Leggett et al., 2011; Van Dongen, Backeljau, Matthysen, & Dhondt, 1998), hybridization (Elkinton, Liebhold, Boettner, & Sremac, 2014; Elkinton et al., 2010; Havill et al., 2017), and a draft genome for this species was recently published (Derks et al., 2015). Yet, the only continent‐scale study of winter moth phylogeography (Gwiazdowski, Elkinton, Dewaard, & Sremac, 2013) found little evidence for geographically distinct genetic lineages when the mitochondrial locus cytochrome oxidase I (COI) was analyzed—although it did find support for a division between northern (i.e., Norway, Scotland, and Sweden) and southern European (i.e., all other sampled locations) populations. This result was unexpected, given ample evidence of local adaptation by winter moth populations (Tikkanen & Lyytikainen‐Saarenmaa, 2002; Tikkanen et al., 2006; Van Dongen et al., 1996), and the fact that females are flightless and that males are considered poor dispersers (Van Dongen et al., 1996).

Therefore, we were interested if a recently developed set of 24 highly variable microsatellite loci (Havill et al., 2017) could be used to further elucidate patterns of genetic diversity for populations of winter moth. Specifically we were interested the following: (1) whether individual winter moths could be assigned to distinct genetic clusters using Bayesian clustering analyses and genetic distance methods and whether these genetic groupings corresponded with geographically distinct glacial refugia, (2) whether divergence times between contemporary populations in regions that likely acted as glacial refugia corresponded with isolation during the last glacial maximum (LGM), (3) whether winter moth populations display evidence of decreasing genetic diversity with increases in latitude, and (4) whether contemporary gene flow could be detected and whether the direction of gene flow can inform the debate about cyclical outbreaks of geometrid moths.

## 2 Materials and Methods

### 2.1 Study species and sampling strategy

Winter moth is broadly distributed across western Asia, Europe (Tenow et al., 2013; Troubridge & Fitzpatrick, 1993), and North Africa (Ferguson, 1978; Mannai, Ezzine, Nouira, & Ben Jamaa, 2015), and is a non‐native invasive pest in North America (reviewed in Elkinton et al., 2014; Elkinton, Boettener, Liebhold, & Gwiazdowski, 2015). Larvae of this species hatch in early spring, often just prior to or synchronized with budburst (Buse & Good, 1996; Tikkanen & Lyytikainen‐Saarenmaa, 2002; Tikkanen et al., 2006; Van Dongen, Backeljau, Matthysen, & Dhondt, 1997). Once emerged, larvae cause considerable damage to their hosts, which include a broad range of deciduous (Wint, 1983) and evergreen (Watt & Mcfarlane, 1991) trees and shrubs. After pupation in the leaf‐litter, flightless adult females and winged adult males emerge during November or December (in most regions) and females attract mates using a sex pheromone (Roelofs et al., 1982) before depositing between 150–350 eggs that overwinter until the following spring (Elkinton et al., 2015).

For this study, male moths were collected at 44 locations in Eurasia (Table S1, Figure S1) using sex pheromone‐baited traps deployed in tree canopies that attract winter moth and several closely related congeners (Elkinton, Lance, Boettner, Khrimian, & Leva, 2011; Elkinton et al., 2010). Moths were collected from the traps and then placed in glassine envelopes (Uline Corporation, USA), before storage at −80°C.

### 2.2 DNA extraction and amplification

Whole genomic DNA was extracted from individual male winter moths using the Qiagen DNeasy Kit (Qiagen Corporation, Netherlands) following the manufacturer's instructions. Prior to DNA extraction, the tip of the abdomen (genitalia are used for morphological species identification) and wings were removed and stored as vouchers for most of the samples. Vouchers were deposited at the Yale Peabody Museum of Natural History, New Haven Connecticut, USA. Twenty‐four polymorphic microsatellites were then amplified from each individual using primers and protocols presented in Havill et al. (2017). Genotyping was conducted at the DNA Analysis Facility on Science Hill at Yale University using a 3730xl DNA Analyzer (Thermo Fisher Scientific, USA), and fragment lengths were scored in comparison with the GeneScan 500 LIZ size standard (Thermo Fisher Scientific) using the microsatellite plug‐in in the software program geneious v.8.1.6 (Kearse et al., 2012).

### 2.3 Winter moth postglacial recolonization

After genotyping, individuals from which ≥20 microsatellite loci were successfully amplified were included in subsequent analyses. Genotype data for each included individual is provided as a single‐line formatted file titled (Appendix S2).

#### 2.3.1 Bayesian genetic clustering

Previous postglacial recolonization studies have found that the Quaternary climatic cycles have played an important role in shaping genetic diversity. This diversity is evident by the presence of two to three distinct genetic lineages in Eurasia (Hewitt, 2000; and Schmitt, 2007), with well‐established hybrid zones where these lineages have come into secondary contact with each other (see Hewitt, 2000; Figure 3). Preliminary analyses of our dataset using structure v.2.3.2 (Falush, Stephens, & Pritchard, 2003; Pritchard, Stephens, & Donnelly, 2000) indicated that individual winter moths could be assigned to an optimal number of two distinct genetic clusters (Appendix S1). Therefore, we estimated the probability of assignment (*Z*) that an individual could be classified as belonging to one of these two clusters or to hybrid categories (F1, F2, or backcrosses), using the software program newhybrids v.1.1.b3 (Anderson, 2008; Anderson & Thompson, 2002). Four independent runs, each of 1 million generations, discarding the first 100,000 burn‐in generations, were analyzed using random starting values, and uniform priors for the estimates of θ (i.e., allele frequencies) and π (i.e., mixing proportions). Results were then averaged across runs, and visualized using arcMap v.10.3.1 (Esri, Redlands, CA, USA).

#### 2.3.2 Population genetic statistics and genetic distances

For all localities from which ≥10 individuals were successfully genotyped, we calculated standard population genetic summary statistics, including: the number of individuals genotyped (*n*), the average number of alleles per locus (*N*_*a*_), the effective number of alleles (*Eff_N*_a_), the average observed heterozygosity across loci (*H*_o_), the average inbreeding coefficient (*G*_IS_), and the presence of deviation from Hardy–Weinberg Equilibrium (HWA) using genodive v.2.0b27 (Meirmans & Van Tienderen, 2004). In addition, we tested each locus‐pair within populations for evidence of Linkage‐Disequilibrium (LD) using genepop (Raymond & Rousset, 1995; Rousset, 2008), and we estimated the frequency of null alleles present for each locus and population using freeNA (Chapuis & Estoup, 2007). The degree of genetic differentiation (*F*_ST_) among populations was calculated (accounting for the presence of null alleles) in freeNA, and Jost's estimator of actual differentiation (*D*_est_) was calculated using smogd v.1.2.5 (Crawford, 2010). To examine whether genetic differentiation was correlated with geographic distances, the presence of isolation‐by‐distance was estimated by performing linear‐regression of *F*_ST_ / (1‐*F*_ST_), calculated in genepop, versus the linear distance between populations (km) in R v. 3.1.3 (R Core Team 2015). Finally, to examine historical relationships among populations a “NeighborNet” network was reconstructed with the above null‐allele corrected *F*_ST_ estimates with the program splitstree v.4.14.2 (Huson & Bryant, 2006).

#### 2.3.3 Historical demography

To determine whether divergence timing for contemporary Eurasian populations of winter moth correspond with genetic isolation during the LGM, population history parameters were estimated using Approximate Bayesian Computation (ABC) with the software program diyABC v.2.1.0 (Cornuet et al., 2008, 2014). For these analyses we included samples from Spain, Serbia, and Georgia as representative of three potential glacial refugia (Iberaian, Balkan, and Caucasus, respectively). Due to limited sampling from Italy (Italian refugium), we did not include samples from this region in our analysis. In addition, we included samples from Germany to represent the product of postglacial recolonization. We evaluated eight different recolonization scenarios (Figure S2). Three scenarios included simple bifurcating trees that differed in whether the German population was derived from the Spanish, Serbian, or Georgian populations, and five scenarios that included the German population being derived from admixture. All scenarios included multiple population size parameters to allow for changes in population sizes following splitting and/or merging events. Prior information for all parameters, including minimum and maximum values, as well as distributions, is presented in Table S2. Default mutation model parameters were used, except for the following: we set the minimum mean mutation rate to 1 × 10^−5^, and maximum values for the Mean and Individual locus coefficient P's to 1.0. Visualization of preliminary results based on principal component analyses (PCAs) within diyABC indicated that these changes in the mutation rate and the Mean and Individual locus coefficient P's improved the shape of the cloud of simulated datasets. In addition, visualization of preliminary results based on PCAs indicated that the removal of four loci (02339, 00925, 02191, and 12042) also improved the shape of the cloud of simulated datasets. These four loci each had allelic ranges ≥120 repeat units (i.e., the number of tandem repeats). Given that lepidopteran genomes have high rates of duplication events (Edger et al., 2015; You et al., 2013), and that the development of microsatellite loci for these taxa have been difficult due to high frequencies of null alleles and associations with transposable elements (see citations in Sinama et al., 2011), it is possible that there might be an association between these four loci and transposable elements and/or duplication events. We will continue to monitor the appropriateness of using these four loci. We then generated a reference table by simulating 8 million datasets (1 million datasets per scenario). Pre‐evaluations of the simulated datasets were conducted using PCA, and scenarios were compared using both the “Direct” (i.e., the proportion of simulated datasets from each scenario “closest” to the sample dataset) and “Logistic Regression” (i.e., a logistic regression analysis of the probability of deviations between summary statistics calculated for the simulated datasets and the sample dataset) tests in diyABC.

#### 2.3.4 Genetic diversity and latitude

To examine whether measures of genetic diversity (*N*_*a*_, *Eff_N*_a_, *H*_o_, and *G*_IS_) were correlated with latitude, we constructed generalized additive models (GAM) using the R package “mgcv” (Wood, 2011). Results were then visualized using the “plot.gam” function.

### 2.4 Contemporary rates of gene flow

The rates of contemporary gene flow among populations in Eurasia were examined by estimating the proportion of migrant individuals in each population using the software program bayesass v.4.0 (Wilson & Rannala, 2003). To reduce the number of pair‐wise comparisons, individuals were grouped by country. Gene flow rates were then estimated for all 205 possible comparisons with four independent analyses, each with a random starting seed, runtimes of 10 million generations, burn‐in periods of 1 million generations, and by setting all mixing parameters to 0.8. Results were then summarized across analyses using tracer v.1.6.0 (Rambaut & Drummond, 2007), and all migration rates whose 95% confidence intervals (*mean* ± [1.96 × *SE*]) did not include 0 are reported as significant. Average rates of gene flow between eastern (Austria, Czech Republic, Georgia, Poland, Serbia, Slovakia) and western (England, France, Germany, Italy, Norway, Scotland, Spain, Sweden, Switzerland) European countries were then calculated. The difference between these rates was used as the basis for simulations to determine if one or the other populations would eventually become ubiquitous and if so in how many generations. Simulations were carried out using the software program popG v. 4.03 (© University of Washington and Joseph Felsenstein), under a simple two allele model with equal starting frequencies and the observed difference in migration rates. Ten independent simulations were conducted; each with random starting seeds, and the number of generations to fixation of one of the alleles averaged across runs was recorded.

## 3 Results

### 3.1 Winter moth postglacial recolonization

#### 3.1.1 Bayesian genetic clustering

**Figure 1:**
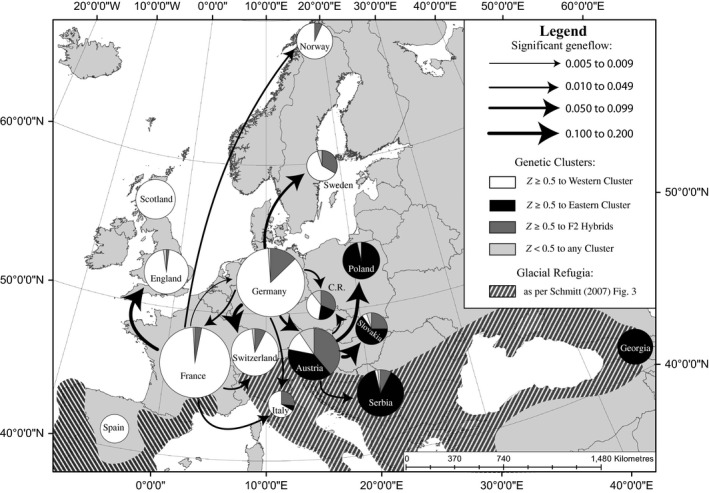
Proportional assignment of individuals to hybrid categories, and contemporary gene flow rates among Eurasian countries. Pie charts, representing the proportion of individuals in each country assigned to different hybrid categories calculated with newhybrids (*Z *≥* *0.5 to Cluster 1 =  Black; *Z* ≥ 0.5 to Cluster 2 = White; *Z *≥* *0.5 to F2 Hybrid = Dark Gray; *Z *< 0.5 to any category = Light Gray), were generated in arcMap v.10.3.1 and visualized using the Europe Albers Equal Area Conic projection. The location of each chart is placed approximately in the center of each country. The label for the Czech Republic has been abbreviated “C.R.” Gene flow rates are presented as the proportion of individuals that are likely migrants from a different Eurasian country as calculated by bayesass. The thickness of the curved lines represents the proportion of migrants (see legend, top right), and the arrowhead indicates the direction of gene flow. The distribution of glacial refugia during the LGM (diagonal shading) is drawn as per Figure 3 in Schmitt (2007)

After filtering the dataset to include only those individuals from which ≥20 microsatellite loci were genotyped, analyses included 669 individual moths. The proportional assignment of individuals by newhybrids is presented in Table S1 and summarized by country in Figure 1. These results showed that 145 individuals could be assigned to Cluster 1 (123 individuals with high probability of assignment *Z ≥ *0.8, and 22 individuals with moderate probabilities of assignment 0.5 ≤ *Z *< 0.8). Four hundred and thirty‐four individuals were assigned to Cluster 2 Cluster 2 (386 with high probability of assignment and 47 with moderate probability of assignment). In addition, 78 individuals were identified as hybrids (13 with high probability of assignment and 65 with moderate probabilities of assignment). All hybrids were assigned to the F2 hybrid class. Thirteen individuals were classified as “unassigned” due to low probabilities of assignment (*Z < *0.5 to any category). In general, assignments showed a clear distinction between eastern and western Europe, with moths from Georgia, Poland, and Serbia being primarily assigned to Cluster 1 (100%, 97%, and 88%, respectively), and individuals from Spain, Scotland, and England being primarily assigned to Cluster 2 (100%, 100%, and 96%, respectively). In addition, while the overall number of hybrids was low, the greatest proportions of hybrid individuals were found in Austria, Sweden, and the Czech Republic (39%, 33%, and 32%, respectively), roughly corresponding with a previously identified hybrid zone in central Europe (see Figure 3 in Hewitt, 2000).

#### 3.1.2 Population genetic statistics and genetic distances

Population genetic statistics for 26 locations from which ≥10 individual moths were sampled showed that the average number of alleles per locus was *N*_*a *_= 8.63, the average effective number of alleles per locus was *Eff_N*_*a*_ = 4.34 the average observed heterozygosity was *H*_o_ = 0.58, and the average inbreeding coefficient was *G*_IS_ = 0.17 (Table 1). The average presence of null alleles across loci was 6.7%. All locations showed significant (*p* ≤ .05) deviations from HWE, while only 13 of the 276 pair‐wise comparisons showed significant (*p* ≤ .05) evidence of LD between loci after using the Benjamini–Hochberg false‐discovery method (Benjamini & Hochberg, 1995).

**Table 1:** Population genetic summary statistics calculated using genodive, including: the number of individuals genotyped (*n*), the average number of alleles per locus (*N*_*a*_), the effective number of alleles (*Eff_N*_*a*_), the average observed heterozygosity (*H*_o_)*,* the inbreeding coefficient (*G*_IS_), the probability (*p*) of deviation from Hardy–Weinberg Equilibrium (HWE), and the percent of individuals in the population classified as hybrids using newhybrids (%Hybrids)

Population	*n*	*N*_*a*_	*Eff_N*_*a*_	*H*_*o*_	*G*_IS_	HWE	%Hybrids
Austria, Neulengbach	29	10.125	4.841	0.610	0.167	0.001	37.93
Austria, Vienna	35	10.625	4.802	0.623	0.113	0.001	40.00
Czech Republic, Prague	19	8.083	4.505	0.535	0.251	0.001	31.58
England, Farnham	48	10.583	4.149	0.566	0.185	0.001	2.08
France, Bédarieux	16	7.042	3.966	0.579	0.183	0.001	0.00
France, Haguenau	31	9.292	4.451	0.619	0.145	0.001	12.90
France, Marcillac	29	10.500	4.876	0.645	0.128	0.001	0.00
France, Onet L'eglise	20	6.875	3.732	0.481	0.270	0.001	0.00
France, Rennes	23	8.458	4.334	0.587	0.156	0.001	0.00
Georgia, Tbilisi	27	7.500	3.519	0.519	0.117	0.001	0.00
Germany, Bühren	30	10.500	4.695	0.614	0.170	0.001	13.33
Germany, Göttingen	30	10.000	4.803	0.633	0.143	0.001	16.67
Germany, Reinhardshagen	30	10.375	4.694	0.668	0.111	0.001	13.33
Germany, Schlüchtern	18	8.542	4.494	0.597	0.181	0.001	11.11
Italy, Tregnago	15	7.083	4.041	0.531	0.242	0.001	15.38
Norway, Tromsø	29	9.125	4.469	0.601	0.173	0.001	6.67
Poland, Bialostocka	20	9.292	5.106	0.608	0.152	0.001	0.00
Scotland, Banchory	10	5.042	2.89	0.539	0.089	0.011	0.00
Scotland, Torphins	30	7.917	3.459	0.526	0.160	0.001	0.00
Serbia, Belgrade	15	7.208	4.471	0.558	0.197	0.001	6.67
Serbia, Pančevo	37	11.792	6.11	0.570	0.218	0.001	8.11
Slovakia, Banská Štiavnica	20	8.542	4.665	0.537	0.248	0.001	25.00
Spain, Lugo	16	4.708	2.583	0.448	0.138	0.001	0.00
Sweden, Uppsala	21	8.792	4.69	0.645	0.145	0.001	33.33
Switzerland, Delémont	21	8.250	4.176	0.651	0.086	0.001	4.76
Switzerland, Malettes	19	8.208	4.179	0.625	0.133	0.001	10.53

**Figure 2:**
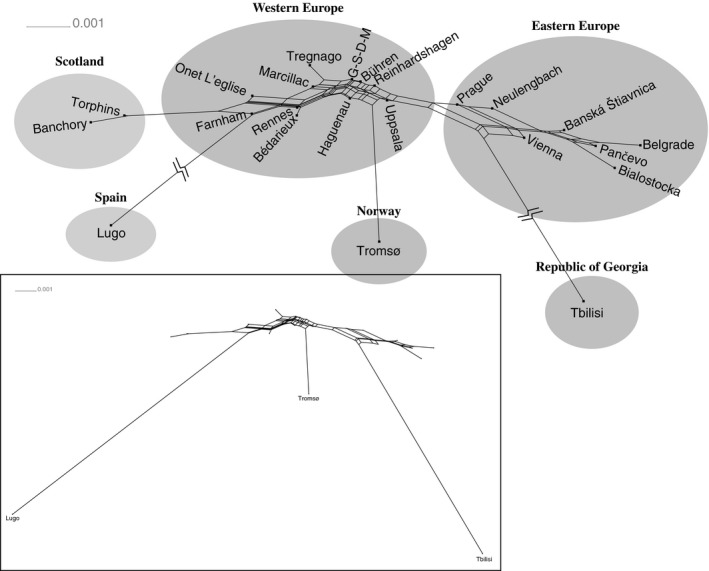
Network Analysis based on *F*_ST_ (corrected for the presence of null alleles). Branches are drawn proportional to the scale bar in the upper left corner. Branches leading to Tbilisi, Georgia and Lugo, Spain have been “broken” in the main figure, but for reference these branches are shown intact in the inset figure. Four sampled locations, Göttingen, Schlüchtern, Delémont, and Malettes had distances of *F*_ST_ = 0.00, and the node with these four populations is indicated with the label “G‐S‐D‐M”

Among populations, the average values for pair‐wise differentiation for *F*_ST_ (corrected for the presence of null alleles) was 0.060 and for *D*_est_ was 0.091 (Table S3). Genetic differentiation also showed strong patterns of isolation‐by‐distance (*p* = 2.2 × 10^−16^, *r* ^2 ^= .5235, *df* = 323) among sampled populations. Network analysis indicated that most populations could be divided into two large subnetworks (Figure 2). One of these subnetworks included populations from Austria, Czech Republic, Poland, Serbia, and Slovakia (labeled “Eastern Europe”), while the other subnetwork included populations from England, France, Germany, Italy, and Switzerland (labeled “Western Europe”). In addition, populations of moths from Scotland, Spain, Norway, and the Republic of Georgia were each placed at the ends of long branches.

#### 3.1.3 Historical demography

**Figure 3:**
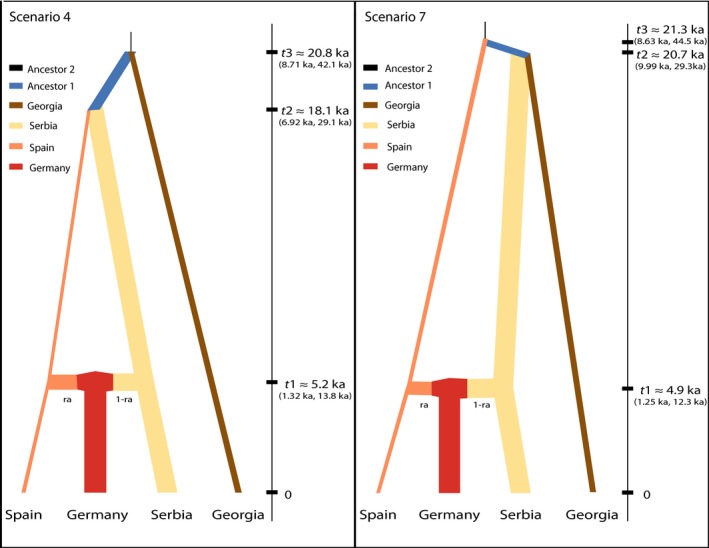
The top two scenarios for diversification and recolonization identified by simulations in diy ABC. Populations in each scenario were chosen to represent different glacial refugia (e.g., Spain = Iberian Refugium, Serbia = Balkan Refugium, Georgia = Caucasus Refugium), as well as a population that would have established after the northward retreat of the glaciers following the LGM (e.g., Germany). Both of these supported scenarios include an admixture event between the Serbian and Spanish populations giving rise to the German population. Mean estimates for merging (t1) and divergence (t2 and t3) events are shown along the *y*‐axis along with 95% confidence intervals. The thicknesses of branches are drawn proportional to the mean estimated population size. Estimates for all parameters, including 95% confidence intervals, are presented in Table 2. All eight tested scenarios is show in Figure S2

Comparisons of the posterior probabilities of the scenarios included in the diyABC analysis indicated that Scenarios 4 and 7 were the best fit for the data (Figure S3). Under the Direct approach simulations based on Scenario 7 were consistently the most supported, whereas under the Logistic Regression approach support moves from Scenario 4 to Scenario 7 with increasing distance from the sample data (Figure S3). These two scenarios differ in regards to whether the Serbian population is sister to the Georgian or the Spanish populations, yet both scenarios indicate that the German populations are derived from recent admixture between the populations that recolonized Europe from the Iberian and Balkan refugia. The branching pattern observed in Scenario 7 also matches the results observed from the Bayesian clustering and distance analyses (i.e., Georgia being most closely related to eastern European populations). Results for all estimated parameters for these two scenarios are presented in Table 2, and the mean values for divergence times and population sizes are presented graphically in Figure 3. In both scenarios, the German and Serbian effective population sizes are significantly larger (non‐overlapping 95% CIs) than the Georgian and Spanish populations. The mean estimates for population sizes were all larger than the estimates for the ancestral population 2 (NA5), although again these were only significant for the German and Serbian populations, and there were no significant differences between the ancestral population 1 (NA3) and any contemporary populations suggesting that population sizes have remained somewhat stable through time. In both scenarios 4 and 7, the mean estimate for when admixture between the Spanish and Serbian populations occurred was ~5 ka (Scenario 4 = 5.19 ka [95% CI = 1.32 ka, 13.8 ka]; Scenario 7 = 4.9 ka [95% CI = 1.25 ka, 12.3 ka]), and the mean estimates for the divergence time between the eastern and western populations was ~ 20 ka (Scenario 4 t2 = 18.1 ka [95% CI = 6.82 ka, 29.1 ka]; Scenario 7 t3 = 21.3 ka [95% CI = 8.63 ka, 44.5 ka]). In both scenarios, the German population is derived from near equal proportions of admixture between the Spanish and Serbian populations (ra = 0.462 [95% CI = 0.047, 0.94] and ra = 0.412 [95% CI = 0.065, 0.866] for scenarios 4 and 7, respectively).

**Table 2:** Parameter estimates (mean and 95% Confidence Intervals [CI]) from diyABC for Scenarios 4 and 7 based on 1 million simulated datasets each. These estimates include; current effective population sizes in Georgia (N1), Serbia (N2), Spain (N3), and Germany (N4), as well as effective population sizes for ancestral population 1 (NA3) and ancestral population 2 (NA5). In addition, estimates are shown for the proportion of admixture (ra), time of divergence (t1, t2, and t3); as well as the average mutation rates (μ‐mic), the average coefficient of P (p‐mic), and the average SNI rates (sni‐mic) for the microsatellite loci

	Scenario 4			Scenario 7		
	Mean	95% CI Lower	95% CI Upper	Mean	95% CI Lower	95% CI Upper
Georgia	1.53E+04	7.24E+03	2.77E+04	1.85E+04	9.46E+03	2.97E+04
Serbia	5.62E+04	3.11E+04	8.60E+04	6.20E+04	3.68E+04	8.91E+04
Spain	1.14E+04	5.31E+03	2.11E+04	1.20E+04	5.32E+03	2.30E+04
Germany	6.75E+04	3.98E+04	9.32E+04	6.52E+04	3.96E+04	9.17E+04
Ancestral population 1	3.14E+04	2.27E+03	7.57E+04	2.02E+04	9.40E+02	7.05E+04
Ancestral population 2	2.75E+03	7.74E+01	8.86E+03	3.35E+03	1.08E+02	9.36E+03
ra	4.62E‐01	4.73E‐02	9.40E‐01	4.12E‐01	6.52E‐02	8.66E‐01
t1	5.19E+03	1.32E+03	1.38E+04	4.90E+03	1.25E+03	1.23E+04
t2	1.81E+04	6.82E+03	2.91E+04	2.07E+04	9.99E+03	2.93E+04
t3	2.08E+04	8.71E+03	4.21E+04	2.13E+04	8.63E+03	4.45E+04
μ‐mic	1.19E‐04	3.38E‐05	2.52E‐04	1.25E‐04	3.46E‐05	2.96E‐04
p‐mic	6.44E‐01	2.34E‐01	9.75E‐01	6.28E‐01	2.26E‐01	9.71E‐01
sni‐mic	1.06E‐07	1.00E‐08	7.70E‐07	1.15E‐07	1.00E‐08	8.49E‐07

#### 3.1.4 Genetic diversity and latitude

Results from the GAM analyses found no significant relationships between measures of genetic diversity (*N*_*a*_, GIS, and *H*_*o*_) and latitude. However, *Eff_N*_*a*_ and latitude showed a marginally significant relationship (*p* = .0838, *r* ^2^ = .361, deviance explained = 55.3%; Figure S4).

### 3.2 Contemporary rates of gene flow

Included in the gene flow analyses were all individuals from Austria (n = 64), Czech Republic (*n* = 19), England (*n* = 48), France (*n* = 124), Georgia (*n* = 27), Germany (*n* = 108), Italy (*n* = 15), Norway (*n* = 30), Poland (*n* = 29), Scotland (*n* = 40), Serbia (*n* = 52), Slovakia (*n* = 20), Spain (*n* = 20), Sweden (*n* = 21), and Switzerland (*n* = 52). Visual inspection of the four bayesass runs using tracer indicated that after 10 million generations all runs had converged on similar estimates for migration rates. After summarizing across runs, 15 of 205 possible migration routes showed evidence of significant gene flow (i.e., 95% CIs did not include 0) between populations (Table S4; Figure 1). Germany was the country with the highest number of significant connections, being the source of migrants to six other countries, as well as the recipient of migrants from France. Austria and France each had five connections (each with four sources and one recipient interaction), while Spain, Scotland, and Georgia showed no evidence of contemporary gene flow. Only Germany and France showed evidence of reciprocal gene flow, although gene flow was greater from Germany to France than the reverse. Of the 15 connections, 13 of them were directed in an easterly direction (e.g., east, north‐east, south‐east, etc.), while only two were directed in a westerly direction (Figure 1). The average dispersal rate from western to eastern countries was *m *=* *0.0125, while for eastern to western countries it was *m *=* *0.0070, with a difference in the average rate of migration of *m *=* *0.0055. Simulations run in popG using the observed difference in migration rates found the average time to fixation was 7,000 generations (±3,830 generations).

## 4 Discussion

### 4.1 Winter moth postglacial recolonization

We find that populations of winter moth can be broadly assigned to two geographically and genetically distinct populations (Figure 1), which diverged ~ 20 ka (Figure 3). While this finding differs slightly from previous evidence that suggested northern winter moth populations might be distinct from southern populations (Gwiazdowski et al., 2013), the observed results are consistent with many previous studies that have highlighted the importance of the Quaternary climate cycles in shaping patterns of genetic diversity and geographic distributions for other lepidopteran species in Europe and western Asia (e.g., Hewitt, 2000; Schmitt, 2007; Schmitt, Rober, & Seitz, 2005; Schmitt & Seitz, 2001a,b; Wu et al., 2015).

For winter moth, our findings suggest that populations became reproductively isolated in glacial refugia located both in the Iberian and Balkan peninsulas and in the Caucasus region, as a result of the expansion of the glaciers to their glacial maximums ~ 26 ka (Clark et al., 2009). During this period, populations in the Balkan Peninsula and the Caucasus evolved independently from populations in the Iberian Peninsula as evident by the level of genetic divergence observed between contemporary populations (Average *F*_ST_ = 0.060). This level of divergence is similar to that which has been observed for other species of Lepidoptera (e.g., Schmitt & Seitz, 2001a; *F*_ST_ = 0.060), but less than that which has been observed for species with recognized subspecies (e.g., Schmitt & Seitz, 2001b; *F*_ST_ = 0.149). Beginning ~20 ka the glaciers began to retreat (Clark et al., 2009), and during the subsequent reforestation of the European continent, these two lineages expanded northward, coming into secondary contact ~ 5 ka along a well‐documented hybrid zone in Central Europe (see Figure 3 in Hewitt, 2000).

Following the northward recolonization of the European continent, populations in Spain and Georgia—regions that acted as glacial refugia during the last glacial cycle—likely became reproductively isolated in these regions as evident by the long branches connecting these populations to other European populations (Figure 2). For the Spanish population, this result might highlight the importance of physical barriers, such as the Pyrenees, in promoting reproductive isolation, as seen for the pine processionary moth, *Thaumetopoea pityocampa* (Rousselet et al., 2010). Similarly, the distance of the branch leading to the Georgian population might also highlight the importance of geographic isolation, a pattern similar to that found for white oaks (Dumolin‐Lapègue, Demesure, Fineschi, LeCorre, & Petit, 1997), one of the primary hosts for winter moth. The Caucasus region has long been recognized as having played an important role as a refugium during glacial cycles (e.g., Babik et al., 2005; Beck, Schmuths, & Schaal, 2007; Dubey, Zaitsev, Cosson, Abdukadier, & Vogel, 2006; Hampe, Arroyo, Jordano, & Petit, 2003; Jaarola & Searle, 2002), however the area connecting the Caucasus region to continental Europe has gone through significant changes in forest cover since the last glacial maximum (Adams, 1997), and it is possible that these changes eventually prevented gene flow between winter moth populations in eastern Europe and the Caucasus region.

Genetic distance analyses also revealed that populations from two countries, Norway and Scotland, that were recolonized after the retreat of the glaciers showed additional evidence of genetic differentiation (Figure 2). In northern Norway, winter moth recently became established (~ 125 ya), and its introduction has caused extensive defoliations due to frequent outbreaks (Vinstad et al., 2013). Our gene flow analyses (Figure 1) indicated the presence of significant gene flow from populations in France to this region, suggesting that France might be the source of this invasive population. If this is correct, then the long branch linking the Norwegian population to the other western European populations (Figure 2) may be the result of a genetic bottleneck created during the invasion process. However, further sampling of the Fennoscandia region is required to comment further on the origins of this population. Similarly, the long branch connecting the populations of winter moth in Scotland to the other Western European populations is also interesting given that populations in Scotland have become a serious pest of Sitka spruce (Hunter, Watt, & Docherty, 1991; Stoakley, 1985), suggesting that this long branch might be the result of micro‐evolutionary processes as a result of this significant host shift.

#### 4.1.1 Genetic diversity and latitude

One common finding for postglacial recolonization studies is that populations in geographic regions that acted as glacial refugia during the LGM are more genetically diverse than populations in recolonized regions (Babik et al., 2009; Canestrelli et al., 2006; Hewitt, 2000; Mezzasalma et al., 2015; Rousselet et al., 2010; Tison et al., 2014; Vitales et al., 2016; Wielstra et al., 2013). Here we find some evidence that measures of genetic diversity might be greater in middle European latitudes (Figure S4), a region that is experiencing secondary contact (i.e., hybridization; Figure 1). Given that hybridization has the potential to provide populations with additional genetic diversity, and that rates of hybridization are much higher than previously suspected (Allendorf, Leary, Spruell, & Wenburg, 2001; Harrison & Larson, 2014) particularly among insect taxa (Schwenk, Brede, & Streit, 2008), secondary contact following postglacial recolonization may play an important, and underreported, role in promoting the genetic diversity of populations in western Eurasia.

### 4.2 Contemporary rates of gene flow

Another interesting finding from our study involves the level and direction of gene flow among contemporary European populations. Recent studies have explored whether outbreaks of winter moth occur on a cyclical basis, suggesting a cycle length of approximately 10 years during which winter moths move across Europe predominantly from east to west (Tenow et al., 2013; but see Jepsen et al., 2016 and Tenow, 2016). These studies have been important contributions to our understanding of the incidence and causes of cyclic population dynamics and of damaging insect outbreaks, however the factors responsible for these outbreaks remain unclear. Dispersal may be an important driver of outbreaks of herbivorous insects (Liebhold, Koenig, & Bjornstad, 2004; Peltonen, Liebhold, Bjornstad, & Williams, 2002), although it is but one of numerous biotic and abiotic factors that can be responsible for the observed patterns (Hunter & Elkinton, 2000; Price & Hunter, 2015; Riolo, Rohani, & Hunter, 2015). Previous studies have highlighted the ability of winter moth populations to undergo long‐distance dispersal (Leggett et al., 2011), likely through a process known as ballooning where larvae secrete a silky thread and are subsequently wind‐dispersed (Elkinton et al., 2015). Therefore, long‐distance dispersal by larvae may provide a mechanism that drives these outbreak waves. If this were the case, then we might expect levels of gene flow to be primarily in the east to west direction (the proposed direction of the outbreak waves), however; our results suggest that long‐distance dispersal occurs primarily in the opposite direction (from west to east). In addition, if dispersal were responsible for these cyclical outbreaks, such frequent dispersal events would have long since obliterated the genetic distinction we discovered between eastern and western populations resulting in the predominance of the eastern genetic lineage. In contrast, our simulation results suggest that the western population will become predominant after another ~2,000 years of continued secondary contact, although populations of the eastern genetic lineage might likely still persist in the Caucasus region due to geographic isolation.

## Data Accessibility

Microsatellite genotype scores for each individual are provided as a tab‐delimited file in single line structure format. See Appendix S2.

## Author Contributions

JSE coordinated the collection of samples and directed the research. AC provided laboratory access and oversaw the analyses. JCA and NPH collected the molecular data. JCA analyzed the dataset and oversaw manuscript preparation. All authors contributed to the project design and in preparing the manuscript.

## Conflict of Interests

The authors declare no conflict of interests.
