# Supplementary material for: Postglacial recolonization shaped the genetic diversity of the winter moth (Operophtera brumata) in Europe
Source: Ecol Evol. 2017 Apr 1;7(10):3312–23. doi: 10.1002/ece3.2860 (PMC5433974; doi:10.1002/ece3.2860)
Supplement: Supplementary file 1 [file ECE3-7-3312-s001.docx]

**Appendix S1**

**Supplemental Materials and Methods**

*Genetic Clustering*

The probability of assignment (*Q*) of individuals to a given number of genetic clusters (*K*) was examined using Structure v.2.3.2 (Pritchard *et al.* 2000; Falush *et al.* 2003). Ten independent runs, each of 1 million generations, discarding the first 100,000 burn-in generations, were analysed for *K=*1 through *K=*6. Results from independent runs for each value of *K* were combined and summarized using Clumpp v.1.1.2 (Jakobsson & Rosenberg 2007), and the optimal value for *K* was estimated using the approach of Evanno *et al.* (2005) as implemented in Structure Harvester v.0.6.94 (Earl & vonHoldt 2012).

Genetic clustering of individuals was further examined using Discriminate Analysis of Principle Components (DAPC) (Jombart *et al.* 2010), as implemented through the R v. 3.1.3 (R Core Team 2015) package ‘adegenet’ (Jombart 2008; Jombart & Ahmed 2011). The above Structure dataset was further filtered to remove all individuals with missing data to allow for cross-validation and estimation of the ideal number of principle components (PCs) and discriminant factors to retain, as performed through the “xvalDapc” command. The optimal number of PCs and discriminant factors were then used to conduct the DAPC analysis, and the results were visualized as a scatter plot.

**Results**

The results from StructureHarvester indicated that the optimal number of genetic clusters was *K*=2 (Table S5), though log-likelihood scores continued to slightly improve for increasing values of *K* (Fig. S5). The proportional assignment of individuals to each genetic cluster is reported in Table S1, in Fig. S6, and summarized in Fig. S1.

After removal of individuals with missing data, 318 individuals were included in the DAPC analysis. Following cross validation, 40 PCs and 14 discriminant factors were retained. The scatter plot (Fig. S7) supports the longitudinal gradient observed in the NewHybrids and Structure analyses (Fig. 1, Fig. S1, Fig. S5), though it differs in that moths from Georgia formed a distinct genetic cluster (Fig. S7, top left quadrant).

**Supplemental Information References**

Earl DA, vonHoldt BM (2012) STRUCTURE HARVESTER: a website and program for visualizing STRUCTURE output and implementing the Evanno method. *Conservation Genetics Resources*, **4**, 359-361.

Evanno G, Regnaut S, Goudet J (2005) Detecting the number of clusters of individuals using the software STRUCTURE: a simulated study. *Molecular Ecology*, **14**, 2611-2620.

Falush D, Stephens M, Pritchard JK (2003) Inference of population structure using multilocus genotype data: Linked loci and correlated allele frequencies. *Genetics*, **164**, 1567-1587.

Jakobsson M, Rosenberg NA (2007) CLUMPP: a cluster matching and permutation program for dealing with label switching and multimodality in analysis of population structure. *Bioinformatics*, **23**, 1801-1806.

Jombart T (2008) adegenet: a R package for the multivariate analysis of genetic markers. *Bioinformatics*, **24**, 1403-1405.

Jombart T, Ahmed I (2011) adegenet 1.3-1: new tools for the analysis of genome-wide SNP data. *Bioinformatics*, **27**, 3070-3071.

Jombart T, Devillard S, Balloux F (2010) Discriminant analysis of principal components: a new method for the analysis of genetically structured populations. *BMC Genetics*, **11**, 94

Pritchard JK, Stephens M, Donnelly P (2000) Inference of population structure using multilocus genotype data. *Genetics*, **155**, 945-959.

**Supplemental Tables**

**Table S1.** Locality information including Latitue, Longitude, Date of Collection, and Collector for each sample. In addition, the Structure and GenePop formatted population codes (StructurePop, and GenePop, respectively), the probability of assignment (*Q*) to one of two genetic clusters using Structure (Q_Pop1 and Q_Pop2), the Assignment given to that individual (as displayed in Fig. S1), the probability of assignment (*Z*) to one of two genetic clusters using NewHybrids (Z_Pure1 and Z_Pure2), F1 hybrids (Z_F1), F2 hybrids (Z_F2), backcrosses to population 1 or 2 (Z_BC1 and Z_BC2, respectively), and the assignment given to that individual (as displayed in Fig. 1).

| **Table S1 Locality information, genotype scores, and probabilities of assignment** | | | | | |  |  |  |  |  |  |  |  |  |  |  |  |  |
| --- | --- | --- | --- | --- | --- | --- | --- | --- | --- | --- | --- | --- | --- | --- | --- | --- | --- | --- |
| **Country** | **Location** | **Latitude** | **Longitude** | **Date** | **Collector** | **Sample** | **StructurePop** | **GenePop** | **Q_Pop1** | **Q_Pop2** | **Structure Assignment** | **Z_Pure1** | **Z_Pure2** | **Z_F1** | **Z_F2** | **Z_BC1** | **Z_BC2** | **NH Assignment** |
| Austria | Neulengbach | 48.18333333 | 15.91 | Feb-07 | Gernot Hoch and Alex Schopf | 11-387-05 | 1 | 1 | 0.951 | 0.049 | Eastern | 0.963 | 0.000 | 0.000 | 0.028 | 0.009 | 0.000 | Eastern |
| Austria | Neulengbach | 48.18333333 | 15.91 | Feb-07 | Gernot Hoch and Alex Schopf | 11-387-06 | 1 | 1 | 0.966 | 0.034 | Eastern | 0.997 | 0.000 | 0.000 | 0.001 | 0.002 | 0.000 | Eastern |
| Austria | Neulengbach | 48.18333333 | 15.91 | Feb-07 | Gernot Hoch and Alex Schopf | 11-387-07 | 1 | 1 | 0.821 | 0.179 | Eastern | 0.271 | 0.007 | 0.019 | 0.650 | 0.035 | 0.017 | F2 - Moderate |
| Austria | Neulengbach | 48.18333333 | 15.91 | Feb-07 | Gernot Hoch and Alex Schopf | 11-387-08 | 1 | 1 | 0.653 | 0.347 | Mixed | 0.015 | 0.352 | 0.009 | 0.573 | 0.007 | 0.045 | F2 - Moderate |
| Austria | Neulengbach | 48.18333333 | 15.91 | Feb-07 | Gernot Hoch and Alex Schopf | 11-387-09 | 1 | 1 | 0.862 | 0.138 | Eastern | 0.520 | 0.036 | 0.004 | 0.410 | 0.023 | 0.008 | Eastern - Moderate |
| Austria | Neulengbach | 48.18333333 | 15.91 | Feb-07 | Gernot Hoch and Alex Schopf | 11-387-10 | 1 | 1 | 0.672 | 0.328 | Mixed | 0.007 | 0.189 | 0.010 | 0.745 | 0.011 | 0.037 | F2 - Moderate |
| Austria | Neulengbach | 48.18333333 | 15.91 | Feb-07 | Gernot Hoch and Alex Schopf | 11-387-11 | 1 | 1 | 0.946 | 0.054 | Eastern | 0.977 | 0.000 | 0.000 | 0.015 | 0.008 | 0.000 | Eastern |
| Austria | Neulengbach | 48.18333333 | 15.91 | Feb-07 | Gernot Hoch and Alex Schopf | 11-387-12 | 1 | 1 | 0.652 | 0.348 | Mixed | 0.008 | 0.222 | 0.003 | 0.711 | 0.005 | 0.051 | F2 - Moderate |
| Austria | Neulengbach | 48.18333333 | 15.91 | Feb-07 | Gernot Hoch and Alex Schopf | 11-387-13 | 1 | 1 | 0.754 | 0.246 | Mixed | 0.130 | 0.001 | 0.002 | 0.827 | 0.038 | 0.002 | F2 |
| Austria | Neulengbach | 48.18333333 | 15.91 | Feb-07 | Gernot Hoch and Alex Schopf | 11-387-14 | 1 | 1 | 0.583 | 0.417 | Mixed | 0.012 | 0.206 | 0.008 | 0.729 | 0.012 | 0.032 | F2 - Moderate |
| Austria | Neulengbach | 48.18333333 | 15.91 | Feb-07 | Gernot Hoch and Alex Schopf | 11-387-15 | 1 | 1 | 0.558 | 0.442 | Mixed | 0.000 | 0.775 | 0.004 | 0.187 | 0.001 | 0.033 | Western - Moderate |
| Austria | Neulengbach | 48.18333333 | 15.91 | Feb-07 | Gernot Hoch and Alex Schopf | 11-387-16 | 1 | 1 | 0.694 | 0.306 | Mixed | 0.007 | 0.582 | 0.005 | 0.373 | 0.006 | 0.028 | Western - Moderate |
| Austria | Neulengbach | 48.18333333 | 15.91 | Feb-07 | Gernot Hoch and Alex Schopf | 11-387-18 | 1 | 1 | 0.428 | 0.572 | Mixed | 0.000 | 0.954 | 0.000 | 0.037 | 0.000 | 0.009 | Western |
| Austria | Neulengbach | 48.18333333 | 15.91 | Feb-07 | Gernot Hoch and Alex Schopf | 11-387-19 | 1 | 1 | 0.747 | 0.253 | Mixed | 0.114 | 0.079 | 0.005 | 0.764 | 0.016 | 0.021 | F2 - Moderate |
| Austria | Neulengbach | 48.18333333 | 15.91 | Feb-07 | Gernot Hoch and Alex Schopf | 11-387-20 | 1 | 1 | 0.589 | 0.411 | Mixed | 0.000 | 0.017 | 0.009 | 0.912 | 0.005 | 0.057 | F2 |
| Austria | Neulengbach | 48.18333333 | 15.91 | Feb-07 | Gernot Hoch and Alex Schopf | 11-387-21 | 1 | 1 | 0.465 | 0.535 | Mixed | 0.001 | 0.847 | 0.001 | 0.130 | 0.001 | 0.020 | Western |
| Austria | Neulengbach | 48.18333333 | 15.91 | Feb-07 | Gernot Hoch and Alex Schopf | 11-387-22 | 1 | 1 | 0.909 | 0.091 | Eastern | 0.732 | 0.003 | 0.004 | 0.231 | 0.028 | 0.002 | Eastern - Moderate |
| Austria | Neulengbach | 48.18333333 | 15.91 | Feb-07 | Gernot Hoch and Alex Schopf | 11-387-23 | 1 | 1 | 0.623 | 0.377 | Mixed | 0.000 | 0.365 | 0.003 | 0.583 | 0.002 | 0.047 | F2 - Moderate |
| Austria | Neulengbach | 48.18333333 | 15.91 | Feb-07 | Gernot Hoch and Alex Schopf | 11-387-24 | 1 | 1 | 0.858 | 0.142 | Eastern | 0.532 | 0.002 | 0.005 | 0.428 | 0.029 | 0.004 | Eastern - Moderate |
| Austria | Neulengbach | 48.18333333 | 15.91 | Feb-07 | Gernot Hoch and Alex Schopf | 11-387-25 | 1 | 1 | 0.969 | 0.031 | Eastern | 0.996 | 0.000 | 0.000 | 0.002 | 0.002 | 0.000 | Eastern |
| Austria | Neulengbach | 48.18333333 | 15.91 | Feb-07 | Gernot Hoch and Alex Schopf | 11-387-26 | 1 | 1 | 0.839 | 0.161 | Eastern | 0.470 | 0.025 | 0.005 | 0.464 | 0.029 | 0.007 | Unassigned |
| Austria | Neulengbach | 48.18333333 | 15.91 | Feb-07 | Gernot Hoch and Alex Schopf | 11-387-27 | 1 | 1 | 0.571 | 0.429 | Mixed | 0.014 | 0.084 | 0.015 | 0.826 | 0.015 | 0.046 | F2 |
| Austria | Neulengbach | 48.18333333 | 15.91 | Feb-07 | Gernot Hoch and Alex Schopf | 11-387-28 | 1 | 1 | 0.884 | 0.116 | Eastern | 0.642 | 0.001 | 0.008 | 0.317 | 0.029 | 0.004 | Eastern - Moderate |
| Austria | Neulengbach | 48.18333333 | 15.91 | Feb-07 | Gernot Hoch and Alex Schopf | 11-387-29 | 1 | 1 | 0.57 | 0.43 | Mixed | 0.015 | 0.387 | 0.005 | 0.549 | 0.006 | 0.039 | F2 - Moderate |
| Austria | Neulengbach | 48.18333333 | 15.91 | Feb-07 | Gernot Hoch and Alex Schopf | 11-387-30 | 1 | 1 | 0.677 | 0.323 | Mixed | 0.003 | 0.490 | 0.035 | 0.408 | 0.008 | 0.056 | Unassigned |
| Austria | Neulengbach | 48.18333333 | 15.91 | Feb-07 | Gernot Hoch and Alex Schopf | 11-387-31 | 1 | 1 | 0.91 | 0.09 | Eastern | 0.770 | 0.009 | 0.001 | 0.202 | 0.016 | 0.002 | Eastern - Moderate |
| Austria | Neulengbach | 48.18333333 | 15.91 | Feb-07 | Gernot Hoch and Alex Schopf | 11-387-32 | 1 | 1 | 0.487 | 0.513 | Mixed | 0.000 | 0.794 | 0.003 | 0.169 | 0.001 | 0.033 | Western - Moderate |
| Austria | Neulengbach | 48.18333333 | 15.91 | Feb-07 | Gernot Hoch and Alex Schopf | 11-387-33 | 1 | 1 | 0.799 | 0.201 | Mixed | 0.045 | 0.425 | 0.004 | 0.489 | 0.007 | 0.030 | Unassigned |
| Austria | Neulengbach | 48.18333333 | 15.91 | Feb-07 | Gernot Hoch and Alex Schopf | 11-387-34 | 1 | 1 | 0.919 | 0.081 | Eastern | 0.898 | 0.000 | 0.001 | 0.087 | 0.013 | 0.001 | Eastern |
| Austria | Vienna | 48.23472222 | 16.34027778 | 11/27/06 | Alex Schopf | 06-213-01 | 1 | 2 | 0.891 | 0.109 | Eastern | 0.890 | 0.000 | 0.001 | 0.090 | 0.019 | 0.000 | Eastern |
| Austria | Vienna | 48.23472222 | 16.34027778 | 11/27/06 | Alex Schopf | 06-213-02 | 1 | 2 | 0.937 | 0.063 | Eastern | 0.913 | 0.000 | 0.000 | 0.073 | 0.014 | 0.000 | Eastern |
| Austria | Vienna | 48.23472222 | 16.34027778 | 11/27/06 | Alex Schopf | 06-213-03 | 1 | 2 | 0.655 | 0.345 | Mixed | 0.000 | 0.279 | 0.004 | 0.672 | 0.004 | 0.040 | F2 - Moderate |
| Austria | Vienna | 48.23472222 | 16.34027778 | 11/23/06 | Alex Schopf | 06-214-01 | 1 | 2 | 0.709 | 0.291 | Mixed | 0.250 | 0.049 | 0.006 | 0.655 | 0.025 | 0.014 | F2 - Moderate |
| Austria | Vienna | 48.23472222 | 16.34027778 | 11/23/06 | Alex Schopf | 06-214-02 | 1 | 2 | 0.953 | 0.047 | Eastern | 0.967 | 0.000 | 0.000 | 0.024 | 0.009 | 0.000 | Eastern |
| Austria | Vienna | 48.23472222 | 16.34027778 | 11/23/06 | Alex Schopf | 06-214-03 | 1 | 2 | 0.938 | 0.062 | Eastern | 0.960 | 0.000 | 0.000 | 0.031 | 0.009 | 0.000 | Eastern |
| Austria | Vienna | 48.23472222 | 16.34027778 | 11/23/06 | Alex Schopf | 06-214-04 | 1 | 2 | 0.772 | 0.228 | Mixed | 0.085 | 0.430 | 0.011 | 0.431 | 0.013 | 0.029 | Unassigned |
| Austria | Vienna | 48.23472222 | 16.34027778 | 11/23/06 | Alex Schopf | 06-214-05 | 1 | 2 | 0.879 | 0.121 | Eastern | 0.531 | 0.008 | 0.008 | 0.409 | 0.039 | 0.005 | Eastern - Moderate |
| Austria | Vienna | 48.23472222 | 16.34027778 | 11/23/06 | Alex Schopf | 06-214-06 | 1 | 2 | 0.501 | 0.499 | Mixed | 0.000 | 0.766 | 0.001 | 0.198 | 0.000 | 0.033 | Western - Moderate |
| Austria | Vienna | 48.24166667 | 16.29166667 | 11/28/06 | Alex Schopf | 06-215-02 | 1 | 2 | 0.875 | 0.125 | Eastern | 0.760 | 0.000 | 0.007 | 0.186 | 0.046 | 0.001 | Eastern - Moderate |
| Austria | Vienna | 48.24166667 | 16.29166667 | 11/28/06 | Alex Schopf | 06-215-03 | 1 | 2 | 0.904 | 0.096 | Eastern | 0.750 | 0.002 | 0.003 | 0.218 | 0.025 | 0.002 | Eastern - Moderate |
| Austria | Vienna | 48.24166667 | 16.29166667 | 11/28/06 | Alex Schopf | 06-215-04 | 1 | 2 | 0.746 | 0.254 | Mixed | 0.035 | 0.083 | 0.001 | 0.855 | 0.007 | 0.018 | F2 |
| Austria | Vienna | 48.24166667 | 16.29166667 | 11/28/06 | Alex Schopf | 06-215-05 | 1 | 2 | 0.818 | 0.182 | Eastern | 0.274 | 0.192 | 0.006 | 0.492 | 0.016 | 0.020 | Unassigned |
| Austria | Vienna | 48.24166667 | 16.29166667 | 11/28/06 | Alex Schopf | 06-215-06 | 1 | 2 | 0.867 | 0.133 | Eastern | 0.737 | 0.003 | 0.003 | 0.224 | 0.031 | 0.002 | Eastern - Moderate |
| Austria | Vienna | 48.24166667 | 16.29166667 | 11/28/06 | Alex Schopf | 06-215-07 | 1 | 2 | 0.572 | 0.428 | Mixed | 0.001 | 0.116 | 0.004 | 0.821 | 0.004 | 0.055 | F2 |
| Austria | Vienna | 48.24166667 | 16.29166667 | 11/28/06 | Alex Schopf | 06-215-08 | 1 | 2 | 0.777 | 0.223 | Mixed | 0.224 | 0.001 | 0.001 | 0.741 | 0.031 | 0.002 | F2 - Moderate |
| Austria | Vienna | 48.24166667 | 16.29166667 | 11/28/06 | Alex Schopf | 06-215-09 | 1 | 2 | 0.956 | 0.044 | Eastern | 0.987 | 0.000 | 0.000 | 0.009 | 0.004 | 0.000 | Eastern |
| Austria | Vienna | 48.24166667 | 16.29166667 | 11/28/06 | Alex Schopf | 06-215-10 | 1 | 2 | 0.972 | 0.028 | Eastern | 0.998 | 0.000 | 0.000 | 0.001 | 0.001 | 0.000 | Eastern |
| Austria | Vienna | 48.24166667 | 16.29166667 | 11/28/06 | Alex Schopf | 06-216-01 | 1 | 2 | 0.809 | 0.191 | Eastern | 0.161 | 0.003 | 0.004 | 0.800 | 0.020 | 0.012 | F2 - Moderate |
| Austria | Vienna | 48.24166667 | 16.29166667 | 11/28/06 | Alex Schopf | 06-216-02 | 1 | 2 | 0.95 | 0.05 | Eastern | 0.965 | 0.000 | 0.000 | 0.027 | 0.008 | 0.000 | Eastern |
| Austria | Vienna | 48.24166667 | 16.29166667 | 11/28/06 | Alex Schopf | 06-216-03 | 1 | 2 | 0.934 | 0.066 | Eastern | 0.956 | 0.000 | 0.000 | 0.037 | 0.007 | 0.000 | Eastern |
| Austria | Vienna | 48.24166667 | 16.29166667 | 11/28/06 | Alex Schopf | 06-216-04 | 1 | 2 | 0.86 | 0.14 | Eastern | 0.287 | 0.090 | 0.006 | 0.580 | 0.020 | 0.017 | F2 - Moderate |
| Austria | Vienna | 48.24166667 | 16.29166667 | 11/28/06 | Alex Schopf | 06-216-05 | 1 | 2 | 0.787 | 0.213 | Mixed | 0.272 | 0.007 | 0.013 | 0.654 | 0.046 | 0.009 | F2 - Moderate |
| Austria | Vienna | 48.24166667 | 16.29166667 | 11/28/06 | Alex Schopf | 06-216-06 | 1 | 2 | 0.713 | 0.287 | Mixed | 0.043 | 0.142 | 0.010 | 0.757 | 0.016 | 0.032 | F2 - Moderate |
| Austria | Vienna | 48.24166667 | 16.29166667 | 11/28/06 | Alex Schopf | 06-216-08 | 1 | 2 | 0.927 | 0.073 | Eastern | 0.724 | 0.016 | 0.002 | 0.239 | 0.016 | 0.004 | Eastern - Moderate |
| Austria | Vienna | 48.24166667 | 16.29166667 | 11/28/06 | Alex Schopf | 06-216-09 | 1 | 2 | 0.852 | 0.148 | Eastern | 0.438 | 0.021 | 0.009 | 0.487 | 0.036 | 0.009 | Unassigned |
| Austria | Vienna | 48.24166667 | 16.29166667 | 11/28/06 | Alex Schopf | 06-216-10 | 1 | 2 | 0.8 | 0.2 | Eastern | 0.295 | 0.009 | 0.009 | 0.634 | 0.046 | 0.008 | F2 - Moderate |
| Austria | Vienna | 48.23472222 | 16.34027778 | 11/13/13 | Gernot Hoch | 13-293-03 | 1 | 2 | 0.69 | 0.31 | Mixed | 0.029 | 0.087 | 0.019 | 0.811 | 0.025 | 0.029 | F2 |
| Austria | Vienna | 48.23472222 | 16.34027778 | 11/13/13 | Gernot Hoch | 13-293-04 | 1 | 2 | 0.887 | 0.113 | Eastern | 0.662 | 0.016 | 0.006 | 0.285 | 0.026 | 0.005 | Eastern - Moderate |
| Austria | Vienna | 48.23472222 | 16.34027778 | 11/13/13 | Gernot Hoch | 13-293-05 | 1 | 2 | 0.931 | 0.069 | Eastern | 0.905 | 0.000 | 0.001 | 0.076 | 0.017 | 0.000 | Eastern |
| Austria | Vienna | 48.23472222 | 16.34027778 | 11/13/13 | Gernot Hoch | 13-293-06 | 1 | 2 | 0.841 | 0.159 | Eastern | 0.213 | 0.107 | 0.006 | 0.635 | 0.019 | 0.019 | F2 - Moderate |
| Austria | Vienna | 48.23472222 | 16.34027778 | 11/13/13 | Gernot Hoch | 13-293-07 | 1 | 2 | 0.679 | 0.321 | Mixed | 0.018 | 0.522 | 0.003 | 0.420 | 0.004 | 0.033 | Western - Moderate |
| Austria | Vienna | 48.23472222 | 16.34027778 | 11/13/13 | Gernot Hoch | 13-293-08 | 1 | 2 | 0.802 | 0.198 | Eastern | 0.460 | 0.009 | 0.001 | 0.510 | 0.016 | 0.004 | F2 - Moderate |
| Austria | Vienna | 48.23472222 | 16.34027778 | 11/13/13 | Gernot Hoch | 13-293-09 | 1 | 2 | 0.775 | 0.225 | Mixed | 0.445 | 0.001 | 0.002 | 0.506 | 0.044 | 0.002 | F2 - Moderate |
| Austria | Vienna | 48.23472222 | 16.34027778 | 11/13/13 | Gernot Hoch | 13-293-10 | 1 | 2 | 0.494 | 0.506 | Mixed | 0.001 | 0.759 | 0.006 | 0.202 | 0.002 | 0.030 | Western - Moderate |
| Czech Republic | Prague | 50.08333333 | 14.41666667 | Dec-06 | Marek Turcáni | 06-197-01 | 2 | 3 | 0.288 | 0.712 | Mixed | 0.000 | 0.925 | 0.000 | 0.062 | 0.000 | 0.012 | Western |
| Czech Republic | Prague | 50.08333333 | 14.41666667 | Dec-06 | Marek Turcáni | 06-197-02 | 2 | 3 | 0.553 | 0.447 | Mixed | 0.004 | 0.340 | 0.011 | 0.589 | 0.006 | 0.050 | F2 - Moderate |
| Czech Republic | Prague | 50.08333333 | 14.41666667 | Dec-06 | Marek Turcáni | 06-197-03 | 2 | 3 | 0.931 | 0.069 | Eastern | 0.919 | 0.000 | 0.000 | 0.071 | 0.010 | 0.000 | Eastern |
| Czech Republic | Prague | 50.08333333 | 14.41666667 | Dec-06 | Marek Turcáni | 06-197-04 | 2 | 3 | 0.547 | 0.453 | Mixed | 0.001 | 0.748 | 0.002 | 0.216 | 0.001 | 0.033 | Western - Moderate |
| Czech Republic | Prague | 50.08333333 | 14.41666667 | Dec-06 | Marek Turcáni | 06-197-05 | 2 | 3 | 0.663 | 0.337 | Mixed | 0.002 | 0.206 | 0.002 | 0.754 | 0.004 | 0.032 | F2 - Moderate |
| Czech Republic | Prague | 50.08333333 | 14.41666667 | Dec-06 | Marek Turcáni | 06-197-06 | 2 | 3 | 0.575 | 0.425 | Mixed | 0.003 | 0.429 | 0.007 | 0.505 | 0.004 | 0.052 | F2 - Moderate |
| Czech Republic | Prague | 50.08333333 | 14.41666667 | Dec-06 | Marek Turcáni | 06-197-07 | 2 | 3 | 0.497 | 0.503 | Mixed | 0.000 | 0.645 | 0.005 | 0.297 | 0.001 | 0.052 | Western - Moderate |
| Czech Republic | Prague | 50.08333333 | 14.41666667 | Dec-06 | Marek Turcáni | 06-197-08 | 2 | 3 | 0.436 | 0.564 | Mixed | 0.000 | 0.702 | 0.004 | 0.255 | 0.001 | 0.037 | Western - Moderate |
| Czech Republic | Prague | 50.08333333 | 14.41666667 | Dec-06 | Marek Turcáni | 06-197-09 | 2 | 3 | 0.496 | 0.504 | Mixed | 0.000 | 0.839 | 0.004 | 0.119 | 0.001 | 0.037 | Western |
| Czech Republic | Prague | 50.08333333 | 14.41666667 | Dec-06 | Marek Turcáni | 06-197-10 | 2 | 3 | 0.832 | 0.168 | Eastern | 0.283 | 0.001 | 0.001 | 0.690 | 0.023 | 0.002 | F2 - Moderate |
| Czech Republic | Prague | 50.08333333 | 14.41666667 | Dec-06 | Marek Turcáni | 06-197-11 | 2 | 3 | 0.87 | 0.13 | Eastern | 0.698 | 0.002 | 0.001 | 0.274 | 0.024 | 0.001 | Eastern - Moderate |
| Czech Republic | Prague | 50.08333333 | 14.41666667 | Dec-06 | Marek Turcáni | 06-197-12 | 2 | 3 | 0.855 | 0.145 | Eastern | 0.343 | 0.162 | 0.009 | 0.444 | 0.024 | 0.017 | Unassigned |
| Czech Republic | Prague | 50.08333333 | 14.41666667 | Dec-06 | Marek Turcáni | 06-197-13 | 2 | 3 | 0.835 | 0.165 | Eastern | 0.593 | 0.000 | 0.001 | 0.362 | 0.044 | 0.000 | Eastern - Moderate |
| Czech Republic | Prague | 50.08333333 | 14.41666667 | Dec-06 | Marek Turcáni | 06-197-14 | 2 | 3 | 0.146 | 0.854 | Western | 0.000 | 0.986 | 0.000 | 0.008 | 0.000 | 0.005 | Western |
| Czech Republic | Prague | 50.08333333 | 14.41666667 | Dec-06 | Marek Turcáni | 06-197-15 | 2 | 3 | 0.928 | 0.072 | Eastern | 0.893 | 0.000 | 0.000 | 0.091 | 0.016 | 0.000 | Eastern |
| Czech Republic | Prague | 50.08333333 | 14.41666667 | Dec-06 | Marek Turcáni | 06-197-16 | 2 | 3 | 0.794 | 0.206 | Mixed | 0.283 | 0.059 | 0.004 | 0.619 | 0.021 | 0.013 | F2 - Moderate |
| Czech Republic | Prague | 50.08333333 | 14.41666667 | Dec-06 | Marek Turcáni | 06-197-17 | 2 | 3 | 0.199 | 0.801 | Western | 0.000 | 0.983 | 0.000 | 0.010 | 0.000 | 0.007 | Western |
| Czech Republic | Prague | 50.08333333 | 14.41666667 | Dec-06 | Marek Turcáni | 06-197-18 | 2 | 3 | 0.764 | 0.236 | Mixed | 0.479 | 0.001 | 0.002 | 0.487 | 0.028 | 0.003 | Unassigned |
| Czech Republic | Prague | 50.08333333 | 14.41666667 | Dec-06 | Marek Turcáni | 06-197-20 | 2 | 3 | 0.805 | 0.195 | Eastern | 0.405 | 0.017 | 0.002 | 0.545 | 0.025 | 0.006 | F2 - Moderate |
| England | Farnham | 51.17825 | -0.857 | 12/10/06 | Christine Tilbury | 06-202-01 | 3 | 4 | 0.052 | 0.948 | Western | 0.000 | 0.999 | 0.000 | 0.000 | 0.000 | 0.001 | Western |
| England | Farnham | 51.17825 | -0.857 | 12/10/06 | Christine Tilbury | 06-202-02 | 3 | 4 | 0.327 | 0.673 | Mixed | 0.000 | 0.939 | 0.000 | 0.045 | 0.000 | 0.016 | Western |
| England | Farnham | 51.17825 | -0.857 | 12/10/06 | Christine Tilbury | 06-202-03 | 3 | 4 | 0.046 | 0.954 | Western | 0.000 | 1.000 | 0.000 | 0.000 | 0.000 | 0.000 | Western |
| England | Farnham | 51.17825 | -0.857 | 12/10/06 | Christine Tilbury | 06-202-04 | 3 | 4 | 0.038 | 0.962 | Western | 0.000 | 1.000 | 0.000 | 0.000 | 0.000 | 0.000 | Western |
| England | Farnham | 51.17825 | -0.857 | 12/10/06 | Christine Tilbury | 06-202-05 | 3 | 4 | 0.049 | 0.951 | Western | 0.000 | 0.998 | 0.000 | 0.000 | 0.000 | 0.001 | Western |
| England | Farnham | 51.17825 | -0.857 | 12/10/06 | Christine Tilbury | 06-202-06 | 3 | 4 | 0.028 | 0.972 | Western | 0.000 | 1.000 | 0.000 | 0.000 | 0.000 | 0.000 | Western |
| England | Farnham | 51.17825 | -0.857 | 12/10/06 | Christine Tilbury | 06-202-07 | 3 | 4 | 0.025 | 0.975 | Western | 0.000 | 1.000 | 0.000 | 0.000 | 0.000 | 0.000 | Western |
| England | Farnham | 51.17825 | -0.857 | 12/10/06 | Christine Tilbury | 06-202-08 | 3 | 4 | 0.09 | 0.91 | Western | 0.000 | 0.998 | 0.000 | 0.000 | 0.000 | 0.001 | Western |
| England | Farnham | 51.17825 | -0.857 | 12/10/06 | Christine Tilbury | 06-202-09 | 3 | 4 | 0.194 | 0.806 | Western | 0.000 | 0.974 | 0.000 | 0.017 | 0.000 | 0.008 | Western |
| England | Farnham | 51.17825 | -0.857 | 12/10/06 | Christine Tilbury | 06-202-10 | 3 | 4 | 0.314 | 0.686 | Mixed | 0.000 | 0.389 | 0.000 | 0.581 | 0.000 | 0.030 | F2 - Moderate |
| England | Farnham | 51.17825 | -0.857 | 12/10/06 | Christine Tilbury | 06-202-12 | 3 | 4 | 0.298 | 0.702 | Mixed | 0.000 | 0.870 | 0.000 | 0.107 | 0.000 | 0.023 | Western |
| England | Farnham | 51.17825 | -0.857 | 12/10/06 | Christine Tilbury | 06-202-13 | 3 | 4 | 0.181 | 0.819 | Western | 0.000 | 0.971 | 0.000 | 0.014 | 0.000 | 0.015 | Western |
| England | Farnham | 51.17825 | -0.857 | 12/10/06 | Christine Tilbury | 06-202-14 | 3 | 4 | 0.143 | 0.857 | Western | 0.000 | 0.985 | 0.000 | 0.009 | 0.000 | 0.006 | Western |
| England | Farnham | 51.17825 | -0.857 | 12/10/06 | Christine Tilbury | 06-202-15 | 3 | 4 | 0.312 | 0.688 | Mixed | 0.000 | 0.960 | 0.000 | 0.026 | 0.000 | 0.013 | Western |
| England | Farnham | 51.18158333 | -0.853666667 | 12/10/06 | Christine Tilbury | 06-203-01 | 3 | 4 | 0.056 | 0.944 | Western | 0.000 | 0.999 | 0.000 | 0.000 | 0.000 | 0.001 | Western |
| England | Farnham | 51.18158333 | -0.853666667 | 12/10/06 | Christine Tilbury | 06-203-02 | 3 | 4 | 0.053 | 0.947 | Western | 0.000 | 0.999 | 0.000 | 0.000 | 0.000 | 0.001 | Western |
| England | Farnham | 51.18158333 | -0.853666667 | 12/10/06 | Christine Tilbury | 06-203-03 | 3 | 4 | 0.172 | 0.828 | Western | 0.000 | 0.991 | 0.000 | 0.005 | 0.000 | 0.005 | Western |
| England | Farnham | 51.18158333 | -0.853666667 | 12/10/06 | Christine Tilbury | 06-203-04 | 3 | 4 | 0.039 | 0.961 | Western | 0.000 | 0.999 | 0.000 | 0.000 | 0.000 | 0.001 | Western |
| England | Farnham | 51.18158333 | -0.853666667 | 12/10/06 | Christine Tilbury | 06-203-05 | 3 | 4 | 0.138 | 0.862 | Western | 0.000 | 0.988 | 0.000 | 0.005 | 0.000 | 0.007 | Western |
| England | Farnham | 51.18158333 | -0.853666667 | 12/10/06 | Christine Tilbury | 06-203-06 | 3 | 4 | 0.088 | 0.912 | Western | 0.000 | 0.997 | 0.000 | 0.000 | 0.000 | 0.002 | Western |
| England | Farnham | 51.18158333 | -0.853666667 | 12/10/06 | Christine Tilbury | 06-203-07 | 3 | 4 | 0.047 | 0.953 | Western | 0.000 | 1.000 | 0.000 | 0.000 | 0.000 | 0.000 | Western |
| England | Farnham | 51.18158333 | -0.853666667 | 12/10/06 | Christine Tilbury | 06-203-08 | 3 | 4 | 0.058 | 0.942 | Western | 0.000 | 0.999 | 0.000 | 0.000 | 0.000 | 0.001 | Western |
| England | Farnham | 51.18158333 | -0.853666667 | 12/10/06 | Christine Tilbury | 06-203-09 | 3 | 4 | 0.053 | 0.947 | Western | 0.000 | 0.999 | 0.000 | 0.000 | 0.000 | 0.001 | Western |
| England | Farnham | 51.18158333 | -0.853666667 | 12/10/06 | Christine Tilbury | 06-203-10 | 3 | 4 | 0.049 | 0.951 | Western | 0.000 | 0.999 | 0.000 | 0.000 | 0.000 | 0.001 | Western |
| England | Farnham | 51.18158333 | -0.853666667 | 12/10/06 | Christine Tilbury | 06-203-11 | 3 | 4 | 0.063 | 0.937 | Western | 0.000 | 0.998 | 0.000 | 0.000 | 0.000 | 0.001 | Western |
| England | Farnham | 51.18158333 | -0.853666667 | 12/10/06 | Christine Tilbury | 06-203-12 | 3 | 4 | 0.115 | 0.885 | Western | 0.000 | 0.995 | 0.000 | 0.002 | 0.000 | 0.003 | Western |
| England | Farnham | 51.18158333 | -0.853666667 | 12/10/06 | Christine Tilbury | 06-203-13 | 3 | 4 | 0.031 | 0.969 | Western | 0.000 | 1.000 | 0.000 | 0.000 | 0.000 | 0.000 | Western |
| England | Farnham | 51.18158333 | -0.853666667 | 12/10/06 | Christine Tilbury | 06-203-14 | 3 | 4 | 0.138 | 0.862 | Western | 0.000 | 0.996 | 0.000 | 0.001 | 0.000 | 0.002 | Western |
| England | Farnham | 51.18158333 | -0.853666667 | 12/10/06 | Christine Tilbury | 06-203-15 | 3 | 4 | 0.153 | 0.847 | Western | 0.000 | 0.993 | 0.000 | 0.003 | 0.000 | 0.004 | Western |
| England | Farnham | 51.17866667 | -0.850472222 | 12/10/06 | Christine Tilbury | 06-204-01 | 3 | 4 | 0.052 | 0.948 | Western | 0.000 | 0.999 | 0.000 | 0.000 | 0.000 | 0.001 | Western |
| England | Farnham | 51.17866667 | -0.850472222 | 12/10/06 | Christine Tilbury | 06-204-02 | 3 | 4 | 0.032 | 0.968 | Western | 0.000 | 1.000 | 0.000 | 0.000 | 0.000 | 0.000 | Western |
| England | Farnham | 51.17866667 | -0.850472222 | 12/10/06 | Christine Tilbury | 06-204-03 | 3 | 4 | 0.222 | 0.778 | Mixed | 0.000 | 0.956 | 0.000 | 0.027 | 0.000 | 0.016 | Western |
| England | Farnham | 51.17866667 | -0.850472222 | 12/10/06 | Christine Tilbury | 06-204-04 | 3 | 4 | 0.096 | 0.904 | Western | 0.000 | 0.997 | 0.000 | 0.001 | 0.000 | 0.002 | Western |
| England | Farnham | 51.17866667 | -0.850472222 | 12/10/06 | Christine Tilbury | 06-204-05 | 3 | 4 | 0.221 | 0.779 | Mixed | 0.000 | 0.989 | 0.000 | 0.005 | 0.000 | 0.006 | Western |
| England | Farnham | 51.17866667 | -0.850472222 | 12/10/06 | Christine Tilbury | 06-204-06 | 3 | 4 | 0.086 | 0.914 | Western | 0.000 | 0.996 | 0.000 | 0.001 | 0.000 | 0.003 | Western |
| England | Farnham | 51.17866667 | -0.850472222 | 12/10/06 | Christine Tilbury | 06-204-07 | 3 | 4 | 0.191 | 0.809 | Western | 0.000 | 0.985 | 0.000 | 0.009 | 0.000 | 0.006 | Western |
| England | Farnham | 51.17866667 | -0.850472222 | 12/10/06 | Christine Tilbury | 06-204-08 | 3 | 4 | 0.33 | 0.67 | Mixed | 0.000 | 0.957 | 0.000 | 0.029 | 0.000 | 0.014 | Western |
| England | Farnham | 51.17866667 | -0.850472222 | 12/10/06 | Christine Tilbury | 06-204-09 | 3 | 4 | 0.095 | 0.905 | Western | 0.000 | 0.999 | 0.000 | 0.000 | 0.000 | 0.001 | Western |
| England | Farnham | 51.17866667 | -0.850472222 | 12/10/06 | Christine Tilbury | 06-204-10 | 3 | 4 | 0.05 | 0.95 | Western | 0.000 | 0.999 | 0.000 | 0.000 | 0.000 | 0.001 | Western |
| England | Farnham | 51.17866667 | -0.850472222 | 12/10/06 | Christine Tilbury | 06-204-11 | 3 | 4 | 0.37 | 0.63 | Mixed | 0.000 | 0.733 | 0.001 | 0.231 | 0.001 | 0.033 | Western - Moderate |
| England | Farnham | 51.17866667 | -0.850472222 | 12/10/06 | Christine Tilbury | 06-204-12 | 3 | 4 | 0.175 | 0.825 | Western | 0.000 | 0.994 | 0.000 | 0.003 | 0.000 | 0.003 | Western |
| England | Farnham | 51.17866667 | -0.850472222 | 12/10/06 | Christine Tilbury | 06-204-13 | 3 | 4 | 0.05 | 0.95 | Western | 0.000 | 1.000 | 0.000 | 0.000 | 0.000 | 0.000 | Western |
| England | Farnham | 51.17866667 | -0.850472222 | 12/10/06 | Christine Tilbury | 06-204-14 | 3 | 4 | 0.054 | 0.946 | Western | 0.000 | 0.999 | 0.000 | 0.000 | 0.000 | 0.001 | Western |
| England | Farnham | 51.17866667 | -0.850472222 | 12/10/06 | Christine Tilbury | 06-204-15 | 3 | 4 | 0.158 | 0.842 | Western | 0.000 | 0.987 | 0.000 | 0.005 | 0.000 | 0.008 | Western |
| England | Farnham | 51.17825 | -0.857 |  |  | 14-025-01 | 3 | 4 | 0.318 | 0.682 | Mixed | 0.000 | 0.470 | 0.001 | 0.421 | 0.000 | 0.107 | Unassigned |
| England | Farnham | 51.17825 | -0.857 |  |  | 14-025-02 | 3 | 4 | 0.039 | 0.961 | Western | 0.000 | 1.000 | 0.000 | 0.000 | 0.000 | 0.000 | Western |
| England | Farnham | 51.17825 | -0.857 |  |  | 14-025-04 | 3 | 4 | 0.099 | 0.901 | Western | 0.000 | 0.997 | 0.000 | 0.000 | 0.000 | 0.002 | Western |
| England | Farnham | 51.17825 | -0.857 |  |  | 14-025-05 | 3 | 4 | 0.034 | 0.966 | Western | 0.000 | 1.000 | 0.000 | 0.000 | 0.000 | 0.000 | Western |
| France | Bédarieux | 43.5745 | 2.876 | 1/1/07 | F. Hérard | 325 | 4 | 5 | 0.356 | 0.644 | Mixed | 0.000 | 0.481 | 0.001 | 0.443 | 0.000 | 0.074 | Unassigned |
| France | Bédarieux | 43.5745 | 2.876 | 1/1/07 | F. Hérard | 326 | 4 | 5 | 0.302 | 0.698 | Mixed | 0.000 | 0.731 | 0.001 | 0.172 | 0.000 | 0.097 | Western - Moderate |
| France | Bédarieux | 43.5745 | 2.876 | 1/1/07 | F. Hérard | 327 | 4 | 5 | 0.291 | 0.709 | Mixed | 0.000 | 0.961 | 0.000 | 0.025 | 0.000 | 0.014 | Western |
| France | Bédarieux | 43.5745 | 2.876 | 1/1/07 | F. Hérard | 329 | 4 | 5 | 0.402 | 0.598 | Mixed | 0.000 | 0.874 | 0.001 | 0.098 | 0.000 | 0.027 | Western |
| France | Bédarieux | 43.5745 | 2.876 | 1/1/07 | F. Hérard | 06-196-01 | 4 | 5 | 0.264 | 0.736 | Mixed | 0.000 | 0.966 | 0.000 | 0.026 | 0.000 | 0.008 | Western |
| France | Bédarieux | 43.5745 | 2.876 | 1/1/07 | F. Hérard | 06-196-02 | 4 | 5 | 0.18 | 0.82 | Western | 0.000 | 0.935 | 0.000 | 0.028 | 0.000 | 0.037 | Western |
| France | Bédarieux | 43.5745 | 2.876 | 1/1/07 | F. Hérard | 06-196-03 | 4 | 5 | 0.059 | 0.941 | Western | 0.000 | 0.998 | 0.000 | 0.000 | 0.000 | 0.002 | Western |
| France | Bédarieux | 43.5745 | 2.876 | 1/1/07 | F. Hérard | 06-196-04 | 4 | 5 | 0.098 | 0.902 | Western | 0.000 | 0.997 | 0.000 | 0.001 | 0.000 | 0.002 | Western |
| France | Bédarieux | 43.5745 | 2.876 | 1/1/07 | F. Hérard | 06-196-05 | 4 | 5 | 0.045 | 0.955 | Western | 0.000 | 0.999 | 0.000 | 0.000 | 0.000 | 0.001 | Western |
| France | Bédarieux | 43.5745 | 2.876 | 1/1/07 | F. Hérard | 06-196-08 | 4 | 5 | 0.083 | 0.917 | Western | 0.000 | 0.997 | 0.000 | 0.001 | 0.000 | 0.002 | Western |
| France | Bédarieux | 43.5745 | 2.876 | 1/1/07 | F. Hérard | 06-196-09 | 4 | 5 | 0.037 | 0.963 | Western | 0.000 | 1.000 | 0.000 | 0.000 | 0.000 | 0.000 | Western |
| France | Bédarieux | 43.5745 | 2.876 | 1/1/07 | F. Hérard | 06-196-10 | 4 | 5 | 0.084 | 0.916 | Western | 0.000 | 0.996 | 0.000 | 0.002 | 0.000 | 0.003 | Western |
| France | Bédarieux | 43.5745 | 2.876 | 1/1/07 | F. Hérard | 06-196-11 | 4 | 5 | 0.07 | 0.93 | Western | 0.000 | 0.999 | 0.000 | 0.000 | 0.000 | 0.001 | Western |
| France | Bédarieux | 43.5745 | 2.876 | 1/1/07 | F. Hérard | 06-196-12 | 4 | 5 | 0.036 | 0.964 | Western | 0.000 | 1.000 | 0.000 | 0.000 | 0.000 | 0.000 | Western |
| France | Bédarieux | 43.5745 | 2.876 | 1/1/07 | F. Hérard | 06-196-13 | 4 | 5 | 0.268 | 0.732 | Mixed | 0.000 | 0.977 | 0.000 | 0.013 | 0.000 | 0.010 | Western |
| France | Bédarieux | 43.5745 | 2.876 | 1/1/07 | F. Hérard | 06-196-14 | 4 | 5 | 0.171 | 0.829 | Western | 0.000 | 0.982 | 0.000 | 0.010 | 0.000 | 0.008 | Western |
| France | Haguenau | 48.86 | 7.647666667 | 12/21/06 | F. Hérard | 268 | 4 | 6 | 0.176 | 0.824 | Western | 0.000 | 0.982 | 0.000 | 0.009 | 0.000 | 0.009 | Western |
| France | Haguenau | 48.86 | 7.647666667 | 12/21/06 | F. Hérard | 269 | 4 | 6 | 0.611 | 0.389 | Mixed | 0.003 | 0.268 | 0.024 | 0.639 | 0.011 | 0.055 | F2 - Moderate |
| France | Haguenau | 48.86 | 7.647666667 | 12/21/06 | F. Hérard | 271 | 4 | 6 | 0.592 | 0.408 | Mixed | 0.001 | 0.426 | 0.015 | 0.498 | 0.006 | 0.054 | F2 - Moderate |
| France | Haguenau | 48.86 | 7.647666667 | 12/21/06 | F. Hérard | 272 | 4 | 6 | 0.255 | 0.745 | Mixed | 0.000 | 0.986 | 0.000 | 0.008 | 0.000 | 0.006 | Western |
| France | Haguenau | 48.86 | 7.647666667 | 12/21/06 | F. Hérard | 06-195-01 | 4 | 6 | 0.085 | 0.915 | Western | 0.000 | 0.998 | 0.000 | 0.001 | 0.000 | 0.002 | Western |
| France | Haguenau | 48.86 | 7.647666667 | 12/21/06 | F. Hérard | 06-195-02 | 4 | 6 | 0.149 | 0.851 | Western | 0.000 | 0.992 | 0.000 | 0.003 | 0.000 | 0.004 | Western |
| France | Haguenau | 48.86 | 7.647666667 | 12/21/06 | F. Hérard | 06-195-04 | 4 | 6 | 0.201 | 0.799 | Mixed | 0.000 | 0.976 | 0.000 | 0.015 | 0.000 | 0.008 | Western |
| France | Haguenau | 48.86 | 7.647666667 | 12/21/06 | F. Hérard | 06-195-05 | 4 | 6 | 0.102 | 0.898 | Western | 0.000 | 0.993 | 0.000 | 0.003 | 0.000 | 0.003 | Western |
| France | Haguenau | 48.86 | 7.647666667 | 12/21/06 | F. Hérard | 06-195-06 | 4 | 6 | 0.096 | 0.904 | Western | 0.000 | 0.998 | 0.000 | 0.001 | 0.000 | 0.001 | Western |
| France | Haguenau | 48.86 | 7.647666667 | 12/21/06 | F. Hérard | 06-195-08 | 4 | 6 | 0.405 | 0.595 | Mixed | 0.000 | 0.945 | 0.000 | 0.038 | 0.000 | 0.017 | Western |
| France | Haguenau | 48.86 | 7.647666667 | 12/21/06 | F. Hérard | 06-195-09 | 4 | 6 | 0.552 | 0.448 | Mixed | 0.001 | 0.750 | 0.002 | 0.216 | 0.001 | 0.030 | Western - Moderate |
| France | Haguenau | 48.86 | 7.647666667 | 12/21/06 | F. Hérard | 06-195-10 | 4 | 6 | 0.241 | 0.759 | Mixed | 0.000 | 0.960 | 0.000 | 0.025 | 0.000 | 0.015 | Western |
| France | Haguenau | 48.86 | 7.647666667 | 12/21/06 | F. Hérard | 06-195-11 | 4 | 6 | 0.185 | 0.815 | Western | 0.000 | 0.986 | 0.000 | 0.008 | 0.000 | 0.006 | Western |
| France | Haguenau | 48.86 | 7.647666667 | 12/21/06 | F. Hérard | 06-195-12 | 4 | 6 | 0.225 | 0.775 | Mixed | 0.000 | 0.985 | 0.000 | 0.006 | 0.000 | 0.009 | Western |
| France | Haguenau | 48.86 | 7.647666667 | 12/21/06 | F. Hérard | 06-195-13 | 4 | 6 | 0.068 | 0.932 | Western | 0.000 | 0.999 | 0.000 | 0.000 | 0.000 | 0.001 | Western |
| France | Haguenau | 48.86 | 7.647666667 | 12/21/06 | F. Hérard | 06-195-15 | 4 | 6 | 0.792 | 0.208 | Mixed | 0.145 | 0.110 | 0.026 | 0.658 | 0.035 | 0.026 | F2 - Moderate |
| France | Haguenau | 48.86 | 7.647666667 | 12/21/06 | F. Hérard | 06-195-16 | 4 | 6 | 0.188 | 0.812 | Western | 0.000 | 0.980 | 0.000 | 0.012 | 0.000 | 0.008 | Western |
| France | Haguenau | 48.86 | 7.647666667 | 12/21/06 | F. Hérard | 06-195-17 | 4 | 6 | 0.762 | 0.238 | Mixed | 0.037 | 0.288 | 0.015 | 0.608 | 0.022 | 0.031 | F2 - Moderate |
| France | Haguenau | 48.86 | 7.647666667 | 12/21/06 | F. Hérard | 06-195-18 | 4 | 6 | 0.196 | 0.804 | Western | 0.000 | 0.989 | 0.000 | 0.004 | 0.000 | 0.007 | Western |
| France | Haguenau | 48.86 | 7.647666667 | 12/21/06 | F. Hérard | 06-195-19 | 4 | 6 | 0.189 | 0.811 | Western | 0.000 | 0.992 | 0.000 | 0.003 | 0.000 | 0.004 | Western |
| France | Haguenau | 48.86 | 7.647666667 | 12/21/06 | F. Hérard | 06-195-20 | 4 | 6 | 0.162 | 0.838 | Western | 0.000 | 0.993 | 0.000 | 0.003 | 0.000 | 0.004 | Western |
| France | Haguenau | 48.86 | 7.647666667 | 12/21/06 | F. Hérard | 06-195-21 | 4 | 6 | 0.331 | 0.669 | Mixed | 0.000 | 0.929 | 0.000 | 0.049 | 0.000 | 0.021 | Western |
| France | Haguenau | 48.86 | 7.647666667 | 12/21/06 | F. Hérard | 06-195-22 | 4 | 6 | 0.28 | 0.72 | Mixed | 0.000 | 0.961 | 0.000 | 0.020 | 0.000 | 0.019 | Western |
| France | Haguenau | 48.86 | 7.647666667 | 12/21/06 | F. Hérard | 06-195-23 | 4 | 6 | 0.166 | 0.834 | Western | 0.000 | 0.989 | 0.000 | 0.004 | 0.000 | 0.007 | Western |
| France | Haguenau | 48.86 | 7.647666667 | 12/21/06 | F. Hérard | 06-195-24 | 4 | 6 | 0.132 | 0.868 | Western | 0.000 | 0.991 | 0.000 | 0.004 | 0.000 | 0.005 | Western |
| France | Haguenau | 48.86 | 7.647666667 | 12/21/06 | F. Hérard | 06-195-25 | 4 | 6 | 0.149 | 0.851 | Western | 0.000 | 0.990 | 0.000 | 0.005 | 0.000 | 0.005 | Western |
| France | Haguenau | 48.86 | 7.647666667 | 12/21/06 | F. Hérard | 06-195-26 | 4 | 6 | 0.173 | 0.827 | Western | 0.000 | 0.991 | 0.000 | 0.003 | 0.000 | 0.006 | Western |
| France | Haguenau | 48.86 | 7.647666667 | 12/21/06 | F. Hérard | 06-195-27 | 4 | 6 | 0.178 | 0.822 | Western | 0.000 | 0.987 | 0.000 | 0.008 | 0.000 | 0.005 | Western |
| France | Haguenau | 48.86 | 7.647666667 | 12/21/06 | F. Hérard | 06-195-28 | 4 | 6 | 0.207 | 0.793 | Mixed | 0.000 | 0.987 | 0.000 | 0.006 | 0.000 | 0.006 | Western |
| France | Haguenau | 48.86 | 7.647666667 | 12/21/06 | F. Hérard | 06-195-29 | 4 | 6 | 0.601 | 0.399 | Mixed | 0.001 | 0.531 | 0.000 | 0.434 | 0.001 | 0.033 | Western - Moderate |
| France | Haguenau | 48.86 | 7.647666667 | 12/21/06 | F. Hérard | 06-195-30 | 4 | 6 | 0.131 | 0.869 | Western | 0.000 | 0.997 | 0.000 | 0.001 | 0.000 | 0.002 | Western |
| France | Marcillac | 44.4862 | 2.4670 | 12/24/13 | Alain Roques | 14-040-01 | 4 | 7 | 0.248 | 0.752 | Mixed | 0.000 | 0.973 | 0.000 | 0.012 | 0.000 | 0.015 | Western |
| France | Marcillac | 44.4862 | 2.4670 | 12/24/13 | Alain Roques | 14-040-02 | 4 | 7 | 0.111 | 0.889 | Western | 0.000 | 0.996 | 0.000 | 0.001 | 0.000 | 0.003 | Western |
| France | Marcillac | 44.4862 | 2.4670 | 12/24/13 | Alain Roques | 14-040-03 | 4 | 7 | 0.235 | 0.765 | Mixed | 0.000 | 0.978 | 0.000 | 0.013 | 0.000 | 0.009 | Western |
| France | Marcillac | 44.4862 | 2.4670 | 12/24/13 | Alain Roques | 14-040-04 | 4 | 7 | 0.342 | 0.658 | Mixed | 0.000 | 0.982 | 0.000 | 0.011 | 0.000 | 0.007 | Western |
| France | Marcillac | 44.4862 | 2.4670 | 12/24/13 | Alain Roques | 14-040-05 | 4 | 7 | 0.059 | 0.941 | Western | 0.000 | 0.999 | 0.000 | 0.000 | 0.000 | 0.001 | Western |
| France | Marcillac | 44.4862 | 2.4670 | 12/24/13 | Alain Roques | 14-040-06 | 4 | 7 | 0.134 | 0.866 | Western | 0.000 | 0.997 | 0.000 | 0.001 | 0.000 | 0.003 | Western |
| France | Marcillac | 44.4862 | 2.4670 | 12/24/13 | Alain Roques | 14-040-07 | 4 | 7 | 0.419 | 0.581 | Mixed | 0.000 | 0.840 | 0.000 | 0.125 | 0.000 | 0.034 | Western |
| France | Marcillac | 44.4862 | 2.4670 | 12/24/13 | Alain Roques | 14-040-08 | 4 | 7 | 0.278 | 0.722 | Mixed | 0.000 | 0.972 | 0.000 | 0.015 | 0.000 | 0.014 | Western |
| France | Marcillac | 44.4862 | 2.4670 | 12/24/13 | Alain Roques | 14-040-09 | 4 | 7 | 0.31 | 0.69 | Mixed | 0.000 | 0.925 | 0.000 | 0.051 | 0.000 | 0.023 | Western |
| France | Marcillac | 44.4862 | 2.4670 | 12/24/13 | Alain Roques | 14-040-10 | 4 | 7 | 0.222 | 0.778 | Mixed | 0.000 | 0.984 | 0.000 | 0.006 | 0.000 | 0.009 | Western |
| France | Marcillac | 44.4862 | 2.4670 | 12/24/13 | Alain Roques | 14-040-11 | 4 | 7 | 0.26 | 0.74 | Mixed | 0.000 | 0.991 | 0.000 | 0.004 | 0.000 | 0.004 | Western |
| France | Marcillac | 44.4862 | 2.4670 | 12/24/13 | Alain Roques | 14-040-12 | 4 | 7 | 0.324 | 0.676 | Mixed | 0.000 | 0.917 | 0.000 | 0.067 | 0.000 | 0.017 | Western |
| France | Marcillac | 44.4862 | 2.4670 | 12/24/13 | Alain Roques | 14-040-13 | 4 | 7 | 0.22 | 0.78 | Mixed | 0.000 | 0.983 | 0.000 | 0.008 | 0.000 | 0.009 | Western |
| France | Marcillac | 44.4862 | 2.4670 | 12/24/13 | Alain Roques | 14-040-14 | 4 | 7 | 0.273 | 0.727 | Mixed | 0.000 | 0.986 | 0.000 | 0.007 | 0.000 | 0.008 | Western |
| France | Marcillac | 44.4862 | 2.4670 | 12/24/13 | Alain Roques | 14-040-15 | 4 | 7 | 0.324 | 0.676 | Mixed | 0.000 | 0.956 | 0.001 | 0.024 | 0.000 | 0.019 | Western |
| France | Marcillac | 44.4862 | 2.4670 | 12/24/13 | Alain Roques | 14-040-16 | 4 | 7 | 0.245 | 0.755 | Mixed | 0.000 | 0.958 | 0.000 | 0.023 | 0.000 | 0.019 | Western |
| France | Marcillac | 44.4862 | 2.4670 | 12/24/13 | Alain Roques | 14-040-17 | 4 | 7 | 0.173 | 0.827 | Western | 0.000 | 0.988 | 0.000 | 0.006 | 0.000 | 0.005 | Western |
| France | Marcillac | 44.4862 | 2.4670 | 12/24/13 | Alain Roques | 14-040-19 | 4 | 7 | 0.088 | 0.912 | Western | 0.000 | 0.996 | 0.000 | 0.001 | 0.000 | 0.003 | Western |
| France | Marcillac | 44.4862 | 2.4670 | 12/24/13 | Alain Roques | 14-040-20 | 4 | 7 | 0.053 | 0.947 | Western | 0.000 | 0.999 | 0.000 | 0.000 | 0.000 | 0.001 | Western |
| France | Marcillac | 44.4862 | 2.4670 | 12/24/13 | Alain Roques | 14-040-21 | 4 | 7 | 0.489 | 0.511 | Mixed | 0.000 | 0.781 | 0.004 | 0.179 | 0.001 | 0.036 | Western - Moderate |
| France | Marcillac | 44.4862 | 2.4670 | 12/24/13 | Alain Roques | 14-040-22 | 4 | 7 | 0.084 | 0.916 | Western | 0.000 | 0.998 | 0.000 | 0.001 | 0.000 | 0.002 | Western |
| France | Marcillac | 44.4862 | 2.4670 | 12/24/13 | Alain Roques | 14-040-23 | 4 | 7 | 0.278 | 0.722 | Mixed | 0.000 | 0.955 | 0.000 | 0.030 | 0.000 | 0.015 | Western |
| France | Marcillac | 44.4862 | 2.4670 | 12/24/13 | Alain Roques | 14-040-24 | 4 | 7 | 0.378 | 0.622 | Mixed | 0.000 | 0.818 | 0.000 | 0.148 | 0.000 | 0.034 | Western |
| France | Marcillac | 44.4862 | 2.4670 | 12/24/13 | Alain Roques | 14-040-25 | 4 | 7 | 0.141 | 0.859 | Western | 0.000 | 0.997 | 0.000 | 0.001 | 0.000 | 0.002 | Western |
| France | Marcillac | 44.4862 | 2.4670 | 12/24/13 | Alain Roques | 14-040-26 | 4 | 7 | 0.059 | 0.941 | Western | 0.000 | 0.999 | 0.000 | 0.000 | 0.000 | 0.001 | Western |
| France | Marcillac | 44.4862 | 2.4670 | 12/24/13 | Alain Roques | 14-040-27 | 4 | 7 | 0.155 | 0.845 | Western | 0.000 | 0.992 | 0.000 | 0.003 | 0.000 | 0.005 | Western |
| France | Marcillac | 44.4862 | 2.4670 | 12/24/13 | Alain Roques | 14-040-28 | 4 | 7 | 0.196 | 0.804 | Western | 0.000 | 0.994 | 0.000 | 0.001 | 0.000 | 0.004 | Western |
| France | Marcillac | 44.4862 | 2.4670 | 12/24/13 | Alain Roques | 14-040-29 | 4 | 7 | 0.114 | 0.886 | Western | 0.000 | 0.989 | 0.000 | 0.004 | 0.000 | 0.006 | Western |
| France | Marcillac | 44.4862 | 2.4670 | 12/24/13 | Alain Roques | 14-040-30 | 4 | 7 | 0.254 | 0.746 | Mixed | 0.000 | 0.982 | 0.000 | 0.012 | 0.000 | 0.006 | Western |
| France | Montpellier | 43.748 | 3.768833333 | 12/26/06 | F. Hérard | 264 | 4 |  | 0.261 | 0.739 | Mixed | 0.000 | 0.979 | 0.000 | 0.013 | 0.000 | 0.008 | Western |
| France | Montpellier | 43.748 | 3.768833333 | 12/26/06 | F. Hérard | 265 | 4 |  | 0.133 | 0.867 | Western | 0.000 | 0.996 | 0.000 | 0.002 | 0.000 | 0.003 | Western |
| France | Montpellier | 43.748 | 3.768833333 | 12/26/06 | F. Hérard | 266 | 4 |  | 0.345 | 0.655 | Mixed | 0.000 | 0.955 | 0.001 | 0.030 | 0.000 | 0.014 | Western |
| France | Montpellier | 43.748 | 3.768833333 | 12/26/06 | F. Hérard | 06-193-01 | 4 |  | 0.033 | 0.967 | Western | 0.000 | 1.000 | 0.000 | 0.000 | 0.000 | 0.000 | Western |
| France | Onet L'eglise | 44.4382 | 2.5521 | 12/24/13 | Alain Roques | 14-039-01 | 4 | 8 | 0.103 | 0.897 | Western | 0.000 | 0.993 | 0.000 | 0.003 | 0.000 | 0.004 | Western |
| France | Onet L'eglise | 44.4382 | 2.5521 | 12/24/13 | Alain Roques | 14-039-02 | 4 | 8 | 0.179 | 0.821 | Western | 0.000 | 0.983 | 0.000 | 0.009 | 0.000 | 0.008 | Western |
| France | Onet L'eglise | 44.4382 | 2.5521 | 12/24/13 | Alain Roques | 14-039-03 | 4 | 8 | 0.13 | 0.87 | Western | 0.000 | 0.994 | 0.000 | 0.002 | 0.000 | 0.004 | Western |
| France | Onet L'eglise | 44.4382 | 2.5521 | 12/24/13 | Alain Roques | 14-039-04 | 4 | 8 | 0.038 | 0.962 | Western | 0.000 | 0.999 | 0.000 | 0.000 | 0.000 | 0.000 | Western |
| France | Onet L'eglise | 44.4382 | 2.5521 | 12/24/13 | Alain Roques | 14-039-05 | 4 | 8 | 0.063 | 0.937 | Western | 0.000 | 0.999 | 0.000 | 0.000 | 0.000 | 0.001 | Western |
| France | Onet L'eglise | 44.4382 | 2.5521 | 12/24/13 | Alain Roques | 14-039-06 | 4 | 8 | 0.125 | 0.875 | Western | 0.000 | 0.996 | 0.000 | 0.001 | 0.000 | 0.003 | Western |
| France | Onet L'eglise | 44.4382 | 2.5521 | 12/24/13 | Alain Roques | 14-039-07 | 4 | 8 | 0.334 | 0.666 | Mixed | 0.000 | 0.931 | 0.001 | 0.050 | 0.000 | 0.018 | Western |
| France | Onet L'eglise | 44.4382 | 2.5521 | 12/24/13 | Alain Roques | 14-039-08 | 4 | 8 | 0.083 | 0.917 | Western | 0.000 | 0.998 | 0.000 | 0.000 | 0.000 | 0.002 | Western |
| France | Onet L'eglise | 44.4382 | 2.5521 | 12/24/13 | Alain Roques | 14-039-09 | 4 | 8 | 0.055 | 0.945 | Western | 0.000 | 0.998 | 0.000 | 0.000 | 0.000 | 0.001 | Western |
| France | Onet L'eglise | 44.4382 | 2.5521 | 12/24/13 | Alain Roques | 14-039-10 | 4 | 8 | 0.033 | 0.967 | Western | 0.000 | 1.000 | 0.000 | 0.000 | 0.000 | 0.000 | Western |
| France | Onet L'eglise | 44.4382 | 2.5521 | 12/24/13 | Alain Roques | 14-039-11 | 4 | 8 | 0.044 | 0.956 | Western | 0.000 | 0.999 | 0.000 | 0.000 | 0.000 | 0.001 | Western |
| France | Onet L'eglise | 44.4382 | 2.5521 | 12/24/13 | Alain Roques | 14-039-12 | 4 | 8 | 0.293 | 0.707 | Mixed | 0.000 | 0.894 | 0.000 | 0.083 | 0.000 | 0.023 | Western |
| France | Onet L'eglise | 44.4382 | 2.5521 | 12/24/13 | Alain Roques | 14-039-13 | 4 | 8 | 0.23 | 0.77 | Mixed | 0.000 | 0.979 | 0.000 | 0.014 | 0.000 | 0.007 | Western |
| France | Onet L'eglise | 44.4382 | 2.5521 | 12/24/13 | Alain Roques | 14-039-14 | 4 | 8 | 0.048 | 0.952 | Western | 0.000 | 0.999 | 0.000 | 0.000 | 0.000 | 0.001 | Western |
| France | Onet L'eglise | 44.4382 | 2.5521 | 12/24/13 | Alain Roques | 14-039-15 | 4 | 8 | 0.334 | 0.666 | Mixed | 0.000 | 0.528 | 0.005 | 0.366 | 0.001 | 0.100 | Western - Moderate |
| France | Onet L'eglise | 44.4382 | 2.5521 | 12/24/13 | Alain Roques | 14-039-16 | 4 | 8 | 0.077 | 0.923 | Western | 0.000 | 0.997 | 0.000 | 0.001 | 0.000 | 0.002 | Western |
| France | Onet L'eglise | 44.4382 | 2.5521 | 12/24/13 | Alain Roques | 14-039-17 | 4 | 8 | 0.037 | 0.963 | Western | 0.000 | 1.000 | 0.000 | 0.000 | 0.000 | 0.000 | Western |
| France | Onet L'eglise | 44.4382 | 2.5521 | 12/24/13 | Alain Roques | 14-039-18 | 4 | 8 | 0.039 | 0.961 | Western | 0.000 | 1.000 | 0.000 | 0.000 | 0.000 | 0.000 | Western |
| France | Onet L'eglise | 44.4382 | 2.5521 | 12/24/13 | Alain Roques | 14-039-19 | 4 | 8 | 0.069 | 0.931 | Western | 0.000 | 0.998 | 0.000 | 0.001 | 0.000 | 0.002 | Western |
| France | Onet L'eglise | 44.4382 | 2.5521 | 12/24/13 | Alain Roques | 14-039-20 | 4 | 8 | 0.058 | 0.942 | Western | 0.000 | 0.998 | 0.000 | 0.000 | 0.000 | 0.001 | Western |
| France | Orléans | 47.8284 | 1.1915 | 1/5/14 | Alain Roques | 14-033-02 | 4 |  | 0.086 | 0.914 | Western | 0.000 | 0.997 | 0.000 | 0.001 | 0.000 | 0.002 | Western |
| France | Rennes | 48.02366667 | -2.222833333 | 12/22/06 | F. Hérard | 258 | 4 | 9 | 0.042 | 0.958 | Western | 0.000 | 1.000 | 0.000 | 0.000 | 0.000 | 0.000 | Western |
| France | Rennes | 48.02366667 | -2.222833333 | 12/22/06 | F. Hérard | 259 | 4 | 9 | 0.051 | 0.949 | Western | 0.000 | 0.999 | 0.000 | 0.000 | 0.000 | 0.001 | Western |
| France | Rennes | 48.02366667 | -2.222833333 | 12/22/06 | F. Hérard | 260 | 4 | 9 | 0.232 | 0.768 | Mixed | 0.000 | 0.995 | 0.000 | 0.002 | 0.000 | 0.003 | Western |
| France | Rennes | 48.02366667 | -2.222833333 | 12/22/06 | F. Hérard | 261 | 4 | 9 | 0.221 | 0.779 | Mixed | 0.000 | 0.986 | 0.000 | 0.007 | 0.000 | 0.007 | Western |
| France | Rennes | 48.02366667 | -2.222833333 | 12/22/06 | F. Hérard | 262 | 4 | 9 | 0.292 | 0.708 | Mixed | 0.000 | 0.959 | 0.000 | 0.030 | 0.000 | 0.011 | Western |
| France | Rennes | 48.02366667 | -2.222833333 | 12/22/06 | F. Hérard | 06-194-13 | 4 | 9 | 0.056 | 0.944 | Western | 0.000 | 0.999 | 0.000 | 0.000 | 0.000 | 0.001 | Western |
| France | Rennes | 48.02366667 | -2.222833333 | 12/22/06 | F. Hérard | 06-194-14 | 4 | 9 | 0.19 | 0.81 | Western | 0.000 | 0.989 | 0.000 | 0.006 | 0.000 | 0.005 | Western |
| France | Rennes | 48.02366667 | -2.222833333 | 12/22/06 | F. Hérard | 06-194-15 | 4 | 9 | 0.256 | 0.744 | Mixed | 0.000 | 0.978 | 0.000 | 0.010 | 0.000 | 0.012 | Western |
| France | Rennes | 48.02366667 | -2.222833333 | 12/22/06 | F. Hérard | 06-194-16 | 4 | 9 | 0.144 | 0.856 | Western | 0.000 | 0.993 | 0.000 | 0.003 | 0.000 | 0.005 | Western |
| France | Rennes | 48.02366667 | -2.222833333 | 12/22/06 | F. Hérard | 06-194-17 | 4 | 9 | 0.359 | 0.641 | Mixed | 0.000 | 0.846 | 0.004 | 0.107 | 0.000 | 0.043 | Western |
| France | Rennes | 48.02366667 | -2.222833333 | 12/22/06 | F. Hérard | 06-194-18 | 4 | 9 | 0.125 | 0.875 | Western | 0.000 | 0.991 | 0.000 | 0.005 | 0.000 | 0.004 | Western |
| France | Rennes | 48.02366667 | -2.222833333 | 12/22/06 | F. Hérard | 06-194-19 | 4 | 9 | 0.196 | 0.804 | Western | 0.000 | 0.986 | 0.000 | 0.008 | 0.000 | 0.005 | Western |
| France | Rennes | 48.02366667 | -2.222833333 | 12/22/06 | F. Hérard | 06-194-20 | 4 | 9 | 0.067 | 0.933 | Western | 0.000 | 0.997 | 0.000 | 0.001 | 0.000 | 0.002 | Western |
| France | Rennes | 48.02366667 | -2.222833333 | 12/22/06 | F. Hérard | 06-194-21 | 4 | 9 | 0.271 | 0.729 | Mixed | 0.000 | 0.970 | 0.000 | 0.018 | 0.000 | 0.012 | Western |
| France | Rennes | 48.02366667 | -2.222833333 | 12/22/06 | F. Hérard | 06-194-22 | 4 | 9 | 0.34 | 0.66 | Mixed | 0.000 | 0.964 | 0.000 | 0.023 | 0.000 | 0.013 | Western |
| France | Rennes | 48.02366667 | -2.222833333 | 12/22/06 | F. Hérard | 06-194-23 | 4 | 9 | 0.185 | 0.815 | Western | 0.000 | 0.984 | 0.000 | 0.007 | 0.000 | 0.008 | Western |
| France | Rennes | 48.02366667 | -2.222833333 | 12/22/06 | F. Hérard | 06-194-24 | 4 | 9 | 0.237 | 0.763 | Mixed | 0.000 | 0.948 | 0.000 | 0.037 | 0.000 | 0.015 | Western |
| France | Rennes | 48.02366667 | -2.222833333 | 12/22/06 | F. Hérard | 06-194-25 | 4 | 9 | 0.094 | 0.906 | Western | 0.000 | 0.997 | 0.000 | 0.001 | 0.000 | 0.002 | Western |
| France | Rennes | 48.02366667 | -2.222833333 | 12/22/06 | F. Hérard | 06-194-26 | 4 | 9 | 0.247 | 0.753 | Mixed | 0.000 | 0.971 | 0.000 | 0.016 | 0.000 | 0.013 | Western |
| France | Rennes | 48.02366667 | -2.222833333 | 12/22/06 | F. Hérard | 06-194-27 | 4 | 9 | 0.1 | 0.9 | Western | 0.000 | 0.997 | 0.000 | 0.001 | 0.000 | 0.002 | Western |
| France | Rennes | 48.02366667 | -2.222833333 | 12/22/06 | F. Hérard | 06-194-28 | 4 | 9 | 0.131 | 0.869 | Western | 0.000 | 0.995 | 0.000 | 0.002 | 0.000 | 0.003 | Western |
| France | Rennes | 48.02366667 | -2.222833333 | 12/22/06 | F. Hérard | 06-194-29 | 4 | 9 | 0.251 | 0.749 | Mixed | 0.000 | 0.986 | 0.000 | 0.008 | 0.000 | 0.006 | Western |
| France | Rennes | 48.02366667 | -2.222833333 | 12/22/06 | F. Hérard | 06-194-30 | 4 | 9 | 0.155 | 0.845 | Western | 0.000 | 0.987 | 0.000 | 0.005 | 0.000 | 0.008 | Western |
| Georgia | Tbilisi | 41.720845 | 44.829894 |  | George Japoshvili | 15-177-01 | 5 | 10 | 0.903 | 0.097 | Eastern | 0.981 | 0.000 | 0.000 | 0.013 | 0.006 | 0.000 | Eastern |
| Georgia | Tbilisi | 41.720845 | 44.829894 |  | George Japoshvili | 15-177-02 | 5 | 10 | 0.939 | 0.061 | Eastern | 0.996 | 0.000 | 0.000 | 0.002 | 0.002 | 0.000 | Eastern |
| Georgia | Tbilisi | 41.720845 | 44.829894 |  | George Japoshvili | 15-177-03 | 5 | 10 | 0.964 | 0.036 | Eastern | 0.998 | 0.000 | 0.000 | 0.001 | 0.001 | 0.000 | Eastern |
| Georgia | Tbilisi | 41.720845 | 44.829894 |  | George Japoshvili | 15-177-04 | 5 | 10 | 0.967 | 0.033 | Eastern | 1.000 | 0.000 | 0.000 | 0.000 | 0.000 | 0.000 | Eastern |
| Georgia | Tbilisi | 41.720845 | 44.829894 |  | George Japoshvili | 15-177-05 | 5 | 10 | 0.909 | 0.091 | Eastern | 0.991 | 0.000 | 0.000 | 0.003 | 0.006 | 0.000 | Eastern |
| Georgia | Tbilisi | 41.720845 | 44.829894 |  | George Japoshvili | 15-177-06 | 5 | 10 | 0.961 | 0.039 | Eastern | 0.999 | 0.000 | 0.000 | 0.000 | 0.001 | 0.000 | Eastern |
| Georgia | Tbilisi | 41.720845 | 44.829894 |  | George Japoshvili | 15-177-07 | 5 | 10 | 0.91 | 0.09 | Eastern | 0.982 | 0.000 | 0.000 | 0.009 | 0.009 | 0.000 | Eastern |
| Georgia | Tbilisi | 41.720845 | 44.829894 |  | George Japoshvili | 15-177-08 | 5 | 10 | 0.886 | 0.114 | Eastern | 0.968 | 0.000 | 0.000 | 0.019 | 0.013 | 0.000 | Eastern |
| Georgia | Tbilisi | 41.720845 | 44.829894 |  | George Japoshvili | 15-177-09 | 5 | 10 | 0.941 | 0.059 | Eastern | 0.992 | 0.000 | 0.000 | 0.004 | 0.003 | 0.000 | Eastern |
| Georgia | Tbilisi | 41.720845 | 44.829894 |  | George Japoshvili | 15-177-10 | 5 | 10 | 0.937 | 0.063 | Eastern | 0.991 | 0.000 | 0.000 | 0.006 | 0.003 | 0.000 | Eastern |
| Georgia | Tbilisi | 41.720845 | 44.829894 |  | George Japoshvili | 15-177-11 | 5 | 10 | 0.969 | 0.031 | Eastern | 0.999 | 0.000 | 0.000 | 0.000 | 0.001 | 0.000 | Eastern |
| Georgia | Tbilisi | 41.720845 | 44.829894 |  | George Japoshvili | 15-177-12 | 5 | 10 | 0.973 | 0.027 | Eastern | 0.999 | 0.000 | 0.000 | 0.000 | 0.001 | 0.000 | Eastern |
| Georgia | Tbilisi | 41.720845 | 44.829894 |  | George Japoshvili | 15-177-13 | 5 | 10 | 0.95 | 0.05 | Eastern | 0.995 | 0.000 | 0.000 | 0.003 | 0.002 | 0.000 | Eastern |
| Georgia | Tbilisi | 41.720845 | 44.829894 |  | George Japoshvili | 15-177-14 | 5 | 10 | 0.885 | 0.115 | Eastern | 0.889 | 0.000 | 0.000 | 0.098 | 0.013 | 0.000 | Eastern |
| Georgia | Tbilisi | 41.720845 | 44.829894 |  | George Japoshvili | 15-177-15 | 5 | 10 | 0.918 | 0.082 | Eastern | 0.994 | 0.000 | 0.000 | 0.001 | 0.005 | 0.000 | Eastern |
| Georgia | Tbilisi | 41.720845 | 44.829894 |  | George Japoshvili | 15-177-16 | 5 | 10 | 0.899 | 0.101 | Eastern | 0.986 | 0.000 | 0.000 | 0.007 | 0.007 | 0.000 | Eastern |
| Georgia | Tbilisi | 41.720845 | 44.829894 |  | George Japoshvili | 15-177-17 | 5 | 10 | 0.958 | 0.042 | Eastern | 0.997 | 0.000 | 0.000 | 0.001 | 0.001 | 0.000 | Eastern |
| Georgia | Tbilisi | 41.720845 | 44.829894 |  | George Japoshvili | 15-177-19 | 5 | 10 | 0.962 | 0.038 | Eastern | 0.998 | 0.000 | 0.000 | 0.001 | 0.001 | 0.000 | Eastern |
| Georgia | Tbilisi | 41.720845 | 44.829894 |  | George Japoshvili | 15-177-20 | 5 | 10 | 0.97 | 0.03 | Eastern | 0.999 | 0.000 | 0.000 | 0.000 | 0.001 | 0.000 | Eastern |
| Georgia | Tbilisi | 41.720845 | 44.829894 |  | George Japoshvili | 15-177-21 | 5 | 10 | 0.858 | 0.142 | Eastern | 0.905 | 0.000 | 0.001 | 0.054 | 0.039 | 0.000 | Eastern |
| Georgia | Tbilisi | 41.720845 | 44.829894 |  | George Japoshvili | 15-177-22 | 5 | 10 | 0.974 | 0.026 | Eastern | 1.000 | 0.000 | 0.000 | 0.000 | 0.000 | 0.000 | Eastern |
| Georgia | Tbilisi | 41.720845 | 44.829894 |  | George Japoshvili | 15-177-23 | 5 | 10 | 0.922 | 0.078 | Eastern | 0.990 | 0.000 | 0.000 | 0.005 | 0.005 | 0.000 | Eastern |
| Georgia | Tbilisi | 41.720845 | 44.829894 |  | George Japoshvili | 15-177-24 | 5 | 10 | 0.961 | 0.039 | Eastern | 0.999 | 0.000 | 0.000 | 0.000 | 0.000 | 0.000 | Eastern |
| Georgia | Tbilisi | 41.720845 | 44.829894 |  | George Japoshvili | 15-177-25 | 5 | 10 | 0.967 | 0.033 | Eastern | 0.999 | 0.000 | 0.000 | 0.000 | 0.001 | 0.000 | Eastern |
| Georgia | Tbilisi | 41.720845 | 44.829894 |  | George Japoshvili | 15-177-26 | 5 | 10 | 0.974 | 0.026 | Eastern | 1.000 | 0.000 | 0.000 | 0.000 | 0.000 | 0.000 | Eastern |
| Georgia | Tbilisi | 41.720845 | 44.829894 |  | George Japoshvili | 15-177-27 | 5 | 10 | 0.935 | 0.065 | Eastern | 0.990 | 0.000 | 0.000 | 0.007 | 0.003 | 0.000 | Eastern |
| Georgia | Tbilisi | 41.720845 | 44.829894 |  | George Japoshvili | 15-177-28 | 5 | 10 | 0.913 | 0.087 | Eastern | 0.990 | 0.000 | 0.000 | 0.002 | 0.008 | 0.000 | Eastern |
| Germany | Bühren | 51.46581 | 9.6778 | 12/18/06 | Berg | 06-170-01 | 6 | 11 | 0.477 | 0.523 | Mixed | 0.000 | 0.786 | 0.007 | 0.169 | 0.001 | 0.037 | Western - Moderate |
| Germany | Bühren | 51.46581 | 9.6778 | 12/18/06 | Berg | 06-170-02 | 6 | 11 | 0.427 | 0.573 | Mixed | 0.000 | 0.900 | 0.000 | 0.079 | 0.000 | 0.021 | Western |
| Germany | Bühren | 51.46581 | 9.6778 | 12/18/06 | Berg | 06-170-03 | 6 | 11 | 0.519 | 0.481 | Mixed | 0.000 | 0.626 | 0.007 | 0.314 | 0.001 | 0.051 | Western - Moderate |
| Germany | Bühren | 51.46581 | 9.6778 | 12/18/06 | Berg | 06-170-04 | 6 | 11 | 0.382 | 0.618 | Mixed | 0.000 | 0.893 | 0.000 | 0.075 | 0.000 | 0.032 | Western |
| Germany | Bühren | 51.46581 | 9.6778 | 12/18/06 | Berg | 06-170-05 | 6 | 11 | 0.352 | 0.648 | Mixed | 0.000 | 0.949 | 0.000 | 0.035 | 0.000 | 0.016 | Western |
| Germany | Bühren | 51.46581 | 9.6778 | 12/18/06 | Berg | 06-170-06 | 6 | 11 | 0.26 | 0.74 | Mixed | 0.000 | 0.982 | 0.000 | 0.009 | 0.000 | 0.009 | Western |
| Germany | Bühren | 51.46581 | 9.6778 | 12/18/06 | Berg | 06-170-07 | 6 | 11 | 0.299 | 0.701 | Mixed | 0.000 | 0.955 | 0.000 | 0.032 | 0.000 | 0.013 | Western |
| Germany | Bühren | 51.46581 | 9.6778 | 12/18/06 | Berg | 06-170-08 | 6 | 11 | 0.482 | 0.518 | Mixed | 0.000 | 0.736 | 0.001 | 0.234 | 0.000 | 0.029 | Western - Moderate |
| Germany | Bühren | 51.46581 | 9.6778 | 12/18/06 | Berg | 06-170-09 | 6 | 11 | 0.309 | 0.691 | Mixed | 0.000 | 0.957 | 0.000 | 0.033 | 0.000 | 0.011 | Western |
| Germany | Bühren | 51.46581 | 9.6778 | 12/18/06 | Berg | 06-170-10 | 6 | 11 | 0.076 | 0.924 | Western | 0.000 | 0.998 | 0.000 | 0.001 | 0.000 | 0.002 | Western |
| Germany | Bühren | 51.46581 | 9.6778 | 12/18/06 | Berg | 06-170-11 | 6 | 11 | 0.134 | 0.866 | Western | 0.000 | 0.992 | 0.000 | 0.003 | 0.000 | 0.005 | Western |
| Germany | Bühren | 51.46581 | 9.6778 | 12/18/06 | Berg | 06-170-12 | 6 | 11 | 0.121 | 0.879 | Western | 0.000 | 0.998 | 0.000 | 0.000 | 0.000 | 0.001 | Western |
| Germany | Bühren | 51.46581 | 9.6778 | 12/18/06 | Berg | 06-170-13 | 6 | 11 | 0.307 | 0.693 | Mixed | 0.000 | 0.977 | 0.000 | 0.013 | 0.000 | 0.010 | Western |
| Germany | Bühren | 51.46581 | 9.6778 | 12/18/06 | Berg | 06-170-14 | 6 | 11 | 0.482 | 0.518 | Mixed | 0.001 | 0.614 | 0.000 | 0.361 | 0.001 | 0.023 | Western - Moderate |
| Germany | Bühren | 51.46581 | 9.6778 | 12/18/06 | Berg | 06-170-15 | 6 | 11 | 0.517 | 0.483 | Mixed | 0.000 | 0.613 | 0.003 | 0.335 | 0.001 | 0.048 | Western - Moderate |
| Germany | Bühren | 51.46581 | 9.6778 | 12/18/06 | Berg | 06-170-16 | 6 | 11 | 0.117 | 0.883 | Western | 0.000 | 0.997 | 0.000 | 0.001 | 0.000 | 0.002 | Western |
| Germany | Bühren | 51.46581 | 9.6778 | 12/18/06 | Berg | 06-170-17 | 6 | 11 | 0.708 | 0.292 | Mixed | 0.018 | 0.262 | 0.008 | 0.667 | 0.011 | 0.034 | F2 - Moderate |
| Germany | Bühren | 51.46581 | 9.6778 | 12/18/06 | Berg | 06-170-18 | 6 | 11 | 0.414 | 0.586 | Mixed | 0.000 | 0.952 | 0.000 | 0.035 | 0.000 | 0.012 | Western |
| Germany | Bühren | 51.46581 | 9.6778 | 12/18/06 | Berg | 06-170-19 | 6 | 11 | 0.578 | 0.422 | Mixed | 0.005 | 0.039 | 0.006 | 0.896 | 0.007 | 0.046 | F2 |
| Germany | Bühren | 51.46581 | 9.6778 | 12/18/06 | Berg | 06-170-20 | 6 | 11 | 0.361 | 0.639 | Mixed | 0.000 | 0.108 | 0.021 | 0.601 | 0.001 | 0.269 | F2 - Moderate |
| Germany | Bühren | 51.46581 | 9.6778 | 12/18/06 | Berg | 06-170-21 | 6 | 11 | 0.162 | 0.838 | Western | 0.000 | 0.990 | 0.000 | 0.004 | 0.000 | 0.005 | Western |
| Germany | Bühren | 51.46581 | 9.6778 | 12/18/06 | Berg | 06-170-22 | 6 | 11 | 0.092 | 0.908 | Western | 0.000 | 0.999 | 0.000 | 0.000 | 0.000 | 0.001 | Western |
| Germany | Bühren | 51.46581 | 9.6778 | 12/18/06 | Berg | 06-170-23 | 6 | 11 | 0.541 | 0.459 | Mixed | 0.001 | 0.734 | 0.001 | 0.227 | 0.001 | 0.036 | Western - Moderate |
| Germany | Bühren | 51.46581 | 9.6778 | 12/18/06 | Berg | 06-170-24 | 6 | 11 | 0.43 | 0.57 | Mixed | 0.000 | 0.896 | 0.001 | 0.078 | 0.000 | 0.025 | Western |
| Germany | Bühren | 51.46581 | 9.6778 | 12/18/06 | Berg | 06-170-25 | 6 | 11 | 0.442 | 0.558 | Mixed | 0.000 | 0.801 | 0.002 | 0.159 | 0.000 | 0.037 | Western |
| Germany | Bühren | 51.46581 | 9.6778 | 12/18/06 | Berg | 06-170-26 | 6 | 11 | 0.215 | 0.785 | Mixed | 0.000 | 0.991 | 0.000 | 0.004 | 0.000 | 0.005 | Western |
| Germany | Bühren | 51.46581 | 9.6778 | 12/18/06 | Berg | 06-170-27 | 6 | 11 | 0.564 | 0.436 | Mixed | 0.002 | 0.183 | 0.009 | 0.744 | 0.007 | 0.055 | F2 - Moderate |
| Germany | Bühren | 51.46581 | 9.6778 | 12/18/06 | Berg | 06-170-28 | 6 | 11 | 0.471 | 0.529 | Mixed | 0.001 | 0.906 | 0.001 | 0.074 | 0.000 | 0.018 | Western |
| Germany | Bühren | 51.46581 | 9.6778 | 12/18/06 | Berg | 06-170-29 | 6 | 11 | 0.253 | 0.747 | Mixed | 0.000 | 0.984 | 0.000 | 0.010 | 0.000 | 0.006 | Western |
| Germany | Bühren | 51.46581 | 9.6778 | 12/18/06 | Berg | 06-170-30 | 6 | 11 | 0.468 | 0.532 | Mixed | 0.000 | 0.929 | 0.001 | 0.050 | 0.000 | 0.021 | Western |
| Germany | Göttingen | 51.54602 | 9.91108 | 1/7/06 | Berg | 06-172-01 | 6 | 12 | 0.067 | 0.933 | Western | 0.000 | 0.998 | 0.000 | 0.001 | 0.000 | 0.002 | Western |
| Germany | Göttingen | 51.54602 | 9.91108 | 1/7/06 | Berg | 06-172-02 | 6 | 12 | 0.367 | 0.633 | Mixed | 0.000 | 0.870 | 0.000 | 0.109 | 0.000 | 0.021 | Western |
| Germany | Göttingen | 51.54602 | 9.91108 | 1/7/06 | Berg | 06-172-03 | 6 | 12 | 0.365 | 0.635 | Mixed | 0.000 | 0.964 | 0.000 | 0.025 | 0.000 | 0.011 | Western |
| Germany | Göttingen | 51.54602 | 9.91108 | 1/7/06 | Berg | 06-172-04 | 6 | 12 | 0.393 | 0.607 | Mixed | 0.000 | 0.870 | 0.000 | 0.113 | 0.000 | 0.017 | Western |
| Germany | Göttingen | 51.54602 | 9.91108 | 1/7/06 | Berg | 06-172-05 | 6 | 12 | 0.152 | 0.848 | Western | 0.000 | 0.997 | 0.000 | 0.001 | 0.000 | 0.002 | Western |
| Germany | Göttingen | 51.54602 | 9.91108 | 1/7/06 | Berg | 06-172-06 | 6 | 12 | 0.123 | 0.877 | Western | 0.000 | 0.997 | 0.000 | 0.001 | 0.000 | 0.002 | Western |
| Germany | Göttingen | 51.54602 | 9.91108 | 1/7/06 | Berg | 06-172-07 | 6 | 12 | 0.307 | 0.693 | Mixed | 0.000 | 0.942 | 0.000 | 0.033 | 0.000 | 0.025 | Western |
| Germany | Göttingen | 51.54602 | 9.91108 | 1/7/06 | Berg | 06-172-08 | 6 | 12 | 0.068 | 0.932 | Western | 0.000 | 0.999 | 0.000 | 0.000 | 0.000 | 0.001 | Western |
| Germany | Göttingen | 51.54602 | 9.91108 | 1/7/06 | Berg | 06-172-09 | 6 | 12 | 0.402 | 0.598 | Mixed | 0.000 | 0.893 | 0.003 | 0.077 | 0.000 | 0.027 | Western |
| Germany | Göttingen | 51.54602 | 9.91108 | 1/7/06 | Berg | 06-172-10 | 6 | 12 | 0.273 | 0.727 | Mixed | 0.000 | 0.990 | 0.000 | 0.005 | 0.000 | 0.005 | Western |
| Germany | Göttingen | 51.54602 | 9.91108 | 1/7/06 | Berg | 06-172-11 | 6 | 12 | 0.172 | 0.828 | Western | 0.000 | 0.992 | 0.000 | 0.002 | 0.000 | 0.006 | Western |
| Germany | Göttingen | 51.54602 | 9.91108 | 1/7/06 | Berg | 06-172-12 | 6 | 12 | 0.557 | 0.443 | Mixed | 0.001 | 0.071 | 0.008 | 0.864 | 0.005 | 0.050 | F2 |
| Germany | Göttingen | 51.54602 | 9.91108 | 1/7/06 | Berg | 06-172-13 | 6 | 12 | 0.236 | 0.764 | Mixed | 0.000 | 0.974 | 0.000 | 0.017 | 0.000 | 0.009 | Western |
| Germany | Göttingen | 51.54602 | 9.91108 | 1/7/06 | Berg | 06-172-14 | 6 | 12 | 0.178 | 0.822 | Western | 0.000 | 0.993 | 0.000 | 0.002 | 0.000 | 0.005 | Western |
| Germany | Göttingen | 51.54602 | 9.91108 | 1/7/06 | Berg | 06-172-15 | 6 | 12 | 0.241 | 0.759 | Mixed | 0.000 | 0.985 | 0.000 | 0.008 | 0.000 | 0.008 | Western |
| Germany | Göttingen | 51.54602 | 9.91108 | 1/7/06 | Berg | 06-172-16 | 6 | 12 | 0.339 | 0.661 | Mixed | 0.000 | 0.912 | 0.000 | 0.068 | 0.000 | 0.019 | Western |
| Germany | Göttingen | 51.54602 | 9.91108 | 1/7/06 | Berg | 06-172-17 | 6 | 12 | 0.444 | 0.556 | Mixed | 0.000 | 0.904 | 0.000 | 0.075 | 0.000 | 0.021 | Western |
| Germany | Göttingen | 51.54602 | 9.91108 | 1/7/06 | Berg | 06-172-18 | 6 | 12 | 0.601 | 0.399 | Mixed | 0.004 | 0.252 | 0.037 | 0.639 | 0.012 | 0.056 | F2 - Moderate |
| Germany | Göttingen | 51.54602 | 9.91108 | 1/7/06 | Berg | 06-172-19 | 6 | 12 | 0.302 | 0.698 | Mixed | 0.000 | 0.960 | 0.000 | 0.026 | 0.000 | 0.013 | Western |
| Germany | Göttingen | 51.54602 | 9.91108 | 1/7/06 | Berg | 06-172-20 | 6 | 12 | 0.168 | 0.832 | Western | 0.000 | 0.996 | 0.000 | 0.001 | 0.000 | 0.002 | Western |
| Germany | Göttingen | 51.54602 | 9.91108 | 1/7/06 | Berg | 06-172-21 | 6 | 12 | 0.226 | 0.774 | Mixed | 0.000 | 0.985 | 0.000 | 0.009 | 0.000 | 0.006 | Western |
| Germany | Göttingen | 51.54602 | 9.91108 | 1/7/06 | Berg | 06-172-22 | 6 | 12 | 0.581 | 0.419 | Mixed | 0.002 | 0.631 | 0.006 | 0.322 | 0.003 | 0.037 | Western - Moderate |
| Germany | Göttingen | 51.54602 | 9.91108 | 1/7/06 | Berg | 06-172-23 | 6 | 12 | 0.561 | 0.439 | Mixed | 0.005 | 0.274 | 0.001 | 0.684 | 0.003 | 0.032 | F2 - Moderate |
| Germany | Göttingen | 51.54602 | 9.91108 | 1/7/06 | Berg | 06-172-24 | 6 | 12 | 0.321 | 0.679 | Mixed | 0.000 | 0.985 | 0.000 | 0.009 | 0.000 | 0.006 | Western |
| Germany | Göttingen | 51.54602 | 9.91108 | 1/7/06 | Berg | 06-172-25 | 6 | 12 | 0.106 | 0.894 | Western | 0.000 | 0.998 | 0.000 | 0.000 | 0.000 | 0.002 | Western |
| Germany | Göttingen | 51.54602 | 9.91108 | 1/7/06 | Berg | 06-172-26 | 6 | 12 | 0.464 | 0.536 | Mixed | 0.000 | 0.579 | 0.001 | 0.365 | 0.000 | 0.055 | Western - Moderate |
| Germany | Göttingen | 51.54602 | 9.91108 | 1/7/06 | Berg | 06-172-27 | 6 | 12 | 0.54 | 0.46 | Mixed | 0.001 | 0.321 | 0.005 | 0.626 | 0.003 | 0.045 | F2 - Moderate |
| Germany | Göttingen | 51.54602 | 9.91108 | 1/7/06 | Berg | 06-172-28 | 6 | 12 | 0.582 | 0.418 | Mixed | 0.004 | 0.416 | 0.011 | 0.517 | 0.007 | 0.045 | F2 - Moderate |
| Germany | Göttingen | 51.54602 | 9.91108 | 1/7/06 | Berg | 06-172-29 | 6 | 12 | 0.61 | 0.39 | Mixed | 0.003 | 0.630 | 0.006 | 0.325 | 0.004 | 0.032 | Western - Moderate |
| Germany | Göttingen | 51.54602 | 9.91108 | 1/7/06 | Berg | 06-172-30 | 6 | 12 | 0.449 | 0.551 | Mixed | 0.000 | 0.708 | 0.001 | 0.249 | 0.000 | 0.042 | Western - Moderate |
| Germany | Reinhardshagen | 51.47625 | 9.515373 | 11/22/06 | Berg | 06-168-01 | 6 | 13 | 0.612 | 0.388 | Mixed | 0.002 | 0.329 | 0.013 | 0.605 | 0.009 | 0.043 | F2 - Moderate |
| Germany | Reinhardshagen | 51.47625 | 9.515373 | 11/22/06 | Berg | 06-168-02 | 6 | 13 | 0.559 | 0.441 | Mixed | 0.001 | 0.275 | 0.009 | 0.653 | 0.004 | 0.059 | F2 - Moderate |
| Germany | Reinhardshagen | 51.47625 | 9.515373 | 11/22/06 | Berg | 06-168-03 | 6 | 13 | 0.5 | 0.5 | Mixed | 0.000 | 0.580 | 0.003 | 0.373 | 0.002 | 0.043 | Western - Moderate |
| Germany | Reinhardshagen | 51.47625 | 9.515373 | 11/22/06 | Berg | 06-168-04 | 6 | 13 | 0.423 | 0.577 | Mixed | 0.000 | 0.838 | 0.000 | 0.137 | 0.000 | 0.024 | Western |
| Germany | Reinhardshagen | 51.47625 | 9.515373 | 11/22/06 | Berg | 06-168-05 | 6 | 13 | 0.519 | 0.481 | Mixed | 0.000 | 0.820 | 0.003 | 0.142 | 0.001 | 0.034 | Western |
| Germany | Reinhardshagen | 51.47625 | 9.515373 | 11/22/06 | Berg | 06-168-06 | 6 | 13 | 0.316 | 0.684 | Mixed | 0.000 | 0.985 | 0.000 | 0.007 | 0.000 | 0.008 | Western |
| Germany | Reinhardshagen | 51.47625 | 9.515373 | 11/22/06 | Berg | 06-168-07 | 6 | 13 | 0.413 | 0.587 | Mixed | 0.000 | 0.839 | 0.000 | 0.132 | 0.000 | 0.029 | Western |
| Germany | Reinhardshagen | 51.47625 | 9.515373 | 11/22/06 | Berg | 06-168-08 | 6 | 13 | 0.283 | 0.717 | Mixed | 0.000 | 0.960 | 0.000 | 0.029 | 0.000 | 0.011 | Western |
| Germany | Reinhardshagen | 51.47625 | 9.515373 | 11/22/06 | Berg | 06-168-09 | 6 | 13 | 0.314 | 0.686 | Mixed | 0.000 | 0.976 | 0.000 | 0.012 | 0.000 | 0.011 | Western |
| Germany | Reinhardshagen | 51.47625 | 9.515373 | 11/22/06 | Berg | 06-168-10 | 6 | 13 | 0.45 | 0.55 | Mixed | 0.000 | 0.681 | 0.003 | 0.258 | 0.001 | 0.056 | Western - Moderate |
| Germany | Reinhardshagen | 51.47625 | 9.515373 | 11/22/06 | Berg | 06-168-11 | 6 | 13 | 0.273 | 0.727 | Mixed | 0.000 | 0.972 | 0.000 | 0.020 | 0.000 | 0.008 | Western |
| Germany | Reinhardshagen | 51.47625 | 9.515373 | 11/22/06 | Berg | 06-168-12 | 6 | 13 | 0.093 | 0.907 | Western | 0.000 | 0.998 | 0.000 | 0.001 | 0.000 | 0.002 | Western |
| Germany | Reinhardshagen | 51.47625 | 9.515373 | 11/22/06 | Berg | 06-168-13 | 6 | 13 | 0.486 | 0.514 | Mixed | 0.000 | 0.803 | 0.011 | 0.137 | 0.001 | 0.049 | Western |
| Germany | Reinhardshagen | 51.47625 | 9.515373 | 11/22/06 | Berg | 06-168-14 | 6 | 13 | 0.135 | 0.865 | Western | 0.000 | 0.994 | 0.000 | 0.002 | 0.000 | 0.004 | Western |
| Germany | Reinhardshagen | 51.47625 | 9.515373 | 11/22/06 | Berg | 06-168-15 | 6 | 13 | 0.299 | 0.701 | Mixed | 0.000 | 0.930 | 0.000 | 0.055 | 0.000 | 0.015 | Western |
| Germany | Reinhardshagen | 51.47625 | 9.515373 | 11/22/06 | Berg | 06-168-16 | 6 | 13 | 0.337 | 0.663 | Mixed | 0.000 | 0.972 | 0.000 | 0.019 | 0.000 | 0.010 | Western |
| Germany | Reinhardshagen | 51.47625 | 9.515373 | 11/22/06 | Berg | 06-168-17 | 6 | 13 | 0.442 | 0.558 | Mixed | 0.000 | 0.953 | 0.000 | 0.034 | 0.000 | 0.012 | Western |
| Germany | Reinhardshagen | 51.47625 | 9.515373 | 11/22/06 | Berg | 06-168-18 | 6 | 13 | 0.262 | 0.738 | Mixed | 0.000 | 0.966 | 0.000 | 0.017 | 0.000 | 0.017 | Western |
| Germany | Reinhardshagen | 51.47625 | 9.515373 | 11/22/06 | Berg | 06-168-19 | 6 | 13 | 0.241 | 0.759 | Mixed | 0.000 | 0.986 | 0.000 | 0.008 | 0.000 | 0.006 | Western |
| Germany | Reinhardshagen | 51.47625 | 9.515373 | 11/22/06 | Berg | 06-168-20 | 6 | 13 | 0.534 | 0.466 | Mixed | 0.000 | 0.699 | 0.001 | 0.266 | 0.000 | 0.033 | Western - Moderate |
| Germany | Reinhardshagen | 51.47625 | 9.515373 | 11/22/06 | Berg | 06-168-21 | 6 | 13 | 0.27 | 0.73 | Mixed | 0.000 | 0.988 | 0.000 | 0.005 | 0.000 | 0.007 | Western |
| Germany | Reinhardshagen | 51.47625 | 9.515373 | 11/22/06 | Berg | 06-168-22 | 6 | 13 | 0.295 | 0.705 | Mixed | 0.000 | 0.954 | 0.000 | 0.029 | 0.000 | 0.018 | Western |
| Germany | Reinhardshagen | 51.47625 | 9.515373 | 11/22/06 | Berg | 06-168-23 | 6 | 13 | 0.247 | 0.753 | Mixed | 0.000 | 0.991 | 0.000 | 0.004 | 0.000 | 0.005 | Western |
| Germany | Reinhardshagen | 51.47625 | 9.515373 | 11/22/06 | Berg | 06-168-24 | 6 | 13 | 0.446 | 0.554 | Mixed | 0.000 | 0.746 | 0.001 | 0.220 | 0.000 | 0.032 | Western - Moderate |
| Germany | Reinhardshagen | 51.47625 | 9.515373 | 11/22/06 | Berg | 06-168-25 | 6 | 13 | 0.51 | 0.49 | Mixed | 0.000 | 0.409 | 0.019 | 0.495 | 0.004 | 0.073 | F2 - Moderate |
| Germany | Reinhardshagen | 51.47625 | 9.515373 | 11/22/06 | Berg | 06-168-26 | 6 | 13 | 0.776 | 0.224 | Mixed | 0.187 | 0.046 | 0.002 | 0.735 | 0.019 | 0.011 | F2 - Moderate |
| Germany | Reinhardshagen | 51.47625 | 9.515373 | 11/22/06 | Berg | 06-168-27 | 6 | 13 | 0.234 | 0.766 | Mixed | 0.000 | 0.986 | 0.000 | 0.009 | 0.000 | 0.005 | Western |
| Germany | Reinhardshagen | 51.47625 | 9.515373 | 11/22/06 | Berg | 06-168-28 | 6 | 13 | 0.531 | 0.469 | Mixed | 0.000 | 0.934 | 0.001 | 0.052 | 0.000 | 0.013 | Western |
| Germany | Reinhardshagen | 51.47625 | 9.515373 | 11/22/06 | Berg | 06-168-29 | 6 | 13 | 0.099 | 0.901 | Western | 0.000 | 0.998 | 0.000 | 0.000 | 0.000 | 0.002 | Western |
| Germany | Reinhardshagen | 51.47625 | 9.515373 | 11/22/06 | Berg | 06-168-30 | 6 | 13 | 0.427 | 0.573 | Mixed | 0.000 | 0.862 | 0.001 | 0.104 | 0.000 | 0.033 | Western |
| Germany | Schlüchtern | 50.24254 | 9.45378 | 12/20/06 | Berg | 06-171-01 | 6 | 14 | 0.098 | 0.902 | Western | 0.000 | 0.992 | 0.000 | 0.004 | 0.000 | 0.004 | Western |
| Germany | Schlüchtern | 50.24254 | 9.45378 | 12/20/06 | Berg | 06-171-02 | 6 | 14 | 0.378 | 0.622 | Mixed | 0.000 | 0.864 | 0.002 | 0.101 | 0.000 | 0.032 | Western |
| Germany | Schlüchtern | 50.24254 | 9.45378 | 12/20/06 | Berg | 06-171-03 | 6 | 14 | 0.376 | 0.624 | Mixed | 0.000 | 0.972 | 0.000 | 0.014 | 0.000 | 0.014 | Western |
| Germany | Schlüchtern | 50.24254 | 9.45378 | 12/20/06 | Berg | 06-171-04 | 6 | 14 | 0.426 | 0.574 | Mixed | 0.000 | 0.886 | 0.001 | 0.091 | 0.000 | 0.021 | Western |
| Germany | Schlüchtern | 50.24254 | 9.45378 | 12/20/06 | Berg | 06-171-05 | 6 | 14 | 0.716 | 0.284 | Mixed | 0.025 | 0.125 | 0.021 | 0.773 | 0.028 | 0.029 | F2 - Moderate |
| Germany | Schlüchtern | 50.24254 | 9.45378 | 12/20/06 | Berg | 06-171-06 | 6 | 14 | 0.275 | 0.725 | Mixed | 0.000 | 0.936 | 0.000 | 0.049 | 0.000 | 0.015 | Western |
| Germany | Schlüchtern | 50.24254 | 9.45378 | 12/20/06 | Berg | 06-171-07 | 6 | 14 | 0.401 | 0.599 | Mixed | 0.000 | 0.304 | 0.010 | 0.509 | 0.001 | 0.176 | F2 - Moderate |
| Germany | Schlüchtern | 50.24254 | 9.45378 | 12/20/06 | Berg | 06-171-08 | 6 | 14 | 0.312 | 0.688 | Mixed | 0.000 | 0.977 | 0.000 | 0.016 | 0.000 | 0.007 | Western |
| Germany | Schlüchtern | 50.24254 | 9.45378 | 12/20/06 | Berg | 06-171-09 | 6 | 14 | 0.108 | 0.892 | Western | 0.000 | 0.996 | 0.000 | 0.002 | 0.000 | 0.003 | Western |
| Germany | Schlüchtern | 50.24254 | 9.45378 | 12/20/06 | Berg | 06-171-10 | 6 | 14 | 0.549 | 0.451 | Mixed | 0.002 | 0.513 | 0.003 | 0.440 | 0.002 | 0.040 | Western - Moderate |
| Germany | Schlüchtern | 50.24254 | 9.45378 | 12/20/06 | Berg | 06-171-11 | 6 | 14 | 0.196 | 0.804 | Western | 0.000 | 0.993 | 0.000 | 0.003 | 0.000 | 0.004 | Western |
| Germany | Schlüchtern | 50.24254 | 9.45378 | 12/20/06 | Berg | 06-171-12 | 6 | 14 | 0.325 | 0.675 | Mixed | 0.000 | 0.964 | 0.001 | 0.018 | 0.000 | 0.018 | Western |
| Germany | Schlüchtern | 50.24254 | 9.45378 | 12/20/06 | Berg | 06-171-13 | 6 | 14 | 0.175 | 0.825 | Western | 0.000 | 0.994 | 0.000 | 0.002 | 0.000 | 0.004 | Western |
| Germany | Schlüchtern | 50.24254 | 9.45378 | 12/20/06 | Berg | 06-171-14 | 6 | 14 | 0.077 | 0.923 | Western | 0.000 | 0.999 | 0.000 | 0.000 | 0.000 | 0.001 | Western |
| Germany | Schlüchtern | 50.24254 | 9.45378 | 12/20/06 | Berg | 06-171-15 | 6 | 14 | 0.522 | 0.478 | Mixed | 0.001 | 0.571 | 0.005 | 0.376 | 0.002 | 0.045 | Western - Moderate |
| Germany | Schlüchtern | 50.24254 | 9.45378 | 12/20/06 | Berg | 06-171-16 | 6 | 14 | 0.38 | 0.62 | Mixed | 0.000 | 0.726 | 0.000 | 0.250 | 0.000 | 0.024 | Western - Moderate |
| Germany | Schlüchtern | 50.24254 | 9.45378 | 12/20/06 | Berg | 06-171-17 | 6 | 14 | 0.481 | 0.519 | Mixed | 0.000 | 0.816 | 0.002 | 0.152 | 0.001 | 0.030 | Western |
| Germany | Schlüchtern | 50.24254 | 9.45378 | 12/20/06 | Berg | 06-171-18 | 6 | 14 | 0.404 | 0.596 | Mixed | 0.000 | 0.937 | 0.000 | 0.049 | 0.000 | 0.014 | Western |
| Germany | Well am Rhein | 47.62277778 | 7.585 | 12/4/08 | Marc Kenis | 08-317-01 | 15 |  | 0.315 | 0.685 | Mixed | 0.000 | 0.964 | 0.000 | 0.021 | 0.000 | 0.015 | Western |
| Germany | Well am Rhein | 47.62277778 | 7.585 | 12/4/08 | Marc Kenis | 08-317-03 | 15 |  | 0.142 | 0.858 | Western | 0.000 | 0.992 | 0.000 | 0.004 | 0.000 | 0.004 | Western |
| Germany | Well am Rhein | 47.62277778 | 7.585 | 12/4/08 | Marc Kenis | 08-317-04 | 15 |  | 0.093 | 0.907 | Western | 0.000 | 0.997 | 0.000 | 0.001 | 0.000 | 0.002 | Western |
| Italy | Bernardi | 45.6235 | 11.1329 | 12/8/13 | Andrea Battisti | 14-036-02 | 7 |  | 0.842 | 0.158 | Eastern | 0.421 | 0.038 | 0.003 | 0.507 | 0.022 | 0.008 | F2 - Moderate |
| Italy | Bernardi | 45.6235 | 11.1329 | 12/8/13 | Andrea Battisti | 14-036-04 | 7 |  | 0.777 | 0.223 | Mixed | 0.073 | 0.296 | 0.012 | 0.571 | 0.019 | 0.029 | F2 - Moderate |
| Italy | Tregnago | 45.5218 | 11.1617 | 12/11/13 | Andrea Battisti | 14-037-01 | 7 | 15 | 0.694 | 0.306 | Mixed | 0.062 | 0.347 | 0.010 | 0.536 | 0.013 | 0.031 | F2 - Moderate |
| Italy | Tregnago | 45.5218 | 11.1617 | 12/11/13 | Andrea Battisti | 14-037-02 | 7 | 15 | 0.388 | 0.612 | Mixed | 0.000 | 0.932 | 0.001 | 0.052 | 0.000 | 0.015 | Western |
| Italy | Tregnago | 45.5218 | 11.1617 | 12/11/13 | Andrea Battisti | 14-037-04 | 7 | 15 | 0.9 | 0.1 | Eastern | 0.755 | 0.000 | 0.001 | 0.217 | 0.026 | 0.001 | Eastern - Moderate |
| Italy | Tregnago | 45.5218 | 11.1617 | 12/11/13 | Andrea Battisti | 14-037-05 | 7 | 15 | 0.695 | 0.305 | Mixed | 0.038 | 0.260 | 0.006 | 0.656 | 0.011 | 0.030 | F2 - Moderate |
| Italy | Tregnago | 45.5218 | 11.1617 | 12/11/13 | Andrea Battisti | 14-038-01 | 7 | 15 | 0.05 | 0.95 | Western | 0.000 | 0.999 | 0.000 | 0.000 | 0.000 | 0.001 | Western |
| Italy | Tregnago | 45.5218 | 11.1617 | 12/11/13 | Andrea Battisti | 14-038-02 | 7 | 15 | 0.027 | 0.973 | Western | 0.000 | 1.000 | 0.000 | 0.000 | 0.000 | 0.000 | Western |
| Italy | Tregnago | 45.5218 | 11.1617 | 12/11/13 | Andrea Battisti | 14-038-03 | 7 | 15 | 0.208 | 0.792 | Mixed | 0.000 | 0.980 | 0.000 | 0.011 | 0.000 | 0.009 | Western |
| Italy | Tregnago | 45.5218 | 11.1617 | 12/11/13 | Andrea Battisti | 14-038-04 | 7 | 15 | 0.167 | 0.833 | Western | 0.000 | 0.978 | 0.000 | 0.013 | 0.000 | 0.009 | Western |
| Italy | Tregnago | 45.5218 | 11.1617 | 12/11/13 | Andrea Battisti | 14-038-05 | 7 | 15 | 0.033 | 0.967 | Western | 0.000 | 1.000 | 0.000 | 0.000 | 0.000 | 0.000 | Western |
| Italy | Tregnago | 45.5218 | 11.1617 | 12/11/13 | Andrea Battisti | 14-038-06 | 7 | 15 | 0.061 | 0.939 | Western | 0.000 | 0.999 | 0.000 | 0.000 | 0.000 | 0.001 | Western |
| Italy | Tregnago | 45.5218 | 11.1617 | 12/11/13 | Andrea Battisti | 14-038-07 | 7 | 15 | 0.055 | 0.945 | Western | 0.000 | 1.000 | 0.000 | 0.000 | 0.000 | 0.000 | Western |
| Italy | Tregnago | 45.5218 | 11.1617 | 12/11/13 | Andrea Battisti | 14-038-08 | 7 | 15 | 0.158 | 0.842 | Western | 0.000 | 0.998 | 0.000 | 0.000 | 0.000 | 0.002 | Western |
| Italy | Tregnago | 45.5218 | 11.1617 | 12/11/13 | Andrea Battisti | 14-038-09 | 7 | 15 | 0.079 | 0.921 | Western | 0.000 | 0.999 | 0.000 | 0.000 | 0.000 | 0.001 | Western |
| Norway | Tromsø | 69.650836 | 18.954077 | 6/26/09 | Joseph Elkinton | 11-415-01 | 8 | 16 | 0.293 | 0.707 | Mixed | 0.000 | 0.929 | 0.000 | 0.052 | 0.000 | 0.019 | Western |
| Norway | Tromsø | 69.650836 | 18.954077 | 6/26/09 | Joseph Elkinton | 11-415-02 | 8 | 16 | 0.125 | 0.875 | Western | 0.000 | 0.998 | 0.000 | 0.000 | 0.000 | 0.002 | Western |
| Norway | Tromsø | 69.650836 | 18.954077 | 6/26/09 | Joseph Elkinton | 11-415-03 | 8 | 16 | 0.06 | 0.94 | Western | 0.000 | 0.998 | 0.000 | 0.000 | 0.000 | 0.002 | Western |
| Norway | Tromsø | 69.650836 | 18.954077 | 6/26/09 | Joseph Elkinton | 11-415-04 | 8 | 16 | 0.351 | 0.649 | Mixed | 0.000 | 0.766 | 0.001 | 0.161 | 0.000 | 0.073 | Western - Moderate |
| Norway | Tromsø | 69.650836 | 18.954077 | 6/26/09 | Joseph Elkinton | 11-415-05 | 8 | 16 | 0.57 | 0.43 | Mixed | 0.002 | 0.641 | 0.004 | 0.317 | 0.003 | 0.034 | Western - Moderate |
| Norway | Tromsø | 69.650836 | 18.954077 | 6/26/09 | Joseph Elkinton | 11-415-06 | 8 | 16 | 0.123 | 0.877 | Western | 0.000 | 0.995 | 0.000 | 0.002 | 0.000 | 0.003 | Western |
| Norway | Tromsø | 69.650836 | 18.954077 | 6/26/09 | Joseph Elkinton | 11-415-07 | 8 | 16 | 0.062 | 0.938 | Western | 0.000 | 0.998 | 0.000 | 0.000 | 0.000 | 0.001 | Western |
| Norway | Tromsø | 69.650836 | 18.954077 | 6/26/09 | Joseph Elkinton | 11-415-08 | 8 | 16 | 0.141 | 0.859 | Western | 0.000 | 0.987 | 0.000 | 0.007 | 0.000 | 0.006 | Western |
| Norway | Tromsø | 69.650836 | 18.954077 | 6/26/09 | Joseph Elkinton | 11-415-09 | 8 | 16 | 0.079 | 0.921 | Western | 0.000 | 0.998 | 0.000 | 0.000 | 0.000 | 0.002 | Western |
| Norway | Tromsø | 69.650836 | 18.954077 | 6/26/09 | Joseph Elkinton | 11-415-10 | 8 | 16 | 0.2 | 0.8 | Western | 0.000 | 0.979 | 0.000 | 0.012 | 0.000 | 0.009 | Western |
| Norway | Tromsø | 69.650836 | 18.954077 | 6/26/09 | Joseph Elkinton | 11-416-01 | 8 | 16 | 0.215 | 0.785 | Mixed | 0.000 | 0.985 | 0.000 | 0.006 | 0.000 | 0.008 | Western |
| Norway | Tromsø | 69.650836 | 18.954077 | 6/26/09 | Joseph Elkinton | 11-416-02 | 8 | 16 | 0.284 | 0.716 | Mixed | 0.000 | 0.972 | 0.000 | 0.019 | 0.000 | 0.009 | Western |
| Norway | Tromsø | 69.650836 | 18.954077 | 6/26/09 | Joseph Elkinton | 11-416-03 | 8 | 16 | 0.228 | 0.772 | Mixed | 0.000 | 0.986 | 0.000 | 0.008 | 0.000 | 0.006 | Western |
| Norway | Tromsø | 69.650836 | 18.954077 | 6/26/09 | Joseph Elkinton | 11-416-04 | 8 | 16 | 0.374 | 0.626 | Mixed | 0.000 | 0.967 | 0.000 | 0.019 | 0.000 | 0.013 | Western |
| Norway | Tromsø | 69.650836 | 18.954077 | 6/26/09 | Joseph Elkinton | 11-416-05 | 8 | 16 | 0.09 | 0.91 | Western | 0.000 | 0.993 | 0.000 | 0.004 | 0.000 | 0.003 | Western |
| Norway | Tromsø | 69.650836 | 18.954077 | 6/26/09 | Joseph Elkinton | 11-416-06 | 8 | 16 | 0.214 | 0.786 | Mixed | 0.000 | 0.996 | 0.000 | 0.001 | 0.000 | 0.003 | Western |
| Norway | Tromsø | 69.650836 | 18.954077 | 6/26/09 | Joseph Elkinton | 11-416-07 | 8 | 16 | 0.29 | 0.71 | Mixed | 0.000 | 0.949 | 0.000 | 0.038 | 0.000 | 0.013 | Western |
| Norway | Tromsø | 69.650836 | 18.954077 | 6/26/09 | Joseph Elkinton | 11-416-08 | 8 | 16 | 0.322 | 0.678 | Mixed | 0.000 | 0.887 | 0.000 | 0.082 | 0.000 | 0.031 | Western |
| Norway | Tromsø | 69.650836 | 18.954077 | 6/26/09 | Joseph Elkinton | 11-416-09 | 8 | 16 | 0.087 | 0.913 | Western | 0.000 | 0.997 | 0.000 | 0.001 | 0.000 | 0.002 | Western |
| Norway | Tromsø | 69.650836 | 18.954077 | 6/26/09 | Joseph Elkinton | 11-416-10 | 8 | 16 | 0.103 | 0.897 | Western | 0.000 | 0.996 | 0.000 | 0.001 | 0.000 | 0.003 | Western |
| Norway | Tromsø | 69.650836 | 18.954077 | 6/26/09 | Joseph Elkinton | 11-416-11 | 8 | 16 | 0.158 | 0.842 | Western | 0.000 | 0.996 | 0.000 | 0.001 | 0.000 | 0.003 | Western |
| Norway | Tromsø | 69.650836 | 18.954077 | 6/26/09 | Joseph Elkinton | 11-417-01 | 8 | 16 | 0.103 | 0.897 | Western | 0.000 | 0.996 | 0.000 | 0.001 | 0.000 | 0.002 | Western |
| Norway | Tromsø | 69.650836 | 18.954077 | 6/26/09 | Joseph Elkinton | 11-417-02 | 8 | 16 | 0.143 | 0.857 | Western | 0.000 | 0.993 | 0.000 | 0.003 | 0.000 | 0.004 | Western |
| Norway | Tromsø | 69.650836 | 18.954077 | 6/26/09 | Joseph Elkinton | 11-417-03 | 8 | 16 | 0.53 | 0.47 | Mixed | 0.000 | 0.356 | 0.002 | 0.600 | 0.002 | 0.040 | F2 - Moderate |
| Norway | Tromsø | 69.650836 | 18.954077 | 6/26/09 | Joseph Elkinton | 11-417-04 | 8 | 16 | 0.208 | 0.792 | Mixed | 0.000 | 0.990 | 0.000 | 0.004 | 0.000 | 0.006 | Western |
| Norway | Tromsø | 69.650836 | 18.954077 | 6/26/09 | Joseph Elkinton | 11-417-05 | 8 | 16 | 0.113 | 0.887 | Western | 0.000 | 0.995 | 0.000 | 0.002 | 0.000 | 0.003 | Western |
| Norway | Tromsø | 69.650836 | 18.954077 | 6/26/09 | Joseph Elkinton | 11-417-06 | 8 | 16 | 0.57 | 0.43 | Mixed | 0.001 | 0.350 | 0.010 | 0.585 | 0.005 | 0.049 | F2 - Moderate |
| Norway | Tromsø | 69.650836 | 18.954077 | 6/26/09 | Joseph Elkinton | 11-417-07 | 8 | 16 | 0.285 | 0.715 | Mixed | 0.000 | 0.973 | 0.000 | 0.017 | 0.000 | 0.010 | Western |
| Norway | Tromsø | 69.650836 | 18.954077 | 6/26/09 | Joseph Elkinton | 11-417-08 | 8 | 16 | 0.221 | 0.779 | Mixed | 0.000 | 0.936 | 0.000 | 0.039 | 0.000 | 0.024 | Western |
| Norway | Tromsø | 69.650836 | 18.954077 |  | Joesph Elkinton | 16-001_01 | 8 | 16 | 0.034 | 0.966 | Western | 0.000 | 1.000 | 0.000 | 0.000 | 0.000 | 0.000 | Western |
| Poland | Bialostocka | 53.302084 | 23.289745 | 11/16/06 | L. Sukovata | 06-186-11 | 9 | 17 | 0.865 | 0.135 | Eastern | 0.660 | 0.000 | 0.000 | 0.319 | 0.021 | 0.000 | Eastern - Moderate |
| Poland | Bialostocka | 53.302084 | 23.289745 | 11/16/06 | L. Sukovata | 06-186-12 | 9 | 17 | 0.874 | 0.126 | Eastern | 0.818 | 0.000 | 0.000 | 0.156 | 0.026 | 0.000 | Eastern |
| Poland | Bialostocka | 53.302084 | 23.289745 | 11/16/06 | L. Sukovata | 06-186-13 | 9 | 17 | 0.845 | 0.155 | Eastern | 0.719 | 0.000 | 0.002 | 0.243 | 0.035 | 0.001 | Eastern - Moderate |
| Poland | Bialostocka | 53.302084 | 23.289745 | 11/16/06 | L. Sukovata | 06-186-14 | 9 | 17 | 0.967 | 0.033 | Eastern | 0.993 | 0.000 | 0.000 | 0.004 | 0.003 | 0.000 | Eastern |
| Poland | Bialostocka | 53.302084 | 23.289745 | 11/16/06 | L. Sukovata | 06-186-15 | 9 | 17 | 0.935 | 0.065 | Eastern | 0.971 | 0.000 | 0.000 | 0.021 | 0.008 | 0.000 | Eastern |
| Poland | Bialostocka | 53.302084 | 23.289745 | 11/16/06 | L. Sukovata | 06-187-01 | 9 | 17 | 0.924 | 0.076 | Eastern | 0.963 | 0.000 | 0.000 | 0.027 | 0.010 | 0.000 | Eastern |
| Poland | Bialostocka | 53.302084 | 23.289745 | 11/16/06 | L. Sukovata | 06-187-02 | 9 | 17 | 0.954 | 0.046 | Eastern | 0.986 | 0.000 | 0.000 | 0.009 | 0.005 | 0.000 | Eastern |
| Poland | Bialostocka | 53.302084 | 23.289745 | 11/16/06 | L. Sukovata | 06-187-03 | 9 | 17 | 0.971 | 0.029 | Eastern | 0.998 | 0.000 | 0.000 | 0.001 | 0.001 | 0.000 | Eastern |
| Poland | Bialostocka | 53.302084 | 23.289745 | 11/16/06 | L. Sukovata | 06-187-04 | 9 | 17 | 0.971 | 0.029 | Eastern | 0.996 | 0.000 | 0.000 | 0.002 | 0.002 | 0.000 | Eastern |
| Poland | Bialostocka | 53.302084 | 23.289745 | 11/16/06 | L. Sukovata | 06-187-05 | 9 | 17 | 0.943 | 0.057 | Eastern | 0.958 | 0.000 | 0.000 | 0.030 | 0.012 | 0.000 | Eastern |
| Poland | Bialostocka | 53.302084 | 23.289745 | 11/16/06 | L. Sukovata | 06-187-06 | 9 | 17 | 0.935 | 0.065 | Eastern | 0.981 | 0.000 | 0.000 | 0.010 | 0.009 | 0.000 | Eastern |
| Poland | Bialostocka | 53.302084 | 23.289745 | 11/16/06 | L. Sukovata | 06-187-07 | 9 | 17 | 0.963 | 0.037 | Eastern | 0.991 | 0.000 | 0.000 | 0.006 | 0.003 | 0.000 | Eastern |
| Poland | Bialostocka | 53.302084 | 23.289745 | 11/16/06 | L. Sukovata | 06-187-08 | 9 | 17 | 0.945 | 0.055 | Eastern | 0.980 | 0.000 | 0.000 | 0.013 | 0.006 | 0.000 | Eastern |
| Poland | Bialostocka | 53.302084 | 23.289745 | 11/16/06 | L. Sukovata | 06-187-09 | 9 | 17 | 0.93 | 0.07 | Eastern | 0.957 | 0.000 | 0.000 | 0.035 | 0.007 | 0.000 | Eastern |
| Poland | Bialostocka | 53.302084 | 23.289745 | 11/16/06 | L. Sukovata | 06-187-10 | 9 | 17 | 0.914 | 0.086 | Eastern | 0.943 | 0.000 | 0.000 | 0.043 | 0.014 | 0.000 | Eastern |
| Poland | Bialostocka | 53.302084 | 23.289745 | 11/16/06 | L. Sukovata | 06-187-11 | 9 | 17 | 0.963 | 0.037 | Eastern | 0.994 | 0.000 | 0.000 | 0.004 | 0.003 | 0.000 | Eastern |
| Poland | Bialostocka | 53.302084 | 23.289745 | 11/16/06 | L. Sukovata | 06-187-12 | 9 | 17 | 0.931 | 0.069 | Eastern | 0.880 | 0.000 | 0.002 | 0.096 | 0.021 | 0.001 | Eastern |
| Poland | Bialostocka | 53.302084 | 23.289745 | 11/16/06 | L. Sukovata | 06-187-13 | 9 | 17 | 0.839 | 0.161 | Eastern | 0.498 | 0.000 | 0.001 | 0.450 | 0.049 | 0.001 | Eastern - Moderate |
| Poland | Bialostocka | 53.302084 | 23.289745 | 11/16/06 | L. Sukovata | 06-187-14 | 9 | 17 | 0.976 | 0.024 | Eastern | 0.999 | 0.000 | 0.000 | 0.001 | 0.001 | 0.000 | Eastern |
| Poland | Bialostocka | 53.302084 | 23.289745 | 11/16/06 | L. Sukovata | 06-187-15 | 9 | 17 | 0.926 | 0.074 | Eastern | 0.902 | 0.000 | 0.001 | 0.074 | 0.023 | 0.000 | Eastern |
| Poland | Koryciny | 52.66611111 | 22.73888889 | 11/16/06 | L. Sukovata | 06-173-01 | 9 |  | 0.971 | 0.029 | Eastern | 0.997 | 0.000 | 0.000 | 0.001 | 0.002 | 0.000 | Eastern |
| Poland | Koryciny | 52.66611111 | 22.73888889 | 11/16/06 | L. Sukovata | 06-173-02 | 9 |  | 0.966 | 0.034 | Eastern | 0.998 | 0.000 | 0.000 | 0.001 | 0.001 | 0.000 | Eastern |
| Poland | Koryciny | 52.66611111 | 22.73888889 | 11/16/06 | L. Sukovata | 06-173-03 | 9 |  | 0.916 | 0.084 | Eastern | 0.882 | 0.000 | 0.002 | 0.099 | 0.016 | 0.001 | Eastern |
| Poland | Koryciny | 52.66611111 | 22.73888889 | 11/16/06 | L. Sukovata | 06-173-04 | 9 |  | 0.957 | 0.043 | Eastern | 0.979 | 0.000 | 0.000 | 0.016 | 0.005 | 0.000 | Eastern |
| Poland | Koryciny | 52.66611111 | 22.73888889 | 11/16/06 | L. Sukovata | 06-173-05 | 9 |  | 0.936 | 0.064 | Eastern | 0.976 | 0.000 | 0.000 | 0.013 | 0.011 | 0.000 | Eastern |
| Poland | Koryciny | 52.66611111 | 22.73888889 | 11/16/06 | L. Sukovata | 06-173-06 | 9 |  | 0.931 | 0.069 | Eastern | 0.972 | 0.000 | 0.000 | 0.020 | 0.008 | 0.000 | Eastern |
| Poland | Koryciny | 52.66611111 | 22.73888889 | 11/16/06 | L. Sukovata | 06-173-07 | 9 |  | 0.877 | 0.123 | Eastern | 0.835 | 0.000 | 0.002 | 0.130 | 0.033 | 0.000 | Eastern |
| Poland | Koryciny | 52.66611111 | 22.73888889 | 11/16/06 | L. Sukovata | 06-173-08 | 9 |  | 0.94 | 0.06 | Eastern | 0.984 | 0.000 | 0.000 | 0.010 | 0.006 | 0.000 | Eastern |
| Poland | Koryciny | 52.66611111 | 22.73888889 | 11/16/06 | L. Sukovata | 06-173-09 | 9 |  | 0.951 | 0.049 | Eastern | 0.990 | 0.000 | 0.000 | 0.006 | 0.004 | 0.000 | Eastern |
| Scotland | Banchory | 57.07527778 | -2.532777778 | 12/4/06 | Adam Vanbergen | 06-208-01 | 10 | 18 | 0.053 | 0.947 | Western | 0.000 | 0.998 | 0.000 | 0.001 | 0.000 | 0.002 | Western |
| Scotland | Banchory | 57.07527778 | -2.532777778 | 12/4/06 | Adam Vanbergen | 06-208-02 | 10 | 18 | 0.034 | 0.966 | Western | 0.000 | 1.000 | 0.000 | 0.000 | 0.000 | 0.000 | Western |
| Scotland | Banchory | 57.07527778 | -2.532777778 | 12/4/06 | Adam Vanbergen | 06-208-03 | 10 | 18 | 0.034 | 0.966 | Western | 0.000 | 1.000 | 0.000 | 0.000 | 0.000 | 0.000 | Western |
| Scotland | Banchory | 57.07527778 | -2.532777778 | 12/4/06 | Adam Vanbergen | 06-208-04 | 10 | 18 | 0.039 | 0.961 | Western | 0.000 | 0.999 | 0.000 | 0.000 | 0.000 | 0.001 | Western |
| Scotland | Banchory | 57.07527778 | -2.532777778 | 12/4/06 | Adam Vanbergen | 06-208-05 | 10 | 18 | 0.025 | 0.975 | Western | 0.000 | 1.000 | 0.000 | 0.000 | 0.000 | 0.000 | Western |
| Scotland | Banchory | 57.07527778 | -2.532777778 | 12/4/06 | Adam Vanbergen | 06-208-06 | 10 | 18 | 0.122 | 0.878 | Western | 0.000 | 0.990 | 0.000 | 0.005 | 0.000 | 0.005 | Western |
| Scotland | Banchory | 57.07527778 | -2.532777778 | 12/4/06 | Adam Vanbergen | 06-208-07 | 10 | 18 | 0.024 | 0.976 | Western | 0.000 | 1.000 | 0.000 | 0.000 | 0.000 | 0.000 | Western |
| Scotland | Banchory | 57.07527778 | -2.532777778 | 12/4/06 | Adam Vanbergen | 06-208-08 | 10 | 18 | 0.037 | 0.963 | Western | 0.000 | 0.999 | 0.000 | 0.000 | 0.000 | 0.001 | Western |
| Scotland | Banchory | 57.07527778 | -2.532777778 | 12/4/06 | Adam Vanbergen | 06-208-09 | 10 | 18 | 0.021 | 0.979 | Western | 0.000 | 1.000 | 0.000 | 0.000 | 0.000 | 0.000 | Western |
| Scotland | Banchory | 57.07527778 | -2.532777778 | 12/4/06 | Adam Vanbergen | 06-208-10 | 10 | 18 | 0.029 | 0.971 | Western | 0.000 | 1.000 | 0.000 | 0.000 | 0.000 | 0.000 | Western |
| Scotland | Torphins | 57.07083333 | -2.585833333 | 11/12/06 | Adam Vanbergen | 06-205-01 | 10 | 19 | 0.044 | 0.956 | Western | 0.000 | 1.000 | 0.000 | 0.000 | 0.000 | 0.000 | Western |
| Scotland | Torphins | 57.07083333 | -2.585833333 | 11/12/06 | Adam Vanbergen | 06-205-02 | 10 | 19 | 0.037 | 0.963 | Western | 0.000 | 1.000 | 0.000 | 0.000 | 0.000 | 0.000 | Western |
| Scotland | Torphins | 57.07083333 | -2.585833333 | 11/12/06 | Adam Vanbergen | 06-205-03 | 10 | 19 | 0.099 | 0.901 | Western | 0.000 | 0.996 | 0.000 | 0.001 | 0.000 | 0.003 | Western |
| Scotland | Torphins | 57.07083333 | -2.585833333 | 11/12/06 | Adam Vanbergen | 06-205-04 | 10 | 19 | 0.044 | 0.956 | Western | 0.000 | 0.999 | 0.000 | 0.000 | 0.000 | 0.001 | Western |
| Scotland | Torphins | 57.07083333 | -2.585833333 | 11/12/06 | Adam Vanbergen | 06-205-05 | 10 | 19 | 0.044 | 0.956 | Western | 0.000 | 0.999 | 0.000 | 0.000 | 0.000 | 0.001 | Western |
| Scotland | Torphins | 57.07083333 | -2.585833333 | 11/12/06 | Adam Vanbergen | 06-205-06 | 10 | 19 | 0.034 | 0.966 | Western | 0.000 | 0.999 | 0.000 | 0.000 | 0.000 | 0.001 | Western |
| Scotland | Torphins | 57.07083333 | -2.585833333 | 11/12/06 | Adam Vanbergen | 06-205-07 | 10 | 19 | 0.12 | 0.88 | Western | 0.000 | 0.998 | 0.000 | 0.001 | 0.000 | 0.001 | Western |
| Scotland | Torphins | 57.07083333 | -2.585833333 | 11/12/06 | Adam Vanbergen | 06-205-08 | 10 | 19 | 0.056 | 0.944 | Western | 0.000 | 0.999 | 0.000 | 0.000 | 0.000 | 0.001 | Western |
| Scotland | Torphins | 57.07083333 | -2.585833333 | 11/12/06 | Adam Vanbergen | 06-205-09 | 10 | 19 | 0.033 | 0.967 | Western | 0.000 | 1.000 | 0.000 | 0.000 | 0.000 | 0.000 | Western |
| Scotland | Torphins | 57.07083333 | -2.585833333 | 11/12/06 | Adam Vanbergen | 06-205-10 | 10 | 19 | 0.121 | 0.879 | Western | 0.000 | 0.995 | 0.000 | 0.003 | 0.000 | 0.003 | Western |
| Scotland | Torphins | 57.07083333 | -2.585277778 | 11/12/06 | Adam Vanbergen | 06-206-01 | 10 | 19 | 0.089 | 0.911 | Western | 0.000 | 0.996 | 0.000 | 0.001 | 0.000 | 0.003 | Western |
| Scotland | Torphins | 57.07083333 | -2.585277778 | 11/12/06 | Adam Vanbergen | 06-206-02 | 10 | 19 | 0.034 | 0.966 | Western | 0.000 | 1.000 | 0.000 | 0.000 | 0.000 | 0.000 | Western |
| Scotland | Torphins | 57.07083333 | -2.585277778 | 11/12/06 | Adam Vanbergen | 06-206-03 | 10 | 19 | 0.029 | 0.971 | Western | 0.000 | 1.000 | 0.000 | 0.000 | 0.000 | 0.000 | Western |
| Scotland | Torphins | 57.07083333 | -2.585277778 | 11/12/06 | Adam Vanbergen | 06-206-04 | 10 | 19 | 0.033 | 0.967 | Western | 0.000 | 1.000 | 0.000 | 0.000 | 0.000 | 0.000 | Western |
| Scotland | Torphins | 57.07083333 | -2.585277778 | 11/12/06 | Adam Vanbergen | 06-206-05 | 10 | 19 | 0.031 | 0.969 | Western | 0.000 | 1.000 | 0.000 | 0.000 | 0.000 | 0.000 | Western |
| Scotland | Torphins | 57.07083333 | -2.585277778 | 11/12/06 | Adam Vanbergen | 06-206-06 | 10 | 19 | 0.038 | 0.962 | Western | 0.000 | 1.000 | 0.000 | 0.000 | 0.000 | 0.000 | Western |
| Scotland | Torphins | 57.07083333 | -2.585277778 | 11/12/06 | Adam Vanbergen | 06-206-07 | 10 | 19 | 0.034 | 0.966 | Western | 0.000 | 1.000 | 0.000 | 0.000 | 0.000 | 0.000 | Western |
| Scotland | Torphins | 57.07083333 | -2.585277778 | 11/12/06 | Adam Vanbergen | 06-206-08 | 10 | 19 | 0.052 | 0.948 | Western | 0.000 | 0.999 | 0.000 | 0.000 | 0.000 | 0.001 | Western |
| Scotland | Torphins | 57.07083333 | -2.585277778 | 11/12/06 | Adam Vanbergen | 06-206-09 | 10 | 19 | 0.044 | 0.956 | Western | 0.000 | 0.999 | 0.000 | 0.000 | 0.000 | 0.000 | Western |
| Scotland | Torphins | 57.07083333 | -2.585277778 | 11/12/06 | Adam Vanbergen | 06-206-10 | 10 | 19 | 0.051 | 0.949 | Western | 0.000 | 1.000 | 0.000 | 0.000 | 0.000 | 0.000 | Western |
| Scotland | Torphins | 57.07111111 | -2.585277778 | 11/12/06 | Adam Vanbergen | 06-207-01 | 10 | 19 | 0.039 | 0.961 | Western | 0.000 | 1.000 | 0.000 | 0.000 | 0.000 | 0.000 | Western |
| Scotland | Torphins | 57.07111111 | -2.585277778 | 11/12/06 | Adam Vanbergen | 06-207-02 | 10 | 19 | 0.089 | 0.911 | Western | 0.000 | 0.997 | 0.000 | 0.001 | 0.000 | 0.002 | Western |
| Scotland | Torphins | 57.07111111 | -2.585277778 | 11/12/06 | Adam Vanbergen | 06-207-03 | 10 | 19 | 0.043 | 0.957 | Western | 0.000 | 0.999 | 0.000 | 0.000 | 0.000 | 0.001 | Western |
| Scotland | Torphins | 57.07111111 | -2.585277778 | 11/12/06 | Adam Vanbergen | 06-207-04 | 10 | 19 | 0.069 | 0.931 | Western | 0.000 | 0.999 | 0.000 | 0.000 | 0.000 | 0.001 | Western |
| Scotland | Torphins | 57.07111111 | -2.585277778 | 11/12/06 | Adam Vanbergen | 06-207-05 | 10 | 19 | 0.06 | 0.94 | Western | 0.000 | 0.998 | 0.000 | 0.000 | 0.000 | 0.002 | Western |
| Scotland | Torphins | 57.07111111 | -2.585277778 | 11/12/06 | Adam Vanbergen | 06-207-06 | 10 | 19 | 0.036 | 0.964 | Western | 0.000 | 0.999 | 0.000 | 0.000 | 0.000 | 0.001 | Western |
| Scotland | Torphins | 57.07111111 | -2.585277778 | 11/12/06 | Adam Vanbergen | 06-207-07 | 10 | 19 | 0.046 | 0.954 | Western | 0.000 | 0.999 | 0.000 | 0.000 | 0.000 | 0.001 | Western |
| Scotland | Torphins | 57.07111111 | -2.585277778 | 11/12/06 | Adam Vanbergen | 06-207-08 | 10 | 19 | 0.129 | 0.871 | Western | 0.000 | 0.949 | 0.000 | 0.041 | 0.000 | 0.010 | Western |
| Scotland | Torphins | 57.07111111 | -2.585277778 | 11/12/06 | Adam Vanbergen | 06-207-09 | 10 | 19 | 0.077 | 0.923 | Western | 0.000 | 0.999 | 0.000 | 0.000 | 0.000 | 0.001 | Western |
| Scotland | Torphins | 57.07111111 | -2.585277778 | 11/12/06 | Adam Vanbergen | 06-207-10 | 10 | 19 | 0.033 | 0.967 | Western | 0.000 | 1.000 | 0.000 | 0.000 | 0.000 | 0.000 | Western |
| Serbia | Belgrade | 44.764305 | 20.436431 | 11/27/06 | M. Glavendekić | 06-192-01 | 11 | 20 | 0.87 | 0.13 | Eastern | 0.799 | 0.000 | 0.001 | 0.176 | 0.025 | 0.000 | Eastern - Moderate |
| Serbia | Belgrade | 44.764305 | 20.436431 | 11/27/06 | M. Glavendekić | 06-192-02 | 11 | 20 | 0.958 | 0.042 | Eastern | 0.992 | 0.000 | 0.000 | 0.005 | 0.003 | 0.000 | Eastern |
| Serbia | Belgrade | 44.764305 | 20.436431 | 11/27/06 | M. Glavendekić | 06-192-03 | 11 | 20 | 0.973 | 0.027 | Eastern | 0.999 | 0.000 | 0.000 | 0.000 | 0.001 | 0.000 | Eastern |
| Serbia | Belgrade | 44.764305 | 20.436431 | 11/27/06 | M. Glavendekić | 06-192-04 | 11 | 20 | 0.959 | 0.041 | Eastern | 0.995 | 0.000 | 0.000 | 0.003 | 0.002 | 0.000 | Eastern |
| Serbia | Belgrade | 44.764305 | 20.436431 | 11/27/06 | M. Glavendekić | 06-192-05 | 11 | 20 | 0.931 | 0.069 | Eastern | 0.911 | 0.000 | 0.000 | 0.080 | 0.009 | 0.000 | Eastern |
| Serbia | Belgrade | 44.764305 | 20.436431 | 11/27/06 | M. Glavendekić | 06-192-06 | 11 | 20 | 0.971 | 0.029 | Eastern | 0.999 | 0.000 | 0.000 | 0.000 | 0.001 | 0.000 | Eastern |
| Serbia | Belgrade | 44.764305 | 20.436431 | 11/27/06 | M. Glavendekić | 06-192-07 | 11 | 20 | 0.952 | 0.048 | Eastern | 0.990 | 0.000 | 0.000 | 0.006 | 0.004 | 0.000 | Eastern |
| Serbia | Belgrade | 44.764305 | 20.436431 | 11/27/06 | M. Glavendekić | 06-192-08 | 11 | 20 | 0.961 | 0.039 | Eastern | 0.997 | 0.000 | 0.000 | 0.002 | 0.002 | 0.000 | Eastern |
| Serbia | Belgrade | 44.764305 | 20.436431 | 11/27/06 | M. Glavendekić | 06-192-09 | 11 | 20 | 0.97 | 0.03 | Eastern | 0.999 | 0.000 | 0.000 | 0.000 | 0.001 | 0.000 | Eastern |
| Serbia | Belgrade | 44.764305 | 20.436431 | 11/27/06 | M. Glavendekić | 06-192-10 | 11 | 20 | 0.956 | 0.044 | Eastern | 0.998 | 0.000 | 0.000 | 0.001 | 0.001 | 0.000 | Eastern |
| Serbia | Belgrade | 44.764305 | 20.436431 | 11/27/06 | M. Glavendekić | 06-192-11 | 11 | 20 | 0.809 | 0.191 | Eastern | 0.373 | 0.000 | 0.000 | 0.591 | 0.035 | 0.000 | F2 - Moderate |
| Serbia | Belgrade | 44.764305 | 20.436431 | 11/27/06 | M. Glavendekić | 06-192-12 | 11 | 20 | 0.961 | 0.039 | Eastern | 0.996 | 0.000 | 0.000 | 0.002 | 0.002 | 0.000 | Eastern |
| Serbia | Belgrade | 44.764305 | 20.436431 | 11/27/06 | M. Glavendekić | 06-192-13 | 11 | 20 | 0.958 | 0.042 | Eastern | 0.998 | 0.000 | 0.000 | 0.001 | 0.001 | 0.000 | Eastern |
| Serbia | Belgrade | 44.764305 | 20.436431 | 11/27/06 | M. Glavendekić | 06-192-14 | 11 | 20 | 0.934 | 0.066 | Eastern | 0.983 | 0.000 | 0.000 | 0.008 | 0.008 | 0.000 | Eastern |
| Serbia | Belgrade | 44.764305 | 20.436431 | 11/27/06 | M. Glavendekić | 06-192-15 | 11 | 20 | 0.831 | 0.169 | Eastern | 0.451 | 0.000 | 0.018 | 0.442 | 0.088 | 0.002 | Unassigned |
| Serbia | Pančevo | 44.852359 | 20.653243 | 12/4/06 | M. Glavendekić | 06-189-01 | 11 | 21 | 0.97 | 0.03 | Eastern | 0.999 | 0.000 | 0.000 | 0.000 | 0.001 | 0.000 | Eastern |
| Serbia | Pančevo | 44.852359 | 20.653243 | 12/4/06 | M. Glavendekić | 06-189-02 | 11 | 21 | 0.768 | 0.232 | Mixed | 0.440 | 0.006 | 0.003 | 0.522 | 0.024 | 0.005 | F2 - Moderate |
| Serbia | Pančevo | 44.852359 | 20.653243 | 12/4/06 | M. Glavendekić | 06-189-04 | 11 | 21 | 0.644 | 0.356 | Mixed | 0.013 | 0.541 | 0.003 | 0.405 | 0.004 | 0.034 | Western - Moderate |
| Serbia | Pančevo | 44.852359 | 20.653243 | 12/4/06 | M. Glavendekić | 06-189-05 | 11 | 21 | 0.88 | 0.12 | Eastern | 0.763 | 0.003 | 0.005 | 0.202 | 0.024 | 0.003 | Eastern - Moderate |
| Serbia | Pančevo | 44.852359 | 20.653243 | 12/4/06 | M. Glavendekić | 06-189-06 | 11 | 21 | 0.925 | 0.075 | Eastern | 0.888 | 0.000 | 0.001 | 0.097 | 0.014 | 0.001 | Eastern |
| Serbia | Pančevo | 44.852359 | 20.653243 | 12/4/06 | M. Glavendekić | 06-189-08 | 11 | 21 | 0.893 | 0.107 | Eastern | 0.800 | 0.000 | 0.002 | 0.172 | 0.025 | 0.001 | Eastern - Moderate |
| Serbia | Pančevo | 44.852359 | 20.653243 | 12/4/06 | M. Glavendekić | 06-189-09 | 11 | 21 | 0.792 | 0.208 | Mixed | 0.163 | 0.021 | 0.024 | 0.737 | 0.033 | 0.023 | F2 - Moderate |
| Serbia | Pančevo | 44.852359 | 20.653243 | 12/4/06 | M. Glavendekić | 06-189-10 | 11 | 21 | 0.816 | 0.184 | Eastern | 0.266 | 0.002 | 0.016 | 0.663 | 0.043 | 0.010 | F2 - Moderate |
| Serbia | Pančevo | 44.852359 | 20.653243 | 12/4/06 | M. Glavendekić | 06-189-11 | 11 | 21 | 0.956 | 0.044 | Eastern | 0.976 | 0.000 | 0.000 | 0.018 | 0.006 | 0.000 | Eastern |
| Serbia | Pančevo | 44.852359 | 20.653243 | 12/4/06 | M. Glavendekić | 06-189-12 | 11 | 21 | 0.966 | 0.034 | Eastern | 0.996 | 0.000 | 0.000 | 0.001 | 0.003 | 0.000 | Eastern |
| Serbia | Pančevo | 44.852359 | 20.653243 | 12/4/06 | M. Glavendekić | 06-189-13 | 11 | 21 | 0.888 | 0.112 | Eastern | 0.807 | 0.000 | 0.000 | 0.167 | 0.025 | 0.000 | Eastern |
| Serbia | Pančevo | 44.852359 | 20.653243 | 12/4/06 | M. Glavendekić | 06-189-14 | 11 | 21 | 0.912 | 0.088 | Eastern | 0.920 | 0.000 | 0.000 | 0.069 | 0.011 | 0.000 | Eastern |
| Serbia | Pančevo | 44.852359 | 20.653243 | 12/4/06 | M. Glavendekić | 06-189-15 | 11 | 21 | 0.88 | 0.12 | Eastern | 0.928 | 0.000 | 0.000 | 0.062 | 0.010 | 0.000 | Eastern |
| Serbia | Pančevo | 44.852359 | 20.653243 | 12/4/06 | M. Glavendekić | 06-189-16 | 11 | 21 | 0.97 | 0.03 | Eastern | 0.997 | 0.000 | 0.000 | 0.001 | 0.002 | 0.000 | Eastern |
| Serbia | Pančevo | 44.852359 | 20.653243 | 12/4/06 | M. Glavendekić | 06-189-17 | 11 | 21 | 0.962 | 0.038 | Eastern | 0.994 | 0.000 | 0.000 | 0.003 | 0.002 | 0.000 | Eastern |
| Serbia | Pančevo | 44.852359 | 20.653243 | 12/4/06 | M. Glavendekić | 06-189-18 | 11 | 21 | 0.936 | 0.064 | Eastern | 0.966 | 0.000 | 0.000 | 0.025 | 0.009 | 0.000 | Eastern |
| Serbia | Pančevo | 44.852359 | 20.653243 | 12/4/06 | M. Glavendekić | 06-189-19 | 11 | 21 | 0.92 | 0.08 | Eastern | 0.924 | 0.000 | 0.001 | 0.054 | 0.020 | 0.000 | Eastern |
| Serbia | Pančevo | 44.852359 | 20.653243 | 12/4/06 | M. Glavendekić | 06-189-20 | 11 | 21 | 0.955 | 0.045 | Eastern | 0.988 | 0.000 | 0.000 | 0.008 | 0.004 | 0.000 | Eastern |
| Serbia | Pančevo | 44.875168 | 20.655122 | 11/28/06 | M. Glavendekić | 06-190-01 | 11 | 21 | 0.938 | 0.062 | Eastern | 0.985 | 0.000 | 0.000 | 0.009 | 0.005 | 0.000 | Eastern |
| Serbia | Pančevo | 44.875168 | 20.655122 | 11/28/06 | M. Glavendekić | 06-190-02 | 11 | 21 | 0.939 | 0.061 | Eastern | 0.962 | 0.000 | 0.000 | 0.029 | 0.008 | 0.000 | Eastern |
| Serbia | Pančevo | 44.875168 | 20.655122 | 11/28/06 | M. Glavendekić | 06-190-03 | 11 | 21 | 0.933 | 0.067 | Eastern | 0.964 | 0.000 | 0.000 | 0.028 | 0.008 | 0.000 | Eastern |
| Serbia | Pančevo | 44.875168 | 20.655122 | 11/28/06 | M. Glavendekić | 06-190-04 | 11 | 21 | 0.854 | 0.146 | Eastern | 0.896 | 0.000 | 0.000 | 0.074 | 0.029 | 0.000 | Eastern |
| Serbia | Pančevo | 44.875168 | 20.655122 | 11/28/06 | M. Glavendekić | 06-190-05 | 11 | 21 | 0.953 | 0.047 | Eastern | 0.985 | 0.000 | 0.000 | 0.010 | 0.004 | 0.000 | Eastern |
| Serbia | Pančevo | 44.875168 | 20.655122 | 11/28/06 | M. Glavendekić | 06-190-06 | 11 | 21 | 0.97 | 0.03 | Eastern | 0.999 | 0.000 | 0.000 | 0.000 | 0.001 | 0.000 | Eastern |
| Serbia | Pančevo | 44.875168 | 20.655122 | 11/28/06 | M. Glavendekić | 06-190-07 | 11 | 21 | 0.943 | 0.057 | Eastern | 0.992 | 0.000 | 0.000 | 0.004 | 0.004 | 0.000 | Eastern |
| Serbia | Pančevo | 44.875168 | 20.655122 | 11/28/06 | M. Glavendekić | 06-190-08 | 11 | 21 | 0.954 | 0.046 | Eastern | 0.998 | 0.000 | 0.000 | 0.000 | 0.001 | 0.000 | Eastern |
| Serbia | Pančevo | 44.875168 | 20.655122 | 11/28/06 | M. Glavendekić | 06-190-09 | 11 | 21 | 0.961 | 0.039 | Eastern | 0.992 | 0.000 | 0.000 | 0.005 | 0.003 | 0.000 | Eastern |
| Serbia | Pančevo | 44.875168 | 20.655122 | 11/28/06 | M. Glavendekić | 06-190-10 | 11 | 21 | 0.972 | 0.028 | Eastern | 1.000 | 0.000 | 0.000 | 0.000 | 0.000 | 0.000 | Eastern |
| Serbia | Pančevo | 44.875168 | 20.655122 | 11/28/06 | M. Glavendekić | 06-190-11 | 11 | 21 | 0.962 | 0.038 | Eastern | 0.997 | 0.000 | 0.000 | 0.002 | 0.001 | 0.000 | Eastern |
| Serbia | Pančevo | 44.875168 | 20.655122 | 11/28/06 | M. Glavendekić | 06-190-12 | 11 | 21 | 0.95 | 0.05 | Eastern | 0.983 | 0.000 | 0.000 | 0.013 | 0.005 | 0.000 | Eastern |
| Serbia | Pančevo | 44.875168 | 20.655122 | 11/28/06 | M. Glavendekić | 06-190-13 | 11 | 21 | 0.948 | 0.052 | Eastern | 0.997 | 0.000 | 0.000 | 0.001 | 0.002 | 0.000 | Eastern |
| Serbia | Pančevo | 44.875168 | 20.655122 | 11/28/06 | M. Glavendekić | 06-190-14 | 11 | 21 | 0.903 | 0.097 | Eastern | 0.949 | 0.000 | 0.000 | 0.037 | 0.014 | 0.000 | Eastern |
| Serbia | Pančevo | 44.875168 | 20.655122 | 11/28/06 | M. Glavendekić | 06-190-15 | 11 | 21 | 0.965 | 0.035 | Eastern | 0.998 | 0.000 | 0.000 | 0.001 | 0.002 | 0.000 | Eastern |
| Serbia | Pančevo | 44.875168 | 20.655122 | 11/28/06 | M. Glavendekić | 06-190-17 | 11 | 21 | 0.911 | 0.089 | Eastern | 0.968 | 0.000 | 0.000 | 0.024 | 0.008 | 0.000 | Eastern |
| Serbia | Pančevo | 44.875168 | 20.655122 | 11/28/06 | M. Glavendekić | 06-190-18 | 11 | 21 | 0.941 | 0.059 | Eastern | 0.986 | 0.000 | 0.000 | 0.007 | 0.007 | 0.000 | Eastern |
| Serbia | Pančevo | 44.875168 | 20.655122 | 11/28/06 | M. Glavendekić | 06-190-19 | 11 | 21 | 0.946 | 0.054 | Eastern | 0.980 | 0.000 | 0.000 | 0.014 | 0.006 | 0.000 | Eastern |
| Serbia | Pančevo | 44.875168 | 20.655122 | 11/28/06 | M. Glavendekić | 06-190-20 | 11 | 21 | 0.925 | 0.075 | Eastern | 0.972 | 0.000 | 0.000 | 0.015 | 0.012 | 0.000 | Eastern |
| Slovakia | Banská Štiavnica | 48.45805556 | 18.89638889 | Nov-06 | Marek Turcáni | 06-199-01 | 12 | 22 | 0.646 | 0.354 | Mixed | 0.009 | 0.001 | 0.000 | 0.977 | 0.011 | 0.001 | F2 |
| Slovakia | Banská Štiavnica | 48.45805556 | 18.89638889 | Nov-06 | Marek Turcáni | 06-199-02 | 12 | 22 | 0.6 | 0.4 | Mixed | 0.004 | 0.025 | 0.017 | 0.895 | 0.010 | 0.050 | F2 |
| Slovakia | Banská Štiavnica | 48.45805556 | 18.89638889 | Nov-06 | Marek Turcáni | 06-200-01 | 12 | 22 | 0.954 | 0.046 | Eastern | 0.986 | 0.000 | 0.000 | 0.010 | 0.005 | 0.000 | Eastern |
| Slovakia | Banská Štiavnica | 48.45805556 | 18.89638889 | Nov-06 | Marek Turcáni | 06-200-02 | 12 | 22 | 0.93 | 0.07 | Eastern | 0.972 | 0.000 | 0.000 | 0.017 | 0.010 | 0.000 | Eastern |
| Slovakia | Banská Štiavnica | 48.45805556 | 18.89638889 | Nov-06 | Marek Turcáni | 06-200-03 | 12 | 22 | 0.96 | 0.04 | Eastern | 0.982 | 0.000 | 0.000 | 0.011 | 0.007 | 0.000 | Eastern |
| Slovakia | Banská Štiavnica | 48.45805556 | 18.89638889 | Nov-06 | Marek Turcáni | 06-200-04 | 12 | 22 | 0.66 | 0.34 | Mixed | 0.086 | 0.030 | 0.009 | 0.829 | 0.016 | 0.030 | F2 |
| Slovakia | Banská Štiavnica | 48.45805556 | 18.89638889 | Nov-06 | Marek Turcáni | 06-200-05 | 12 | 22 | 0.915 | 0.085 | Eastern | 0.966 | 0.000 | 0.000 | 0.026 | 0.008 | 0.000 | Eastern |
| Slovakia | Banská Štiavnica | 48.45805556 | 18.89638889 | Nov-06 | Marek Turcáni | 06-200-07 | 12 | 22 | 0.913 | 0.087 | Eastern | 0.931 | 0.000 | 0.000 | 0.055 | 0.013 | 0.000 | Eastern |
| Slovakia | Banská Štiavnica | 48.45805556 | 18.89638889 | Nov-06 | Marek Turcáni | 06-200-08 | 12 | 22 | 0.835 | 0.165 | Eastern | 0.463 | 0.001 | 0.006 | 0.483 | 0.042 | 0.004 | Unassigned |
| Slovakia | Banská Štiavnica | 48.45805556 | 18.89638889 | Nov-06 | Marek Turcáni | 06-200-09 | 12 | 22 | 0.834 | 0.166 | Eastern | 0.559 | 0.001 | 0.002 | 0.403 | 0.034 | 0.002 | Eastern - Moderate |
| Slovakia | Banská Štiavnica | 48.45805556 | 18.89638889 | Nov-06 | Marek Turcáni | 06-200-10 | 12 | 22 | 0.964 | 0.036 | Eastern | 0.995 | 0.000 | 0.000 | 0.002 | 0.003 | 0.000 | Eastern |
| Slovakia | Banská Štiavnica | 48.45805556 | 18.89638889 | Nov-06 | Marek Turcáni | 06-200-12 | 12 | 22 | 0.909 | 0.091 | Eastern | 0.895 | 0.000 | 0.000 | 0.092 | 0.013 | 0.000 | Eastern |
| Slovakia | Banská Štiavnica | 48.45805556 | 18.89638889 | Nov-06 | Marek Turcáni | 06-200-13 | 12 | 22 | 0.975 | 0.025 | Eastern | 0.999 | 0.000 | 0.000 | 0.000 | 0.001 | 0.000 | Eastern |
| Slovakia | Banská Štiavnica | 48.45805556 | 18.89638889 | Nov-06 | Marek Turcáni | 06-200-14 | 12 | 22 | 0.791 | 0.209 | Mixed | 0.274 | 0.037 | 0.005 | 0.646 | 0.028 | 0.011 | F2 - Moderate |
| Slovakia | Banská Štiavnica | 48.45805556 | 18.89638889 | Nov-06 | Marek Turcáni | 06-200-15 | 12 | 22 | 0.84 | 0.16 | Eastern | 0.686 | 0.000 | 0.000 | 0.288 | 0.025 | 0.000 | Eastern - Moderate |
| Slovakia | Banská Štiavnica | 48.45805556 | 18.89638889 | Nov-06 | Marek Turcáni | 06-200-16 | 12 | 22 | 0.94 | 0.06 | Eastern | 0.984 | 0.000 | 0.000 | 0.008 | 0.008 | 0.000 | Eastern |
| Slovakia | Banská Štiavnica | 48.45805556 | 18.89638889 | Nov-06 | Marek Turcáni | 06-200-17 | 12 | 22 | 0.9 | 0.1 | Eastern | 0.889 | 0.000 | 0.000 | 0.085 | 0.025 | 0.000 | Eastern |
| Slovakia | Banská Štiavnica | 48.45805556 | 18.89638889 | Nov-06 | Marek Turcáni | 06-200-18 | 12 | 22 | 0.9 | 0.1 | Eastern | 0.857 | 0.000 | 0.001 | 0.123 | 0.019 | 0.000 | Eastern |
| Slovakia | Banská Štiavnica | 48.45805556 | 18.89638889 | Nov-06 | Marek Turcáni | 06-200-19 | 12 | 22 | 0.598 | 0.402 | Mixed | 0.006 | 0.545 | 0.001 | 0.418 | 0.003 | 0.027 | Western - Moderate |
| Slovakia | Banská Štiavnica | 48.45805556 | 18.89638889 | Nov-06 | Marek Turcáni | 06-200-20 | 12 | 22 | 0.799 | 0.201 | Mixed | 0.212 | 0.019 | 0.002 | 0.734 | 0.025 | 0.007 | F2 - Moderate |
| Spain | La Langa | 40.089468 | -2.660303 |  | Adam Pepi | 15-181-01 | 13 |  | 0.076 | 0.924 | Western | 0.000 | 0.998 | 0.000 | 0.000 | 0.000 | 0.001 | Western |
| Spain | La Langa | 40.089468 | -2.660303 |  | Adam Pepi | 15-181-02 | 13 |  | 0.075 | 0.925 | Western | 0.000 | 0.993 | 0.000 | 0.001 | 0.000 | 0.006 | Western |
| Spain | La Langa | 40.089468 | -2.660303 |  | Adam Pepi | 15-181-03 | 13 |  | 0.04 | 0.96 | Western | 0.000 | 0.999 | 0.000 | 0.000 | 0.000 | 0.001 | Western |
| Spain | La Langa | 40.089468 | -2.660303 |  | Adam Pepi | 15-181-04 | 13 |  | 0.034 | 0.966 | Western | 0.000 | 0.999 | 0.000 | 0.000 | 0.000 | 0.001 | Western |
| Spain | Lugo | 43.11841667 | -7.651694444 | Dec-08 | Maria Lombardero | 1905 | 13 | 23 | 0.046 | 0.954 | Western | 0.000 | 0.999 | 0.000 | 0.000 | 0.000 | 0.001 | Western |
| Spain | Lugo | 43.11841667 | -7.651694444 | Dec-08 | Maria Lombardero | 1906 | 13 | 23 | 0.037 | 0.963 | Western | 0.000 | 0.999 | 0.000 | 0.000 | 0.000 | 0.001 | Western |
| Spain | Lugo | 43.119 | -7.649694444 | Dec-08 | Maria Lombardero | 1910 | 13 | 23 | 0.054 | 0.946 | Western | 0.000 | 0.997 | 0.000 | 0.001 | 0.000 | 0.002 | Western |
| SPAIN | Lugo | 43.11841667 | -7.651694444 | Dec-08 | Maria Lombardero | 08-320-01 | 13 | 23 | 0.055 | 0.945 | Western | 0.000 | 0.998 | 0.000 | 0.000 | 0.000 | 0.001 | Western |
| SPAIN | Lugo | 43.11841667 | -7.651694444 | Dec-08 | Maria Lombardero | 08-320-02 | 13 | 23 | 0.06 | 0.94 | Western | 0.000 | 0.999 | 0.000 | 0.000 | 0.000 | 0.001 | Western |
| SPAIN | Lugo | 43.11841667 | -7.651694444 | Dec-08 | Maria Lombardero | 08-320-03 | 13 | 23 | 0.056 | 0.944 | Western | 0.000 | 0.998 | 0.000 | 0.000 | 0.000 | 0.001 | Western |
| SPAIN | Lugo | 42.99222222 | -7.545083333 | Dec-08 | Maria Lombardero | 08-322-01 | 13 | 23 | 0.057 | 0.943 | Western | 0.000 | 0.999 | 0.000 | 0.000 | 0.000 | 0.001 | Western |
| Spain | Lugo | 42.9926 | -7.5441 | 1/9/14 | Maria Lombardero | 14-001-02 | 13 | 23 | 0.019 | 0.981 | Western | 0.000 | 1.000 | 0.000 | 0.000 | 0.000 | 0.000 | Western |
| Spain | Lugo | 42.9926 | -7.5441 | 1/9/14 | Maria Lombardero | 14-001-03 | 13 | 23 | 0.022 | 0.978 | Western | 0.000 | 1.000 | 0.000 | 0.000 | 0.000 | 0.000 | Western |
| Spain | Lugo | 42.9926 | -7.5441 | 1/9/14 | Maria Lombardero | 14-001-04 | 13 | 23 | 0.024 | 0.976 | Western | 0.000 | 1.000 | 0.000 | 0.000 | 0.000 | 0.000 | Western |
| Spain | Lugo | 42.9926 | -7.5441 | 1/9/14 | Maria Lombardero | 14-001-05 | 13 | 23 | 0.063 | 0.937 | Western | 0.000 | 0.999 | 0.000 | 0.000 | 0.000 | 0.001 | Western |
| Spain | Lugo | 42.9926 | -7.5441 | 1/9/14 | Maria Lombardero | 14-001-06 | 13 | 23 | 0.045 | 0.955 | Western | 0.000 | 0.999 | 0.000 | 0.000 | 0.000 | 0.001 | Western |
| Spain | Lugo | 42.9926 | -7.5441 | 1/9/14 | Maria Lombardero | 14-001-07 | 13 | 23 | 0.017 | 0.983 | Western | 0.000 | 1.000 | 0.000 | 0.000 | 0.000 | 0.000 | Western |
| Spain | Lugo | 42.9926 | -7.5441 | 1/9/14 | Maria Lombardero | 14-001-08 | 13 | 23 | 0.034 | 0.966 | Western | 0.000 | 0.999 | 0.000 | 0.000 | 0.000 | 0.001 | Western |
| Spain | Lugo | 42.9926 | -7.5441 | 1/9/14 | Maria Lombardero | 14-001-09 | 13 | 23 | 0.026 | 0.974 | Western | 0.000 | 1.000 | 0.000 | 0.000 | 0.000 | 0.000 | Western |
| Spain | Lugo | 42.9926 | -7.5441 | 1/9/14 | Maria Lombardero | 14-001-10 | 13 | 23 | 0.025 | 0.975 | Western | 0.000 | 1.000 | 0.000 | 0.000 | 0.000 | 0.000 | Western |
| Sweden | Upsalla | 59.81816667 | 17.65663333 | 11/24/06 | Helena Bylund | 210 | 14 | 24 | 0.835 | 0.165 | Eastern | 0.408 | 0.002 | 0.010 | 0.521 | 0.052 | 0.006 | F2 - Moderate |
| Sweden | Upsalla | 59.81816667 | 17.65663333 | 11/24/06 | Helena Bylund | 212 | 14 | 24 | 0.452 | 0.548 | Mixed | 0.001 | 0.429 | 0.014 | 0.463 | 0.002 | 0.090 | Unassigned |
| Sweden | Upsalla | 59.81816667 | 17.65663333 | 11/24/06 | Helena Bylund | 213 | 14 | 24 | 0.498 | 0.502 | Mixed | 0.000 | 0.661 | 0.019 | 0.259 | 0.002 | 0.059 | Western - Moderate |
| Sweden | Upsalla | 59.81816667 | 17.65663333 | 11/24/06 | Helena Bylund | 214 | 14 | 24 | 0.668 | 0.332 | Mixed | 0.023 | 0.049 | 0.012 | 0.871 | 0.019 | 0.027 | F2 |
| Sweden | Upsalla | 59.81816667 | 17.65663333 | 11/24/06 | Helena Bylund | 215 | 14 | 24 | 0.081 | 0.919 | Western | 0.000 | 0.997 | 0.000 | 0.001 | 0.000 | 0.002 | Western |
| Sweden | Upsalla | 59.81816667 | 17.65663333 | 11/24/06 | Helena Bylund | 216 | 14 | 24 | 0.505 | 0.495 | Mixed | 0.000 | 0.709 | 0.001 | 0.254 | 0.000 | 0.036 | Western - Moderate |
| Sweden | Upsalla | 59.81816667 | 17.65663333 | 11/24/06 | Helena Bylund | 218 | 14 | 24 | 0.137 | 0.863 | Western | 0.000 | 0.995 | 0.000 | 0.002 | 0.000 | 0.004 | Western |
| Sweden | Upsalla | 59.81816667 | 17.65663333 | 11/24/06 | Helena Bylund | 219 | 14 | 24 | 0.182 | 0.818 | Western | 0.000 | 0.982 | 0.000 | 0.010 | 0.000 | 0.007 | Western |
| Sweden | Upsalla | 59.81816667 | 17.65663333 | 10/26/13 | Helena Bylund | 5412 | 14 | 24 | 0.119 | 0.881 | Western | 0.000 | 0.994 | 0.000 | 0.002 | 0.000 | 0.003 | Western |
| Sweden | Upsalla | 59.81816667 | 17.65663333 | 10/26/13 | Helena Bylund | 5413 | 14 | 24 | 0.096 | 0.904 | Western | 0.000 | 0.998 | 0.000 | 0.000 | 0.000 | 0.001 | Western |
| Sweden | Upsalla | 59.81816667 | 17.65663333 | 10/26/13 | Helena Bylund | 5414 | 14 | 24 | 0.174 | 0.826 | Western | 0.000 | 0.989 | 0.000 | 0.004 | 0.000 | 0.007 | Western |
| Sweden | Upsalla | 59.81816667 | 17.65663333 | 10/26/13 | Helena Bylund | 5415 | 14 | 24 | 0.657 | 0.343 | Mixed | 0.041 | 0.024 | 0.006 | 0.895 | 0.015 | 0.019 | F2 |
| Sweden | Upsalla | 59.81816667 | 17.65663333 | 10/26/13 | Helena Bylund | 5416 | 14 | 24 | 0.232 | 0.768 | Mixed | 0.000 | 0.991 | 0.000 | 0.004 | 0.000 | 0.005 | Western |
| Sweden | Upsalla | 59.81816667 | 17.65663333 | 10/26/13 | Helena Bylund | 5417 | 14 | 24 | 0.232 | 0.768 | Mixed | 0.000 | 0.981 | 0.000 | 0.009 | 0.000 | 0.009 | Western |
| Sweden | Upsalla | 59.81816667 | 17.65663333 | 10/26/13 | Helena Bylund | 5418 | 14 | 24 | 0.488 | 0.512 | Mixed | 0.000 | 0.679 | 0.007 | 0.260 | 0.002 | 0.053 | Western - Moderate |
| Sweden | Upsalla | 59.81816667 | 17.65663333 | 10/26/13 | Helena Bylund | 5419 | 14 | 24 | 0.23 | 0.77 | Mixed | 0.000 | 0.952 | 0.000 | 0.021 | 0.000 | 0.027 | Western |
| Sweden | Upsalla | 59.81816667 | 17.65663333 | 10/26/13 | Helena Bylund | 5420 | 14 | 24 | 0.347 | 0.653 | Mixed | 0.000 | 0.173 | 0.002 | 0.704 | 0.000 | 0.120 | F2 - Moderate |
| Sweden | Upsalla | 59.81816667 | 17.65663333 | 10/26/13 | Helena Bylund | 5421 | 14 | 24 | 0.461 | 0.539 | Mixed | 0.000 | 0.369 | 0.000 | 0.601 | 0.001 | 0.029 | F2 - Moderate |
| Sweden | Upsalla | 59.81816667 | 17.65663333 | 10/26/13 | Helena Bylund | 13-292-01 | 14 | 24 | 0.846 | 0.154 | Eastern | 0.384 | 0.045 | 0.004 | 0.534 | 0.024 | 0.010 | F2 - Moderate |
| Sweden | Upsalla | 59.81816667 | 17.65663333 | 10/26/13 | Helena Bylund | 13-292-02 | 14 | 24 | 0.43 | 0.57 | Mixed | 0.000 | 0.231 | 0.002 | 0.739 | 0.003 | 0.024 | F2 - Moderate |
| Sweden | Upsalla | 59.81816667 | 17.65663333 | 10/26/13 | Helena Bylund | 13-292-03 | 14 | 24 | 0.424 | 0.576 | Mixed | 0.000 | 0.716 | 0.002 | 0.231 | 0.000 | 0.051 | Western - Moderate |
| Switzerland | Delémont | 47.37527778 | 7.325277778 | 11/20/08 | M. J. W. Cock | 08-316-01 | 15 | 25 | 0.399 | 0.601 | Mixed | 0.000 | 0.920 | 0.000 | 0.060 | 0.000 | 0.020 | Western |
| Switzerland | Delémont | 47.37527778 | 7.325277778 | 11/20/08 | M. J. W. Cock | 08-316-02 | 15 | 25 | 0.369 | 0.631 | Mixed | 0.000 | 0.973 | 0.000 | 0.017 | 0.000 | 0.010 | Western |
| Switzerland | Delémont | 47.37527778 | 7.325277778 | 11/20/08 | M. J. W. Cock | 08-316-03 | 15 | 25 | 0.527 | 0.473 | Mixed | 0.000 | 0.779 | 0.003 | 0.190 | 0.001 | 0.028 | Western - Moderate |
| Switzerland | Delémont | 47.37527778 | 7.325277778 | 11/20/08 | M. J. W. Cock | 08-316-04 | 15 | 25 | 0.386 | 0.614 | Mixed | 0.000 | 0.933 | 0.000 | 0.049 | 0.000 | 0.017 | Western |
| Switzerland | Delémont | 47.37527778 | 7.325277778 | 11/20/08 | M. J. W. Cock | 08-316-05 | 15 | 25 | 0.152 | 0.848 | Western | 0.000 | 0.998 | 0.000 | 0.000 | 0.000 | 0.001 | Western |
| Switzerland | Delémont | 47.37527778 | 7.325277778 | 11/20/08 | M. J. W. Cock | 08-316-06 | 15 | 25 | 0.197 | 0.803 | Western | 0.000 | 0.986 | 0.000 | 0.009 | 0.000 | 0.005 | Western |
| Switzerland | Delémont | 47.37527778 | 7.325277778 | 11/20/08 | M. J. W. Cock | 08-316-07 | 15 | 25 | 0.061 | 0.939 | Western | 0.000 | 0.999 | 0.000 | 0.000 | 0.000 | 0.001 | Western |
| Switzerland | Delémont | 47.37527778 | 7.325277778 | 11/20/08 | M. J. W. Cock | 08-316-08 | 15 | 25 | 0.439 | 0.561 | Mixed | 0.000 | 0.829 | 0.003 | 0.131 | 0.000 | 0.036 | Western |
| Switzerland | Delémont | 47.37527778 | 7.325277778 | 11/20/08 | M. J. W. Cock | 08-316-09 | 15 | 25 | 0.227 | 0.773 | Mixed | 0.000 | 0.989 | 0.000 | 0.003 | 0.000 | 0.007 | Western |
| Switzerland | Delémont | 47.37527778 | 7.325277778 | 11/20/08 | M. J. W. Cock | 08-316-10 | 15 | 25 | 0.065 | 0.935 | Western | 0.000 | 0.999 | 0.000 | 0.000 | 0.000 | 0.001 | Western |
| Switzerland | Delémont | 47.37527778 | 7.325277778 | 11/20/08 | M. J. W. Cock | 08-316-11 | 15 | 25 | 0.499 | 0.501 | Mixed | 0.000 | 0.795 | 0.000 | 0.184 | 0.000 | 0.020 | Western - Moderate |
| Switzerland | Delémont | 47.37527778 | 7.325277778 | 11/20/08 | M. J. W. Cock | 08-316-12 | 15 | 25 | 0.321 | 0.679 | Mixed | 0.000 | 0.936 | 0.000 | 0.042 | 0.000 | 0.022 | Western |
| Switzerland | Delémont | 47.37527778 | 7.325277778 | 11/20/08 | M. J. W. Cock | 08-316-13 | 15 | 25 | 0.452 | 0.548 | Mixed | 0.000 | 0.822 | 0.000 | 0.154 | 0.000 | 0.024 | Western |
| Switzerland | Delémont | 47.37527778 | 7.325277778 | 11/20/08 | M. J. W. Cock | 08-316-14 | 15 | 25 | 0.218 | 0.782 | Mixed | 0.000 | 0.992 | 0.000 | 0.003 | 0.000 | 0.005 | Western |
| Switzerland | Delémont | 47.37527778 | 7.325277778 | 11/20/08 | M. J. W. Cock | 08-316-15 | 15 | 25 | 0.131 | 0.869 | Western | 0.000 | 0.998 | 0.000 | 0.000 | 0.000 | 0.002 | Western |
| Switzerland | Delémont | 47.365719 | 7.346482 | 11/13/13 | Marc Kenis | 13-294-02 | 15 | 25 | 0.37 | 0.63 | Mixed | 0.001 | 0.761 | 0.001 | 0.208 | 0.001 | 0.029 | Western - Moderate |
| Switzerland | Delémont | 47.365719 | 7.346482 | 11/13/13 | Marc Kenis | 13-294-03 | 15 | 25 | 0.394 | 0.606 | Mixed | 0.000 | 0.244 | 0.002 | 0.728 | 0.002 | 0.025 | F2 - Moderate |
| Switzerland | Delémont | 47.365719 | 7.346482 | 11/13/13 | Marc Kenis | 13-294-05 | 15 | 25 | 0.16 | 0.84 | Western | 0.000 | 0.983 | 0.000 | 0.011 | 0.000 | 0.006 | Western |
| Switzerland | Delémont | 47.365719 | 7.346482 | 11/13/13 | Marc Kenis | 13-294-06 | 15 | 25 | 0.197 | 0.803 | Western | 0.000 | 0.986 | 0.000 | 0.007 | 0.000 | 0.007 | Western |
| Switzerland | Delémont | 47.365719 | 7.346482 | 11/13/13 | Marc Kenis | 13-295-01 | 15 | 25 | 0.06 | 0.94 | Western | 0.000 | 0.999 | 0.000 | 0.000 | 0.000 | 0.001 | Western |
| Switzerland | Delémont | 47.365719 | 7.346482 | 11/13/13 | Marc Kenis | 13-295-02 | 15 | 25 | 0.348 | 0.652 | Mixed | 0.000 | 0.889 | 0.001 | 0.087 | 0.000 | 0.023 | Western |
| Switzerland | Malettes | 47.38555556 | 7.201944444 | 11/20/08 | M. J. W. Cock | 08-319-01 | 15 | 26 | 0.45 | 0.55 | Mixed | 0.000 | 0.753 | 0.007 | 0.199 | 0.001 | 0.039 | Western - Moderate |
| Switzerland | Malettes | 47.38555556 | 7.201944444 | 11/20/08 | M. J. W. Cock | 08-319-02 | 15 | 26 | 0.217 | 0.783 | Mixed | 0.000 | 0.984 | 0.000 | 0.008 | 0.000 | 0.008 | Western |
| Switzerland | Malettes | 47.38555556 | 7.201944444 | 11/20/08 | M. J. W. Cock | 08-319-03 | 15 | 26 | 0.213 | 0.787 | Mixed | 0.000 | 0.989 | 0.000 | 0.005 | 0.000 | 0.006 | Western |
| Switzerland | Malettes | 47.38555556 | 7.201944444 | 11/20/08 | M. J. W. Cock | 08-319-04 | 15 | 26 | 0.421 | 0.579 | Mixed | 0.000 | 0.917 | 0.000 | 0.067 | 0.000 | 0.015 | Western |
| Switzerland | Malettes | 47.38555556 | 7.201944444 | 11/20/08 | M. J. W. Cock | 08-319-05 | 15 | 26 | 0.101 | 0.899 | Western | 0.000 | 0.998 | 0.000 | 0.001 | 0.000 | 0.002 | Western |
| Switzerland | Malettes | 47.38555556 | 7.201944444 | 11/20/08 | M. J. W. Cock | 08-319-06 | 15 | 26 | 0.342 | 0.658 | Mixed | 0.000 | 0.784 | 0.001 | 0.140 | 0.000 | 0.075 | Western - Moderate |
| Switzerland | Malettes | 47.38555556 | 7.201944444 | 11/20/08 | Marc Kenis | 08-319-07 | 15 | 26 | 0.053 | 0.947 | Western | 0.000 | 0.999 | 0.000 | 0.000 | 0.000 | 0.001 | Western |
| Switzerland | Malettes | 47.38555556 | 7.201944444 | 11/20/08 | Marc Kenis | 08-319-08 | 15 | 26 | 0.254 | 0.746 | Mixed | 0.000 | 0.984 | 0.000 | 0.008 | 0.000 | 0.008 | Western |
| Switzerland | Malettes | 47.38555556 | 7.201944444 | 11/20/08 | Marc Kenis | 08-319-09 | 15 | 26 | 0.171 | 0.829 | Western | 0.000 | 0.995 | 0.000 | 0.001 | 0.000 | 0.004 | Western |
| Switzerland | Malettes | 47.38555556 | 7.201944444 | 11/20/08 | Marc Kenis | 08-319-10 | 15 | 26 | 0.738 | 0.262 | Mixed | 0.046 | 0.170 | 0.012 | 0.723 | 0.022 | 0.028 | F2 - Moderate |
| Switzerland | Malettes | 47.38555556 | 7.201944444 | 11/20/08 | Marc Kenis | 08-319-11 | 15 | 26 | 0.141 | 0.859 | Western | 0.000 | 0.997 | 0.000 | 0.001 | 0.000 | 0.002 | Western |
| Switzerland | Malettes | 47.38555556 | 7.201944444 | 11/20/08 | Marc Kenis | 08-319-12 | 15 | 26 | 0.227 | 0.773 | Mixed | 0.000 | 0.989 | 0.000 | 0.006 | 0.000 | 0.006 | Western |
| Switzerland | Malettes | 47.38555556 | 7.201944444 | 11/20/08 | Marc Kenis | 08-319-13 | 15 | 26 | 0.075 | 0.925 | Western | 0.000 | 0.997 | 0.000 | 0.001 | 0.000 | 0.002 | Western |
| Switzerland | Malettes | 47.38555556 | 7.201944444 | 11/20/08 | Marc Kenis | 08-319-14 | 15 | 26 | 0.431 | 0.569 | Mixed | 0.000 | 0.414 | 0.003 | 0.503 | 0.001 | 0.080 | F2 - Moderate |
| Switzerland | Malettes | 47.38555556 | 7.201944444 | 11/20/08 | Marc Kenis | 08-319-15 | 15 | 26 | 0.127 | 0.873 | Western | 0.000 | 0.994 | 0.000 | 0.002 | 0.000 | 0.004 | Western |
| Switzerland | Malettes | 47.38555556 | 7.201944444 | 11/20/08 | Marc Kenis | 08-319-16 | 15 | 26 | 0.49 | 0.51 | Mixed | 0.001 | 0.573 | 0.001 | 0.398 | 0.001 | 0.026 | Western - Moderate |
| Switzerland | Malettes | 47.38555556 | 7.201944444 | 11/20/08 | Marc Kenis | 08-319-17 | 15 | 26 | 0.32 | 0.68 | Mixed | 0.000 | 0.966 | 0.000 | 0.020 | 0.000 | 0.013 | Western |
| Switzerland | Malettes | 47.38555556 | 7.201944444 | 11/20/08 | Marc Kenis | 08-319-18 | 15 | 26 | 0.162 | 0.838 | Western | 0.000 | 0.993 | 0.000 | 0.003 | 0.000 | 0.004 | Western |
| Switzerland | Malettes | 47.38555556 | 7.201944444 | 11/20/08 | Marc Kenis | 08-319-19 | 15 | 26 | 0.368 | 0.632 | Mixed | 0.000 | 0.963 | 0.000 | 0.025 | 0.000 | 0.011 | Western |
| Switzerland | Pleigne | 47.41861111 | 7.246388889 | 12/4/08 | M. J. W. Cock | 08-318-01 | 15 |  | 0.523 | 0.477 | Mixed | 0.005 | 0.480 | 0.000 | 0.495 | 0.001 | 0.019 | F2 - Moderate |
| Switzerland | Pleigne | 47.41861111 | 7.246388889 | 12/4/08 | M. J. W. Cock | 08-318-02 | 15 |  | 0.278 | 0.722 | Mixed | 0.000 | 0.976 | 0.000 | 0.016 | 0.000 | 0.008 | Western |
| Switzerland | Pleigne | 47.41861111 | 7.246388889 | 12/4/08 | M. J. W. Cock | 08-318-03 | 15 |  | 0.486 | 0.514 | Mixed | 0.001 | 0.662 | 0.003 | 0.302 | 0.002 | 0.031 | Western - Moderate |
| Switzerland | Pleigne | 47.41861111 | 7.246388889 | 12/4/08 | M. J. W. Cock | 08-318-04 | 15 |  | 0.251 | 0.749 | Mixed | 0.000 | 0.916 | 0.000 | 0.069 | 0.000 | 0.014 | Western |
| Switzerland | Pleigne | 47.41861111 | 7.246388889 | 12/4/08 | M. J. W. Cock | 08-318-05 | 15 |  | 0.171 | 0.829 | Western | 0.000 | 0.980 | 0.000 | 0.011 | 0.000 | 0.009 | Western |
| Switzerland | Pleigne | 47.41861111 | 7.246388889 | 12/4/08 | M. J. W. Cock | 08-318-06 | 15 |  | 0.281 | 0.719 | Mixed | 0.000 | 0.947 | 0.000 | 0.041 | 0.000 | 0.012 | Western |
| Switzerland | Pleigne | 47.41861111 | 7.246388889 | 12/4/08 | M. J. W. Cock | 08-318-07 | 15 |  | 0.138 | 0.862 | Western | 0.000 | 0.993 | 0.000 | 0.002 | 0.000 | 0.005 | Western |
| Switzerland | Pleigne | 47.41861111 | 7.246388889 | 12/4/08 | M. J. W. Cock | 08-318-08 | 15 |  | 0.273 | 0.727 | Mixed | 0.000 | 0.960 | 0.001 | 0.026 | 0.000 | 0.013 | Western |
| Switzerland | Pleigne | 47.41861111 | 7.246388889 | 12/4/08 | M. J. W. Cock | 08-318-09 | 15 |  | 0.784 | 0.216 | Mixed | 0.084 | 0.016 | 0.037 | 0.791 | 0.053 | 0.018 | F2 - Moderate |

**Table S2.** DIYABC parameter priors, distributions, and constraints.

| Parameter | Distribution | Minimum | Maximum | Mean | st-dev |
| --- | --- | --- | --- | --- | --- |
| N1 | Normal | 10 | 4 x 10^4^ | 2 x 10^4^ | 1 x 10^4^ |
| N2 | Normal | 10 | 1 x 10^5^ | 5 x 10^4^ | 2.5 x 10^4^ |
| N3 | Normal | 10 | 4 x 10^4^ | 2 x 10^4^ | 1 x 10^4^ |
| N4 | Normal | 10 | 1 x 10^5^ | 5 x 10^4^ | 2.5 x 10^4^ |
| NA1 | Normal | 10 | 4 x 10^4^ | 2 x 10^4^ | 1 x 10^4^ |
| NA3 | Uniform | 10 | 8 x 10^4^ | na | na |
| NA5 | Uniform | 10 | 1 x 10^4^ | na | na |
| t1 | Uniform | 10 | 4 x 10^4^ | na | na |
| t2 | Uniform | 10 | 3 x 10^4^ | na | na |
| t3 | Normal | 10 | 6 x 10^4^ | na | na |
| ra | Uniform | 0.001 | 0.999 | na | na |
| Constrains: t3>t2, t3>t1, t2>t1 | | |  |  |  |

**Table S3.** Population pairwise estimates of *F*_ST_ corrected for the presence of null-alleles calculated in FREENA (lower diagonal) and *D*_EST_ in SMOGD (upper diagonal).

**Table S4.** Mean migration rates and standard errors (SE) averaged across four independent BayesAss Analyses. Results are reported as follows. Migration rates from country *i* to country *j* = m[*i*][*j*]. Migration rates whose 95% confidence intervals (mean ± 1.96 x SE) do not include 0 are highlighted in bold.

|  | | | | | | | | | | |
| --- | --- | --- | --- | --- | --- | --- | --- | --- | --- | --- |
| Country Code | Country | Migration | Mean | SE | Migration | Mean | SE | Migration | Mean | SE |
| 0 | Austria | m[0][0] | 0.781925 | 0.044925 | m[0][1] | 0.004275 | 0.0042 | m[0][4] | 0.004275 | 0.004225 |
| 1 | Czech Republic | m[1][0] | **0.084075** | 0.02775 | m[1][1] | 0.676475 | 0.009575 | m[1][4] | 0.009825 | 0.00955 |
| 4 | Georgia | m[4][0] | 0.008025 | 0.00785 | m[4][1] | 0.0079 | 0.0076 | m[4][4] | 0.888525 | 0.023925 |
| 8 | Poland | m[8][0] | **0.200025** | 0.040525 | m[8][1] | 0.007675 | 0.007475 | m[8][4] | 0.007625 | 0.0075 |
| 10 | Serbia | m[10][0] | **0.183775** | 0.069775 | m[10][1] | 0.0051 | 0.005 | m[10][4] | 0.004975 | 0.004875 |
| 11 | Slovakia | m[11][0] | **0.1785** | 0.02955 | m[11][1] | 0.0095 | 0.009325 | m[11][4] | 0.0095 | 0.009225 |
| 2 | England | m[2][0] | 0.005375 | 0.00525 | m[2][1] | 0.005475 | 0.005325 | m[2][4] | 0.005425 | 0.00535 |
| 3 | France | m[3][0] | 0.003025 | 0.003 | m[3][1] | 0.002375 | 0.0024 | m[3][4] | 0.0024 | 0.00235 |
| 5 | Germany | m[5][0] | 0.003375 | 0.003325 | m[5][1] | 0.002725 | 0.002625 | m[5][4] | 0.002675 | 0.002675 |
| 6 | Italy | m[6][0] | 0.019325 | 0.018425 | m[6][1] | 0.01115 | 0.0107 | m[6][4] | 0.0112 | 0.010775 |
| 7 | Norway | m[7][0] | 0.0084 | 0.0081 | m[7][1] | 0.0073 | 0.0071 | m[7][4] | 0.0072 | 0.007175 |
| 9 | Scotland | m[9][0] | 0.0063 | 0.00605 | m[9][1] | 0.005975 | 0.00595 | m[9][4] | 0.00605 | 0.0059 |
| 12 | Spain | m[12][0] | 0.0096 | 0.009275 | m[12][1] | 0.009575 | 0.0093 | m[12][4] | 0.009375 | 0.009025 |
| 13 | Sweden | m[13][0] | 0.023325 | 0.01785 | m[13][1] | 0.009275 | 0.009 | m[13][4] | 0.0093 | 0.008975 |
| 14 | Switzerland | m[14][0] | 0.005575 | 0.005425 | m[14][1] | 0.004975 | 0.004875 | m[14][4] | 0.004975 | 0.00495 |
|  |  |  |  |  |  |  |  |  |  |  |
| Country Code | Country | Migration | Mean | SE | Migration | Mean | SE | Migration | Mean | SE |
| 0 | Austria | m[0][8] | 0.004225 | 0.0042 | m[0][10] | 0.004575 | 0.004625 | m[0][11] | 0.00425 | 0.004175 |
| 1 | Czech Republic | m[1][8] | 0.009825 | 0.0095 | m[1][10] | 0.014225 | 0.012175 | m[1][11] | 0.0098 | 0.009525 |
| 4 | Georgia | m[4][8] | 0.00805 | 0.007875 | m[4][10] | 0.008025 | 0.00795 | m[4][11] | 0.00805 | 0.00785 |
| 8 | Poland | m[8][8] | 0.6773 | 0.016025 | m[8][10] | 0.03025 | 0.0276 | m[8][11] | 0.007675 | 0.0075 |
| 10 | Serbia | m[10][8] | 0.005 | 0.004925 | m[10][10] | 0.74165 | 0.0698 | m[10][11] | 0.004975 | 0.0049 |
| 11 | Slovakia | m[11][8] | 0.0095 | 0.009275 | m[11][10] | 0.016375 | 0.0148 | m[11][11] | 0.6762 | 0.00925 |
| 2 | England | m[2][8] | 0.00535 | 0.0053 | m[2][10] | 0.005425 | 0.00525 | m[2][11] | 0.005425 | 0.005325 |
| 3 | France | m[3][8] | 0.002375 | 0.002375 | m[3][10] | 0.002325 | 0.0023 | m[3][11] | 0.002325 | 0.0024 |
| 5 | Germany | m[5][8] | 0.002825 | 0.0028 | m[5][10] | 0.002675 | 0.00275 | m[5][11] | 0.002725 | 0.0027 |
| 6 | Italy | m[6][8] | 0.011075 | 0.0107 | m[6][10] | 0.011075 | 0.0107 | m[6][11] | 0.011225 | 0.0109 |
| 7 | Norway | m[7][8] | 0.007325 | 0.00695 | m[7][10] | 0.00745 | 0.007425 | m[7][11] | 0.0074 | 0.007125 |
| 9 | Scotland | m[9][8] | 0.00605 | 0.00595 | m[9][10] | 0.00605 | 0.006 | m[9][11] | 0.006175 | 0.005975 |
| 12 | Spain | m[12][8] | 0.0095 | 0.00925 | m[12][10] | 0.0096 | 0.0093 | m[12][11] | 0.009425 | 0.00915 |
| 13 | Sweden | m[13][8] | 0.009325 | 0.00905 | m[13][10] | 0.00925 | 0.009 | m[13][11] | 0.009375 | 0.009125 |
| 14 | Switzerland | m[14][8] | 0.004925 | 0.004875 | m[14][10] | 0.0051 | 0.005025 | m[14][11] | 0.005 | 0.004925 |
|  |  |  |  |  |  |  |  |  |  |  |
| Country Code | Country | Migration | Mean | SE | Migration | Mean | SE | Migration | Mean | SE |
| 0 | Austria | m[0][2] | 0.004225 | 0.0042 | m[0][3] | 0.0067 | 0.00675 | m[0][5] | **0.160175** | 0.0476 |
| 1 | Czech Republic | m[1][2] | 0.009825 | 0.00945 | m[1][3] | 0.037475 | 0.0207 | m[1][5] | **0.0898** | 0.031375 |
| 4 | Georgia | m[4][2] | 0.007875 | 0.007725 | m[4][3] | 0.00805 | 0.007725 | m[4][5] | 0.00775 | 0.0076 |
| 8 | Poland | m[8][2] | 0.00765 | 0.00745 | m[8][3] | 0.007725 | 0.007525 | m[8][5] | 0.008125 | 0.007975 |
| 10 | Serbia | m[10][2] | 0.0051 | 0.0051 | m[10][3] | 0.005475 | 0.005475 | m[10][5] | 0.0138 | 0.00915 |
| 11 | Slovakia | m[11][2] | 0.009525 | 0.0093 | m[11][3] | 0.00975 | 0.00945 | m[11][5] | 0.02405 | 0.01605 |
| 2 | England | m[2][2] | 0.672175 | 0.0056 | m[2][3] | **0.224975** | 0.023075 | m[2][5] | 0.0118 | 0.008775 |
| 3 | France | m[3][2] | 0.002325 | 0.00235 | m[3][3] | 0.887775 | 0.0311 | m[3][5] | **0.078575** | 0.0315 |
| 5 | Germany | m[5][2] | 0.002725 | 0.002725 | m[5][3] | **0.042325** | 0.016825 | m[5][5] | 0.9208 | 0.01885 |
| 6 | Italy | m[6][2] | 0.01125 | 0.0109 | m[6][3] | **0.0866** | 0.0285 | m[6][5] | **0.09305** | 0.032225 |
| 7 | Norway | m[7][2] | 0.007525 | 0.00725 | m[7][3] | 0.015625 | 0.0117 | m[7][5] | 0.01935 | 0.01325 |
| 9 | Scotland | m[9][2] | 0.005925 | 0.0059 | m[9][3] | 0.070025 | 0.0572 | m[9][5] | 0.007875 | 0.00725 |
| 12 | Spain | m[12][2] | 0.00965 | 0.00945 | m[12][3] | 0.0241 | 0.01625 | m[12][5] | 0.009725 | 0.0095 |
| 13 | Sweden | m[13][2] | 0.009375 | 0.00905 | m[13][3] | **0.068275** | 0.0262 | m[13][5] | **0.127875** | 0.0332 |
| 14 | Switzerland | m[14][2] | 0.00495 | 0.004825 | m[14][3] | **0.087625** | 0.0276 | m[14][5] | **0.1805** | 0.030475 |
|  |  |  |  |  |  |  |  |  |  |  |
| Country Code | Country | Migration | Mean | SE | Migration | Mean | SE | Migration | Mean | SE |
| 0 | Austria | m[0][6] | 0.004175 | 0.004175 | m[0][7] | 0.004275 | 0.004225 | m[0][9] | 0.00425 | 0.0042 |
| 1 | Czech Republic | m[1][6] | 0.009775 | 0.0095 | m[1][7] | 0.009825 | 0.009475 | m[1][9] | 0.0098 | 0.009475 |
| 4 | Georgia | m[4][6] | 0.00795 | 0.007625 | m[4][7] | 0.0078 | 0.0078 | m[4][9] | 0.0078 | 0.0076 |
| 8 | Poland | m[8][6] | 0.0076 | 0.00745 | m[8][7] | 0.007625 | 0.007425 | m[8][9] | 0.007625 | 0.007425 |
| 10 | Serbia | m[10][6] | 0.005 | 0.004925 | m[10][7] | 0.00515 | 0.004975 | m[10][9] | 0.005025 | 0.00495 |
| 11 | Slovakia | m[11][6] | 0.009575 | 0.00935 | m[11][7] | 0.00945 | 0.0092 | m[11][9] | 0.0096 | 0.009325 |
| 2 | England | m[2][6] | 0.0054 | 0.005275 | m[2][7] | 0.0054 | 0.005325 | m[2][9] | 0.031625 | 0.0164 |
| 3 | France | m[3][6] | 0.002375 | 0.00225 | m[3][7] | 0.00255 | 0.0025 | m[3][9] | 0.0042 | 0.003775 |
| 5 | Germany | m[5][6] | 0.00275 | 0.002675 | m[5][7] | 0.00275 | 0.00275 | m[5][9] | 0.003375 | 0.003325 |
| 6 | Italy | m[6][6] | 0.677825 | 0.0108 | m[6][7] | 0.011125 | 0.01075 | m[6][9] | 0.011875 | 0.01145 |
| 7 | Norway | m[7][6] | 0.00745 | 0.007225 | m[7][7] | 0.8641 | 0.025675 | m[7][9] | 0.01875 | 0.012325 |
| 9 | Scotland | m[9][6] | 0.006025 | 0.005925 | m[9][7] | 0.006225 | 0.006125 | m[9][9] | 0.848925 | 0.059825 |
| 12 | Spain | m[12][6] | 0.00935 | 0.0091 | m[12][7] | 0.009525 | 0.009275 | m[12][9] | 0.018475 | 0.014 |
| 13 | Sweden | m[13][6] | 0.009325 | 0.00905 | m[13][7] | 0.009325 | 0.009125 | m[13][9] | 0.01155 | 0.0106 |
| 14 | Switzerland | m[14][6] | 0.00495 | 0.0049 | m[14][7] | 0.00495 | 0.0049 | m[14][9] | 0.005 | 0.004925 |
|  |  |  |  |  |  |  |  |  |  |  |
| Country Code | Country | Migration | Mean | SE | Migration | Mean | SE | Migration | Mean | SE |
| 0 | Austria | m[0][12] | 0.004175 | 0.004075 | m[0][13] | 0.004325 | 0.00425 | m[0][14] | 0.004175 | 0.00405 |
| 1 | Czech Republic | m[1][12] | 0.0098 | 0.009425 | m[1][13] | 0.009775 | 0.009575 | m[1][14] | 0.009775 | 0.0095 |
| 4 | Georgia | m[4][12] | 0.00805 | 0.00785 | m[4][13] | 0.0081 | 0.00785 | m[4][14] | 0.007975 | 0.0078 |
| 8 | Poland | m[8][12] | 0.0077 | 0.007525 | m[8][13] | 0.007625 | 0.00745 | m[8][14] | 0.0077 | 0.0075 |
| 10 | Serbia | m[10][12] | 0.00495 | 0.004975 | m[10][13] | 0.005 | 0.004925 | m[10][14] | 0.005025 | 0.00495 |
| 11 | Slovakia | m[11][12] | 0.009475 | 0.009275 | m[11][13] | 0.009475 | 0.00925 | m[11][14] | 0.009525 | 0.00925 |
| 2 | England | m[2][12] | 0.0054 | 0.0053 | m[2][13] | 0.005425 | 0.0053 | m[2][14] | 0.0054 | 0.005325 |
| 3 | France | m[3][12] | 0.0027 | 0.002575 | m[3][13] | 0.002375 | 0.002325 | m[3][14] | 0.0023 | 0.002225 |
| 5 | Germany | m[5][12] | 0.002725 | 0.002675 | m[5][13] | 0.00265 | 0.00265 | m[5][14] | 0.0028 | 0.002775 |
| 6 | Italy | m[6][12] | 0.0111 | 0.010725 | m[6][13] | 0.01105 | 0.010675 | m[6][14] | 0.0111 | 0.0108 |
| 7 | Norway | m[7][12] | 0.00735 | 0.007025 | m[7][13] | 0.007425 | 0.00735 | m[7][14] | 0.0074 | 0.0072 |
| 9 | Scotland | m[9][12] | 0.0061 | 0.005925 | m[9][13] | 0.006 | 0.0059 | m[9][14] | 0.006175 | 0.006075 |
| 12 | Spain | m[12][12] | 0.843 | 0.0283 | m[12][13] | 0.009525 | 0.009325 | m[12][14] | 0.009525 | 0.009225 |
| 13 | Sweden | m[13][12] | 0.009325 | 0.0091 | m[13][13] | 0.675925 | 0.009025 | m[13][14] | 0.0093 | 0.009025 |
| 14 | Switzerland | m[14][12] | 0.004975 | 0.0049 | m[14][13] | 0.004975 | 0.004875 | m[14][14] | 0.671625 | 0.004875 |

**Table S5. Results from the Evanno *et al*. method as implemented in STRUCTURE HARVESTER.**

| # K | Reps | Mean LnP(K) | Stdev LnP(K) | Ln'(K) | \|Ln''(K)\| | Delta K |
| --- | --- | --- | --- | --- | --- | --- |
| 1 | 10 | -60596.14 | 0.17 | NA | NA | NA |
| **2** | **10** | **-58737.64** | **0.98** | **1858.5** | **992** | **1009.89** |
| 3 | 10 | -57871.14 | 92.7 | 866.5 | 198.08 | 2.14 |
| 4 | 10 | -57202.72 | 205.44 | 668.42 | 199.07 | 0.97 |
| 5 | 10 | -56733.37 | 64.84 | 469.35 | 14.82 | 0.23 |
| 6 | 10 | -56278.84 | 6.31 | 454.53 | — | — |

**Supplemental Figures**

**Fig. S1.** Winter moth sample locations and proportional assignment to genetic clusters. Charts are scaled to represent the number of individuals successfully genotyped at each locality, and were generated in ARCMAP v.10.3.1 and visualized using the Europe Albers Equal Area Conic projection. A scale bar is provided in the lower right corner. The proportional shading of each chart represents the number of individuals assigned to either Cluster 1 with *Q* ≥ 0.8 (Black), Cluster 2 with *Q* ≥ 0.8 (White), or receiving mixed assignment (0.2 < *Q* < 0.8). Charts are centred approximately on their sample locality, except in instances where multiple charts would have overlapped and a line is drawn connect the chart to its sample locality. Specific information for each collection locality, including the number of individuals, the collector, the date of collection, and GPS information, is provided in Table S1.

**Fig. S2.** DIYABC Scenarios. Pop 1 = Georgia, Pop 2 = Serbia, Pop 3 = Spain, Pop 4 = Germany.

**Fig. S3.** Comparison of DIYABC Scenarios using Direct and Logistic Regression analyses. For the Direct comparison, values along the y-axis represent the proportion of simulated datasets from each scenario “closest” to the sample dataset, and for the Logistic Regression approach, values along the y-axis represent the probability of deviations between summary statistics calculated for the simulated datasets and the sample dataset. For both methods, values along the x-axis represent the distance from the sample dataset.

**Fig. S4.** Generalized additive models (GAM) comparing measures of genetic diversity to latitude. For each comparison, latitude is plot along the x-axis and the linear predictor of each measure of genetic diversity is ploted along the y-axis. The solid line represents the mean value, with dashed lines representing 95% confidence intervals. Of the four measures, only the effective number of alleles showed a marginally-significant relationship (P=0.0838).

**Figure S5.** Results from the Evanno *et al*. method as implemented in STRUCTURE HARVESTER.

**Figure S6.** Probability of assignment (*Q*) of individual winter moths to distinct genetic clusters, organized by country of collection. Proportional assignment of individuals to each of *K*=2 reconstructed genetic clusters using Structure. The results from ten independent analyses for *K*=2 were summarized with CLUMPP, and visualized in R. Individual columns represent the proportional assignment (*Q*; y-axis) of sampled winter moth individuals to either Cluster 1 (dark gray) or Cluster 2 (light gray). Moths have been grouped by the Eurasian countries from which they were sampled.

**
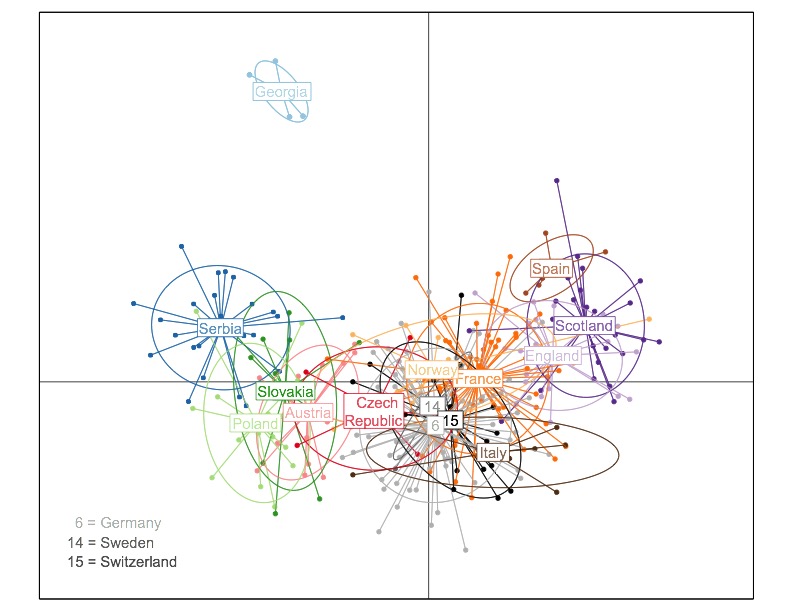
**

**Figure S7.** Results from DAPC analysis. Scatter plot showing the results from DAPC. Individual Eurasian countries are uniquely coloured to highlight the coordinates for individual moths, and a label representing each country has been placed in the centre of the inertia ellipse that includes sampled winter moths from that country. Forty principle components (PCs) were retained, as well as 14 discriminate factors. Labels for three countries whose centres of their inertia ellipses had a high amount of overlap are shown in the bottom left corner.
